# Zinc Exposure Causes Disulfidptosis to Induce Miscarriage by Up‐Regulating GATA1/METTL1/SLC7A11 Axis

**DOI:** 10.1002/advs.202514513

**Published:** 2026-05-07

**Authors:** Wenxin Huang, Yi Sun, Yanxin Wang, Haijun Yan, Xiaoping Yue, Weidong Wu, Xueyu Chen, Jinfang Zhang, Yanbing Lin, Qiong Lei, Nan Ji, Shuaishuai Xing, Liqin Zeng, Qingzheng Kang, Depeng Zhao, Geng Guo, Huidong Zhang

**Affiliations:** ^1^ Research Center for Environment and Female Reproductive Health Biological Laboratory of Hetao Cooperation Zone the Eighth Affiliated Hospital Sun Yat‐sen University Shenzhen China; ^2^ Institute of Flow Chemistry and Engineering College of Chemistry and Materials Jiangxi Normal University Nanchang Jiangxi P. R. China; ^3^ School of Public Health Henan Medical University Xinxiang Henan Province China; ^4^ Department of Reproductive Medicine Women and Children's Medical Center Shenzhen School of Clinical Medicine Shenzhen Maternity and Child Healthcare Hospital Southern Medical University Shenzhen Guangdong Province China; ^5^ Cancer Center Shenzhen Hospital (Futian) of Guangzhou University of Chinese Medicine Shenzhen China; ^6^ Department of Obstetrics The Eighth Affiliated Hospital Sun Yat‐sen University Shenzhen Guangdong Province China; ^7^ Faculty of Health Sciences Building University of Macau Taipa Macau China; ^8^ Department of Emergency Cerebrovascular Disease Center First Hospital of Shanxi Medical University Taiyuan China

**Keywords:** disulfidptosis, m7G RNA modification, SLC7A11, unexplained miscarriage, Zn exposure

## Abstract

The pathogenesis of unexplained miscarriage (UM) is largely unclear. Environmental Zn pollution is widely present in various environments and inevitably ingested by pregnant women, which has shown reproductive toxicity. Disulfidptosis is a newly identified programmed cell death caused by excessive disulfide stress. Notably, the association, causation, and underlying mechanisms among Zn exposure, disulfidptosis, and unexplained miscarriage are completely unknown. In this study, based on two UM case‐control groups, four ZnCl_2_‐exposed mouse models, and a ZnCl_2_‐exposed trophoblast Swan 71 cell model, we obtain a consistent conclusion that excessive Zn exposure causes disulfidptosis and thus induces miscarriage by up‐regulating the GATA1/METTL1/SLC7A11 axis. In the mechanism, Zn exposure up‐regulates GATA1 expression levels, which promotes GATA1‐mediated METTL1 and SLC7A11 transcription. Meanwhile, Zn exposure also promotes METTL1‐mediated m7G modification on SLC7A11 mRNA and thus increases SLC7A11 mRNA stability. Ultimately, Zn exposure up‐regulates SLC7A11 expression levels at both transcription and post‐transcription levels and thus causes disulfidptosis. Knockdown of murine Slc7a11, Gata1, or Mettl1, or supplement with NADPH suppresses mouse placental disulfidptosis and alleviates mouse miscarriage. This study not only discovers pathogenesis and biological mechanisms of Zn exposure‐induced unexplained miscarriage but also provides potential targets, uncovering new health risk effects of Zn exposure in the environment‐health field.

## Graphical abstract

Zn exposure up‐regulates GATA1, promoting GATA1‐mediated METTL1 and SLC7A11 transcription. It also enhances METTL1‐mediated m7G modification on SLC7A11 mRNA, increasing SLC7A11 mRNA stability. Ultimately, Zn exposure up‐regulates SLC7A11 at both transcriptional and post‐transcriptional levels, causing disulfidptosis. Knockdown of murine Slc7a11, Gata1, or Mettl1, or supplementation with NADPH, suppresses mouse placental disulfidptosis and alleviates mouse miscarriage.

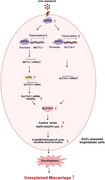

## 1 Introduction

First‐trimester miscarriage (or early pregnancy loss) is the most common complication in pregnancy [1, 2]. Approximately 15%–25% of pregnant women in the world experience miscarriage; and 1%–5% suffer from recurrent miscarriage [3], which greatly limits global human reproduction and impairs family happiness. Moreover, the incidence of miscarriage is rising in recent years [4]. Many factors might induce or cause miscarriage, including chromosomal abnormalities, uterine deformation, hormonal abnormalities, infections, psychological trauma and stressful life events, and immune disorders [5]. However, there are still 50% causes unidentified [6], which are generally identified as unexplained miscarriage (UM). The unclear pathogenesis of UM has becoming important stumbling block for clinical treatment against UM.

Recently, increasing evidence has demonstrated that environmental toxicants might induce UM, such as polycyclic aromatic hydrocarbon BaP/BPDE [7, 8, 9, 10, 11, 12, 13, 14], nanoplastics [15, 16], hypoxia [17], or disinfection by‐products of tap water [18]. In addition to these, zinc (Zn) pollution is widely present in various environments, which is originated from mining of Zn ores and metallurgic operations, the electroplating industry, the non‐ferrous metal processing industry, and highway pollution emission [19, 20]. For example, the levels of Zn exposure are 1979 mg/kg in soil at 185 industrial legacies [19], 0.162 mg/L emitted in Portuguese Highway in 2019 [20], and 37–197 mg/kg in sediment samples on water resources in Vatukoula Goldmine region (VGR), Fiji [21]. Human, including pregnant women, might contact and accumulate high levels of Zn through foods, drinking water, and air inhalation. It has been reported that the median intake content of Zn is 0.243 mg/kg/day (base on Zn element) in the urine of male residents in Chongqing, China [22]. The content of Zn has been detected as 0.71 mg/g creatinine in term birth maternal urine and 12.6 mg/g creatinine in preterm birth maternal urine of pregnant women in Wuhan, China [23], 1.48 mg/L in cord whole blood of pregnant women in Guangdong, China [24], and 665 µg/L in serum of 406 adolescent‐neonate dyads [25]. Although Zn is an essential trace element in the human body and participates in multiple cellular processes [26], exposure to high levels of Zn might cause adverse effects on human. For example, intake of metallic dietary supplements containing 300 mg Zn per day might induce anemia, neutropenia, and immune suppression [22]. Epidemiological studies have shown that Zn exposure during early pregnancy and its higher contents in urine samples are associated with a higher risk of adverse pregnancy outcomes, such as preterm birth [23, 27]. Animal model studies also show that high levels of Zn exposure damage spermatogenic cells and increase abnormal sperm morphology in male mice, and reduce sperm count and motility in male rats [28]. ZnO nanoparticles (NPs), which release Zn ions from NPs [29], inhibit follicle growth, limit oocyte differentiation, damage ovarian cells, cause adverse pregnancy outcomes, and even cause neurological damage in offspring [30]. However, till now, whether exposure to excessive Zn might induce unexplained miscarriage is completely unknown and should be fully investigated.

Disulfidptosis is a newly identified programmed cell death caused by excessive disulfide stress in kidney cancer cells [31], which is different from various classically known cell death forms, such as necroptosis, ferroptosis, or apoptosis. Disulfidptosis is characterized as excessive cystine intake, an increase of NADP^+^/NADPH ratios (i.e., depletion of NADPH, nicotinamide adenine dinucleotide phosphate), the formation of disulfide bonds in actin cytoskeleton proteins, and the collapse of the actin filament (F‐actin) network [31]. As a critical cystine transporter, SLC7A11 (solute carrier family 7 member 11) plays a crucial role in cystine uptake and regulation of disulfidptosis [31, 32]. Till now, it has been reported that disulfidptosis is related only to hepatocellular carcinoma [33], bladder cancer [34], and lung adenocarcinoma [35]. However, whether disulfidptosis might be associated with miscarriage and whether Zn exposure might cause disulfidptosis are completely unknown and should be urgently explored.

Therefore, in this study, we expect to explore the association, causality, and underlying mechanisms among Zn exposure, disulfidptosis, and miscarriage by using two UM case‐control groups, ZnCl_2_‐exposed mouse model, and ZnCl_2_‐exposed trophoblast Swan 71 cell model. Trophoblast cells play crucial roles in healthy pregnancy [36], and Swan 71 cells have been widely used as a cell model in miscarriage studies [7, 8, 9, 10, 11, 12, 13, 14]. We obtain the consistent conclusion that excessive Zn exposure causes disulfidptosis and thus induces miscarriage by up‐regulating the GATA1/METTL1/SLC7A11 axis. This study not only discovers new health risk effects of Zn exposure and novel pathogenesis and biological mechanisms of Zn exposure‐induced unexplained miscarriage but also provides potential targets for treatment against unexplained miscarriage.

## 2 Materials and Methods

### 2.1 Chemicals and Reagents

ZnCl_2_ (99.99% purity) was purchased from Aladdin (7646‐85‐7). ZnCl_2_ was dissolved in ddH_2_O to make a 200 mm ZnCl_2_ stock. Saline was purchased from Macklin.

### 2.2 Cell Culture

Swan 71 cells, first‐trimester human trophoblast cells that were immortalized by human telomerase, were constructed by Gil Mor's group at Yale University [32] and were received as gifts. Swan 71 cells were cultured in DMEM medium (Gibco, C11995500BT) supplemented with 10% FBS (Gibco, 1347575) at 37°C in a humidified atmosphere containing 5% CO_2_. Zn levels in the serum of healthy women at reproductive age were in the range of 8–55 µm [37], which represented the real Zn levels that might contact with the placenta during pregnancy. However, hemolysis might increase Zn levels in plasma because erythrocytes contained approximately 10‐fold higher concentrations of Zn than plasma [38]. Exposure to higher levels of Zn showed cytotoxicity. Compared with physiologically normal Zn levels (20 µm), the elevated Zn levels (40 or 80 µm) suppressed HTR‐8/SVneo cell proliferation, and exposure to 200 µm ZnSO_4_ induced apoptosis in HTR‐8/SVneo cells [38]. Moreover, exposure to 50–100 µm Zn reduced cell viability and increased apoptosis in a dose‐dependent manner in non‐small cell lung cancer cells [39]. Based on the doses used in literature and pre‐experiments, in this study, we selected 0 (control), 50, 100, 200, or 500 µm ZnCl_2_ and treated trophoblast cells for 24 h to construct Zn‐exposed human trophoblast cell models.

### 2.3 Cell Transfection

Human trophoblast Swan 71 cells were transfected with an overexpression plasmid or siRNA to overexpress or knockdown some certain genes, respectively. Empty vector pcDNA3.1 (Catalog No. V790‐20) was purchased from Thermo Fisher Scientific Company. cDNAs that were used for the construction of overexpression plasmid of GATA1 (pcDNA3.1‐GATA1), METTL1 (pcDNA3.1‐METTL1), or SLC7A11 (pcDNA3.1‐SLC7A11) were synthesized and constructed into the pcDNA3.1 vector by Addgene (Watertown, MA, USA). The corresponding RNA sequences were obtained from the National Center for Biotechnology Information (NCBI) database (Gene Bank, Homo sapiens, GRCh38.p14; sequences in Table S1). Empty vector pcDNA3.1 was used as a negative control. Si‐GATA1, si‐METTL1, si‐SLC7A11, and si‐NC (negative control) were customized by Thermo Fisher (sequences in Table S2). Swan 71 cells (1 × 10^6^ cells/well) were seeded in 6‐well plates and cultured to 80% confluence. Trophoblast cells were transfected with 1 µg plasmids or 50 nm siRNAs in turbofect transfection reagent (R0531, Thermo Scientific) for 24 h according to the manufacturer's protocols. The transfection efficiencies were validated by RT‐qPCR.

### 2.4 Cell Viability

To measure cell viability, 5000–10 000 cells per well were seeded in 96‐well plates for 1 day before treatments. Then, the medium in each well was replaced with 100 µL fresh medium containing 10% Cell Counting Kit‐8 (CCK‐8) reagent (ApexBio, #K1018). After incubation for 1 h in a cell incubator, the plate was read by a microplate reader at an absorbance of 450 nm. Cell viability (%) = [(the absorbance of the tested cells—the absorbance of blank)/ (the absorbance of the control cells—the absorbance of blank)] × 100.

### 2.5 Fluorescent Staining of Actin filaments and Cellular Membrane

Immunofluorescence imaging was conducted as the methods described previously [31]. Briefly, trophoblast Swan 71 cells in a chamber slide (Thermo Fisher Scientific, 177402PK) were washed once with PBS and fixed with 3.7% paraformaldehyde in PBS at room temperature for 10 min. For actin filament staining only, the fixed cells were permeabilized in permeabilization buffer (0.5% Triton X‐100 in PBS) at room temperature for 5 min, followed by PBS wash twice. The cells were incubated with 100 nm Acti‐stain 555 phalloidin (Cytoskeleton, PHDH1) in PBS in the dark at room temperature for 30 min. Next, the cells were washed twice and incubated with DAPI in Antifade mounting medium (Thermo Fisher Scientific, R37606). For co‐staining of actin filaments and cellular membrane, the above fixed cells were co‐incubated with Cell Mask green plasma membrane stain dye (Thermo Fisher Scientific, C37608) without permeabilization using SiR‐Actin Kit (Cytoskeleton, CY‐SC001) in the dark at room temperature for 30 min. All fluorescence images were captured using a confocal microscope (LSM 880, Zeiss).

### 2.6 Proteomic Analysis

Two pairs of random UM versus HC women villous tissues (0.1 g), 107 versus 0 mg/kg/day ZnCl_2_‐exposed mouse placental tissues (0.1 g), and 100 versus 0 µm ZnCl_2_‐treated human trophoblast Swan 71 cells (5 × 10^6^ cells) were used for proteomic analysis (Novogene, China). proteomic analysis was performed on an Orbitrap Q Exactive HF‐X mass spectrometer (Thermo Fisher Scientific). Briefly, proteins were extracted, and their concentrations were determined by Bradford protein assay (Bio‐Rad, USA). After proteins were digested with Trypsin Gold (Promega), shot‐gun proteomic analysis was performed using an EASY‐nLC 1200 UHPLC system (Thermo Fisher Scientific) coupled with an Orbitrap Q Exactive HF‐X mass spectrometer (Thermo Fisher Scientific) in the data‐dependent acquisition mode. The differentially expressed proteins (DEPs) with differences > twofold and *p* < 0.05 were generated from read counts using the online bioinformatic platform provided by Novogene (https://magic.novogene.com/customer/main#/loginNew). The DEPs were searched in the UniProt Homo sapiens (homo_sapiens_uniprot_2022_1_27.fasta, 203711 sequences) database and Mus musculus (Mus_musculus_uniprot_2022_1_27.fasta, 86492 sequences) database to determine their genome loci. These DEPs were used for gene ontology (GO) and KEGG analysis to generate GO and KEGG plots, respectively [40, 41].

### 2.7 Quantitative Real‐Time Polymerase Chain Reaction (RT‐qPCR)

Total mRNAs were extracted using TRIzol reagent (ThermoFisher Scientific) according to the manufacturer's instructions. RNA quality and quantity were assessed using a NanoDrop 2000 UV spectrophotometer (Thermo Fischer Scientific, Waltham, USA) (Meyer‐Cifuentes et al. 2020). The RNA purity was high as the A260/280 values were in the range of 1.8–2. Then, 2 µg RNAs were used to generate cDNAs through SuperScript II reverse transcriptase (ThermoFisher Scientific). Real‐time PCR was conducted in triplicate in a 20 µL reaction mixture using SYBR GreenER qPCR SuperMix Universal (ThermoFisher Scientific, #11762100). β‐actin was used as an internal control. The primer sequences were listed in Table S3. The levels of mRNAs were expressed as 2^−ΔΔCt^, where Ct was cycle threshold, Δ*Ct* = testing gene (Ct)—average ACTB (Ct), and ΔΔ*Ct *= sample group Δ(Ct)—average control group Δ(Ct).

### 2.8 RNA Stability Analysis

Swan 71 cells (1 × 10^6^ cells/well) were treated with 0 or 100 µm ZnCl_2_ or transfected with 1 µg overexpression plasmid of METTL1 (with empty vector as control) or 50 nm si‐RNA of METTL1 (with si‐NC as control) for 24 h in a 6‐well plate. Then, the cells were treated with 5 µg/mL actinomycin D (Sigma‐Aldrich) to block mRNA transcription. After 0, 2, 4, 6, 8, or 10 h, RNAs were extracted from cells, and SLC7A11 mRNA levels were analyzed by RT‐qPCR assays. GAPDH mRNA was used as the normalization internal standard.

### 2.9 Western Blot (WB) Analysis

Swan 71 cells, villous tissues, and placental tissues were lysed by RIPA lysis buffer (Thermo Fisher Scientific) and quantified using Pierce BCA Protein Assay Kit (Pierce, Rockford, IL, USA), and were then boiled for 10 min at 95°C. Proteins (10–30 µg/well, equal amounts within group but different amounts among groups for better comparison) were separated on 6%–12% SDS‐PAGE gel and transferred on an equilibrated polyvinylidene difluoride membrane (PVDF, Amersham Biosciences, Buckinghamshire, UK). Subsequently, the membrane was incubated with appropriate primary antibody at 4°C overnight with gentle shaking and then incubated with HRP‐conjugated anti‐rabbit or ‐mouse secondary antibody (Invitrogen, 1:5000, G‐21040/ G‐21234) and Pierce ECL Plus Western Blotting Substrate (ThermoFisher Scientific, 32 132). The primary antibodies included GATA1 (1:1000, ab181544, Abcam), METTL1 (1:1000; 11525‐MM05, SinoBiological), SLC7A11 (1:1000; 12691S, Cell Signaling Technology), GAPDH (1:10 000, ab8245, Abcam), FLNA (1:500; 47 62S, Cell Signaling Technology), MYH9 (1:500; 3403S, Cell Signaling Technology), Drebrin (1:2000; 10260‐1‐AP), ACTB (1:500; MA5‐11869, Thermo Fisher Scientific). The secondary antibodies included goat anti‐rabbit IgG (ab205718, Abcam, dilution 1:10 000) and goat anti‐mouse IgG (ab6789, Abcam, dilution 1:10 000). Afterward, the PVDF membrane was washed thrice, and proteins were detected by enhanced chemiluminescence (Amersham Corporation, Arlington Heights, IL, USA). The intensity of each band was quantified by ImageJ. The value of each band density in experimental and control groups was normalized to that of its corresponding GAPDH band (loading control, ratio to GAPDH%).

### 2.10 ChIP (Chromatin Immunoprecipitation) Assay

ChIP assays were performed using EZ‐Magna ChIP Chromatin Immunoprecipitation Kit (Millipore), as described previously [9]. Briefly, trophoblast cells (2 × 10^7^ cells) or 0.2 g villous tissues or placental tissues were digested using trypsin, cross‐linked in PBS containing 1% formaldehyde at room temperature for 15 min, and then quenched with 125 mm glycine for 5 min. DNAs were extracted and sonicated to generate DNA fragments with 300–600 bp, as validated by 2.5% agarose gel electrophoresis. Subsequently, the resulting mixture was incubated with anti‐GATA1 antibody (1:1000, ab181544, Abcam) overnight at 4°C, with an equal weight of IgG antibody (ab172730, Abcam, dilution 1:200) as a negative control. Protein A/G magnetic beads were then added, and the mixture was incubated at 4°C for 4 h to form bead‐protein‐DNA complex. After elution and de‐crosslinking, the precipitated DNA fragments were extracted by DNA extraction reagent (phenol/chloroform/isoamyl alcohol). The promoter regions of SLC7A11 or METTL1 were amplified by RT‐qPCR with specific primers (sequences in Table S3).

### 2.11 MeRIP (Methylated RNA Immunoprecipitation) Assay

N7‐methyladenosine (m7A RNA methylation) modification on SLC7A11 mRNA was determined by MeRIP‐qPCR assays using BersinBio RIP Immunoprecipitation (RIP) Kit (BersinBio) according to the manufacturer's instructions. RNAs were extracted from 2 × 10^7^ trophoblast cells or 0.2 g of women's villous tissues or mouse placental tissues in lysis buffer containing protease inhibitor and RNAase inhibitor. Then, the RNA samples were treated with DNase I and sonicated to generate RNA fragments in 200–500 nt. Fragmented RNAs were incubated with m7G‐methyladenosine antibody (4141‐13, MBL, dilution 1:200) or IgG as a negative control overnight at 4°C in IP binding buffer; and then the mixtures were incubated with Protein A/G magnetic beads (HY‐K0202, MedChemExpress) for another 4 h at 4°C. Afterward, the beads were washed thrice with IP washing buffer. The m7G‐modified RNAs were eluted and extracted from beads for RT‐qPCR analysis with the specific primers (sequences in Table S3). One tenth of the total RNAs was used as Input.

### 2.12 NADP^+^ and NADPH Measurement

The intracellular levels of NADPH and total NADP (NADPH + NADP^+^) were measured as described previously [31]. Briefly, cells were lysed in 300 µL extraction buffer (20 mm nicotinamide, 20 mm NaHCO_3_, 100 mm Na_2_CO_3_) and the supernatant was collected in two 150‐µL aliquots. For the measurement of total NADP, 20 µL supernatant from one 150 µL aliquot was mixed with 80 µL NADP‐cycling buffer (100 mm Tris‐HCl [pH 8 at 25°C], 0.5 mm thiazolyl blue, 2 mm phenazine ethosulfate, 5 mm ethylenediaminetetraacetic acid [EDTA]) containing 1 U of G6PD enzyme (Sigma‐Aldrich, #G4134). After incubation for 1 min in the dark at 30°C, 20 µL 10 mm fresh glucose 6‐phosphate solution was added to the mixture, and the absorbance at 570 nm was measured with a microplate reader every 30 s for 5 min at 30°C. For NADPH measurement, the remaining 150 µL supernatant was incubated at 60°C for 30 min (to destroy NADP^+^ without affecting NADPH), and the absorbance at 570 nm was measured. The extraction buffer was used as a blank, and the unexposed cells were used as a control. Eventually, the concentration of NADP^+^ was calculated by subtracting [NADPH] from [total NADP]. All experiments were performed in triplicate.

### 2.13 Cellular Cystine Intake Assay

Cystine intake levels were measured using the Cystine uptake assay kit (UP05, Dojindo Laboratories) according to the manufacturer's instructions. Swan 71 cells (1 × 10^4^ cells/well) were incubated in a black 96‐well plate for overnight, washed with DMEM medium (cystine‐free, serum‐free, with 2 mm glutamine) thrice, and then incubated in this DMEM medium for 5 min and in this medium containing 200 µL cystine analogue (selenocystine) for 30 min at 37°C. The fluorescence intensity was measured on a fluorescence microplate reader (Synergy HT, Biotek, U.S.A.) with excitation at 490 nm and emission at 535 nm. Cell suspension without cystine analogue was used as the blank, and the unexposed cells were used as the control. The relative cystine intake levels in cells were calculated by the fluorescence intensity in the tested cells—the blank intensity. The data in all groups were normalized against that in the control group.

### 2.14 Glucose Uptake Assays

The glucose uptake was detected by analyzing 2‐dexoyglucose (2‐DG) uptake using a Glucose Uptake Kit (ab136955, Abcam, MA, USA) according to the manufacturer's instructions. Briefly, Swan 71 cells (1 × 10^4^ cells/well) were treated with 10 mm 2‐DG at 37°C for 20 min. After lysis of cells, the cell lysates were heated at 85°C for 40 min and cool down on ice for 5 min followed by treatment with neutralization buffer. After spin, the supernatant was incubated with the reaction buffer at 37°C for 1 h. The fluorescence was then measured with a microplate reader (Elx808, BioTek, VT, USA). Absorbance was measured using a microplate reader at OD 412 nm, and relative glucose uptake was quantified by interpolation from a standard curve.

### 2.15 Cystine Levels in Villous Tissues

Cystine levels in 0.2 g women villous tissues or 0.2 g mouse placental tissues were detected by HPLC‐MS/MS, as the methods described previously [32]. Tissues were lysed in 500 µL pre‐chilled extraction buffer containing 40% acetonitrile, 40% methanol, and 20% water with 1 mm EDTA and 100 mm formic acid. The addition of EDTA prevented potential oxidation induced by metal ions; whereas formic acid prevented the formation of the highly reactive thiolate anion. The supernatant was collected after centrifugation for 10 min at 4°C. Supernatant (90 µL) was mixed with 10 µL isotopically labeled internal standards (^15^N_2_‐cystine, 10 µg/mL). Cystine was then reacted with benzyl chloroformate to form stable carboxybenzyl adducts by reacting between free amines and thiols. Next, 10 µL triethylamine and 1 µL benzyl chloroformate were added to this mixture and were incubated at 37°C for 10 min. The resulting samples were further analyzed using high‐performance liquid chromatography‐mass spectrometry. The samples were separated in an Agilent ZORBAX 300SB‐C_18_ column (2.1 mm × 100 mm × 3.5 µm) and were eluted with a mobile phase of deionized water supplemented with 0.1% (v/v) formic acid (solvent A) and methanol (solvent B) at a total flow of 0.2 mL/min. The mass spectrometer was equipped with a heated electrospray ionization source (AB SCIEX QTRAP 5500 system, Sciex, USA) for optimized detection of cystine and ^15^N_2_‐cystine (internal standard) in positive ionization mode. The selected mass transitions were m/z 353.1 → 208.1 for cystine and m/z 355.2 → 209.1 for ^15^N_2_‐cystine. The data was analyzed using Analyst 1.6.1 software.

### 2.16 Clinical Sample Collection

In this study, unexplained miscarriage (UM) patients and healthy control (HC) women who had elective miscarriage to terminate their unwanted pregnancies in 25–30 years old with 6–10 weeks gestation were recruited from the Eighth Affiliated Hospital of Sun Yat‐sen University at Shenzhen for Group 1 and from the First Affiliated Hospital of Lanzhou University at Lanzhou for Group 2, as the methods described previously [10, 15]. Any woman with clinically known causes of miscarriage was excluded, such as cervical incompetence, chromosome abnormalities, endocrine or metabolic diseases, virus or bacterial infections, as described previously [10, 15]. The UM group did not have a previous successful pregnancy; and the HC group did not report any previous miscarriages. After exclusion, 50 UM and 50 HC women were enrolled at Shenzhen city (case‐control group 1), and 40 UM and 40 HC women were enrolled at Lanzhou city (case‐control group 2). The characteristics of these HC and UM women were listed in Tables S4 and S5, including baseline characteristics (age, residence, education, body mass index), clinical information (gestational days, RBC, WBC, Hb), and lifestyle (household income, smoking, drinking) in the period of three months before miscarriage operation. All these information was obtained from medical records and questionnaires. Villus tissue samples were collected, dissected from other aborted materials, washed sequentially, and immediately frozen in liquid nitrogen. Villus tissue samples were used for Western blot analysis or for immunohistochemical analysis by paraffin embedding and sectioning. Midstream urine samples were collected on the same day before the miscarriage operation and were used for Zn detection. Peripheral blood samples were collected on the same day of the miscarriage operation and stored in BD Vacutainer SST tubes. Serum samples were isolated within 30 min by centrifugation and were stored in aliquots at −80°C until further use. The protocol was approved by the Medical Ethics Committee of the Eighth Affiliated Hospital of Sun Yat‐sen University at Shenzhen for Group 1 and by the Medical Ethics Committee of the First Affiliated Hospital of Lanzhou University at Lanzhou for Group 2. Written informed consents were signed by all the participants before enrollment.

### 2.17 Inductively Coupled Plasma‐Mass Spectrometry (ICP‐MS)

Serum or urine samples were carried into the plasma axial channel to be evaporated, dissociated, atomized, and ionized at high temperatures (approximately 8000 K). The charged positive ions entered the mass spectrometer through the ion acquisition system, and the elements were separated according to their mass‐to‐charge ratio. A standard curve for Zn levels was made in a certain concentration range, in which the intensity of Zn element is proportional to its concentration. Zn levels were determined using an ICP mass spectrometer (PerkinElmer Inc., model NexION 350X, Massachusetts, USA) based on this standard curve [42].

### 2.18 Immunohistochemistry

Immunohistochemical analysis was performed as described previously [43]. In brief, women villous tissues and mouse placental tissues were collected, fixed, and subjected for embedding and then hematoxylin and eosin staining. Subsequently, the tissue sections were incubated with the primary antibodies, such as cleaved‐caspase 3 (1:500, 9661s, Cell Signaling Technology) or 4‐HNE (1:400, ab46545, Abcam), overnight at 4°C. Staining was performed using the Vectastain Elite ABC kit and DAB peroxidase substrate kit (Vector Laboratories). IHC images were randomly taken at 400× magnification using an Olympus BX43 microscope. The immunoreactive score was calculated according to the equation IRS score = percentage of positive cells × intensity of staining.

### 2.19 Mouse Models

#### 2.19.1 Selection of ZnCl_2_ Dose in ZnCl_2_‐Exposed Mouse Models

To explore the effects of the real environmental exposure dose (REED) of Zn on miscarriage, we calculated the actual daily intake dose of Zn in the human body (Table S6). It was reported that the median Zn level in the urine of male residents in Chongqing, China, was 0.243 mg/L/day (base on Zn element) [22]. This dose could be considered as a representative of human REED under an inadvertent environment. Based on the body surface area conversion factor of mouse/human as 12, 0.243 mg/L/day Zn element exposure in humans corresponded to 6.09 mg/kg/day ZnCl_2_ exposure in mice ($\frac{6.09\;mg/kg/day}{M_{ZnCl2\;(136.3)}}=\frac{0.243\;mg/kg/day}{M_{Zn\;(65.38)}\;\times \;12}$). Besides, the LD50 of oral intake of ZnCl_2_ in mice was estimated as 329 mg/kg [44]. Therefore, 6 mg/kg/day ZnCl_2_ (corresponding to 1/55 of mouse LD50, onefold REED) was chosen to treat pregnant mice. In mouse models, treatment of mice with 28 mg/kg ZnCl_2_ by intraperitoneal injection suppressed the functions and the synthesis of hepatic cytochrome P450 enzymes [45]. Therefore, we selected 33 mg/kg/day ZnCl_2_ (corresponding to 1/10 of LD50, 5.5‐fold REED) as a medium exposure dose in sub‐chronic toxicity assays. It was reported that anemia, neutropenia, and immune suppression occurred when humans were ingested with metallic dietary supplements containing 300 mg/70 kg/day Zn (based on Zn element mass and average body weight of 70 kg) [22]. Zn element exposure at 300 mg/70 kg/day in humans corresponded to 107 mg/kg/day ZnCl_2_ exposure in mice ($\frac{107\;mg/kg/day}{M_{ZnCl2}\;\left(136.3\right)}=\frac{4.3\;mg/kg/day}{M_{Zn}\;\left(65.38\right)}\times 12$). Thus, we selected 107 mg/kg/day ZnCl_2_ (corresponding to 1/3 of LD50, 17.8‐fold REED) as the maximal dose, which corresponded to oral exposure dose with adverse outcomes. Therefore, pregnant mice were exposed with 0, 6, 33, or 107 mg/kg/day ZnCl_2_ to mimic control, the real environmental exposure dose, median dose, or adverse outcome exposure dose, respectively, in this mouse model, corresponding to 0 (control), 1‐, 5.5‐, or 17.8‐fold REED of Zn exposure (Table S6). To eliminate the potential effects of Cl^−^, mice were treated with saline as a control [46].

### 2.20 Four Different Mouse Models

#### 2.20.1 Herein, We Constructed Four Different Mouse Model

Model 1. A ZnCl_2_‐exposed pregnant mouse model was constructed as the similar methods described previously [9, 47]. Pregnant C57BL/6 mice (Charles River Company, Beijing, China) were randomly divided into four groups (*n* = 6 per group). The appearance of a vaginal copulation plug was considered as day 1 (D1) of pregnancy, which was further confirmed by weighting mouse body every day. The pregnant mice were exposed to 0, 6, 33, or 107 mg/kg/day ZnCl_2_ by oral gavage every day from D1 to D13 (*n* = 6): ① control group, treated with the same volume of saline solution, ② 6 mg/kg/day ZnCl_2_‐exposed group (low‐dose group, corresponding to 1/55 of LD50), ③ 33 mg/kg/day ZnCl_2_‐exposed group (medium‐dose group, corresponding to 1/10 of LD50), ④ 107 mg/kg/day ZnCl_2_‐exposed group (high‐dose group, corresponding to 1/3 of LD50).

Model 2. The ZnCl_2_‐exposed pregnant mouse model was treated with AS‐Slc7a11 (antisense oligonucleotides of Slc7a11). The AS‐oligonucleotides specifically bind with their complementary mRNA sequences and promote the degradation of mRNAs [48], which were widely used in various clinical therapy studies [49]. The introduction of locked nucleic acid could further increase the stability and improve the pharmacokinetic properties of AS‐oligonucleotides [50, 51]. Pregnant mice were randomly divided into four groups (*n* = 6): ① control group, treated with the same volume of saline solution, ② 100 mg/kg/day ZnCl_2_, ③ 100 mg/kg/day ZnCl_2_ and AS‐NC, ④ 100 mg/kg/day ZnCl_2_ and AS‐SLC7A11. Pregnant mice were exposed to 0 or 100 mg/kg/day ZnCl_2_ by oral gavage every day from D1 to D13 and were also intraperitoneally injected with 20 mg/kg/3 day AS‐SLC7A11, with AS‐NC as a control, once per three days from D1 to D13.

Model 3. The ZnCl_2_‐exposed pregnant mouse model was also treated with AS‐GATA1 or AS‐METTL1. ZnCl_2_‐exposed pregnant mice were randomly divided into three groups (*n* = 6): ① 100 mg/kg/day ZnCl_2_ and AS‐NC, ② 100 mg/kg/day ZnCl_2_ and AS‐GATA1, ③ 100 mg/kg/day ZnCl_2_ and AS‐METTL1. Pregnant mice were exposed to 100 mg/kg/day ZnCl_2_ every day from D1 to D13 and were also intraperitoneally injected with 20 mg/kg/3 day GATA1 or AS‐METTL1, with AS‐NC as a control, once per three days from D1 to D13.

Model 4. The ZnCl_2_‐exposed pregnant mouse model was also treated with NADPH. ZnCl_2_‐exposed pregnant mice were randomly divided into two groups (*n* = 6): ① 100 mg/kg/day ZnCl_2_ and DMSO; and ② 100 mg/kg/day ZnCl_2_ and NADPH. Pregnant mice were exposed to 100 mg/kg/day ZnCl_2_ every day from D1 to D13 and were also intraperitoneally injected with 5 mg/kg/day NADPH, with DMSO as solvent and control, once per day from D1 to D13.

On D14, all mice were euthanized by injection with nembutal (100 mg/kg) for the collection of the uterus. The embryo resorption was identified by a smaller or darker appearance relative to the viable and pink healthy embryos [52]. The miscarriage rate in each mouse and the average miscarriage rate in each group were calculated by (the number of adsorbed embryos)/(the total number of normal embryos and the adsorbed embryos) [52]. RNAs and proteins were extracted from a random placenta in each mouse for RT‐qPCR and Western blot analysis, respectively. The gene conservation and homology analysis were conducted using the UCSC Blast‐like alignment tool (BLAT) genome browser (https://genome.ucsc.edu/cgi‐bin/hgBlat). The protein conservation and homology analysis were conducted using Uniprot (https://www.uniprot.org/). The animal project was authorized by the Ethics Committee of the Eighth Affiliated Hospital of Sun Yat‐sen University.

### 2.21 Structural and Energy Analysis of ZnCl2‐Exposed GATA1 and ZnCl2‐Exposed GATA1 Bound With SLC7A11 or METTL1 Promoter Region

Selection of GATA1 region and SLC7A11 or METTL1 promoter region: Human protein structure GATA1_HUMAN (UniProt ID: P15976) was selected from the UniProt database. The DNA‐binding domain of GATA1 contained amino acid residues from 200 to 318. JASPAR database showed the highest‐scoring DNA fragment to bind with GATA1: SLC7A11 promoter region 5’‐TCCTTATCTCA‐3’ and METTL1 promoter region 5’‐TTATAATCTAA‐3’.

Molecular dynamics (MD) simulations: The structure of the GATA1 protein was constructed by Alphafold3. The 3D structure was visualized by PyMOL v2.5.4 and its 2D structures were analyzed using PDBsum. The structures of GATA1 bound to SLC7A11 or METTL1 promoter region in the presence of 200 µm ZnCl_2_ (with 200 µm NaCl as a control group) were analyzed by MD simulations (within 100 ns) using GROMACS 2020.6. Protein parameters and topology files were generated using the Amber03 force field. In the simulation box, periodic boundary conditions were applied, and GATA1 protein or GATA1‐DNA complexes were centered in a cubic box (minimum 1 nm edge distance) with TIP3P water as solvent. Charge neutrality was maintained by replacing the solvent with 200 µm NaCl or ZnCl_2_. Energy minimization was conducted via the steepest descent algorithm to eliminate steric clashes and to optimize solvent orientation.

Production MD simulations were executed under isothermal‐isobaric conditions (303.15 K, 1 bar) within 100 ns using the leapfrog algorithm. For post‐simulation, trajectories were aligned to the protein backbone; and root mean square deviation (RMSD) was analyzed.

Ramachandran plot assessment: The Ramachandran plot (generated using the PyMod module in PyMOL v2.5.4) evaluated the quality of the 3D structural model of the GATA1 protein. This model exhibited strong overall quality with 82.4% residues in the most favored regions (red), 15.7% in additional allowed regions (yellow), 2% in generously allowed regions (light yellow), and 0.0% in disallowed regions (white).

3D structural analysis and visualization: The 3D structures of GATA1 or GATA1‐DNA complex were constructed by AlphaFold3; and the detailed 3D structures and binding interface analysis were visualized by PyMOL v2.5.4. The 3D structure of the GATA1 protein contained α‐helices in red, β‐sheets in yellow, and random coils in green. The 3D structure of GATA1‐DNA complex contained stable interactions, such as H‐bonds (formed between polar donors (e.g., N─H) and acceptors (e.g., O, N), energy range in 10–40 kJ/mol), salt bridges (formed between oppositely charged residues, distance ≤ 5.5 Å, energy range in 3–13 kJ/mol), hydrophobic interactions (aggregation of hydrophobic groups), π–π Stacking (weak interactions of aromatic rings between electron‐rich and electron‐deficient systems), π‐cation interactions (interactions between π‐systems (e.g., aromatic rings) and cations (e.g., positively charged residues)).

Binding energy calculation: The binding energies (kcal/mol) of GATA1‐SLC7A11 or ‐METTL1 promoter region were analyzed by gmx_MMPBSA v1.6.1 (single trajectory method).

### 2.22 Statistical Analysis

All experiments were replicated thrice independently with similar results. The measured or calculated data (including the control group) were presented as mean ± SD (standard deviation, *n* = 3). SPSS26.0 or R software was used to analyze the data, and GraphPad Prism 8.0 as well as R software was used for figure generation. The data about the association among molecules and diseases were obtained from the Comparative Toxicogenomics Database (CTD; http://ctdbase.org). The alluvial plot was visualized using the online OmicShare tools (https://www.omicshare.com/tools). In cellular assays, the number *n* = 3 indicated that all experiments were replicated thrice independently. In the animal model, the number *n* = 6 indicated 6 mice in each group. In villous tissue assays, *n* = 50 indicated 50 pairs of HC and UM tissue or serum samples. Levene's test was used to evaluate the homogeneity of variance of the data. Student's *t*‐test was used to compare differences between two groups. ANOVA and Fisher's least significant difference (LSD) test were used to compare differences among multiple groups. The correlation analysis of the relative expression levels was performed using Pearson analysis. When *p* < 0.05, the difference was considered to be statistically significant.

## 3 Results

### 3.1 Section I High Levels of Zn Exposure Induced Miscarriage

#### 3.1.1 The Levels of Internal Zn in Serum or Urine Were Associated with Unexplained Miscarriage

**FIGURE 1:**
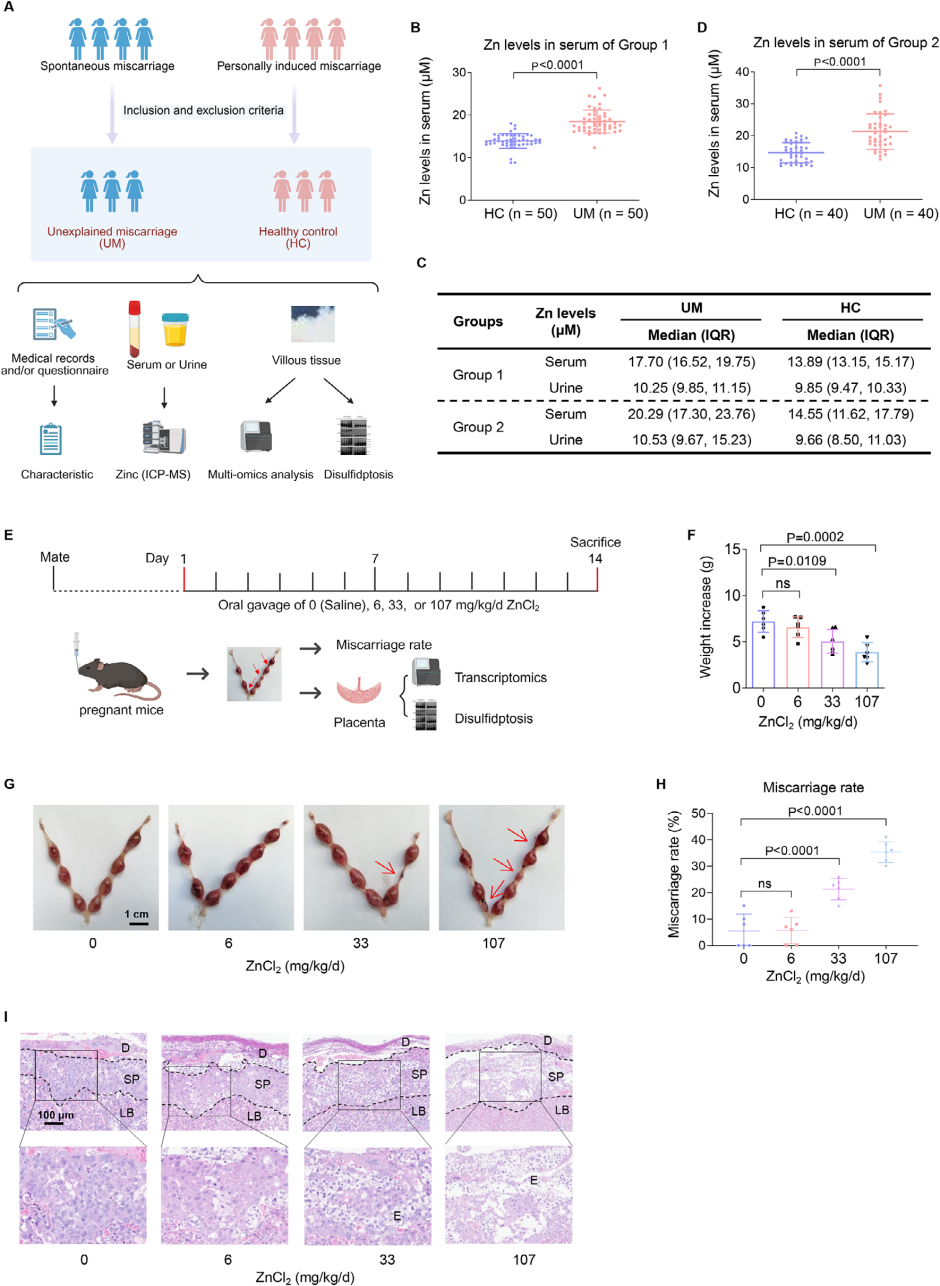
High levels of Zn exposure induced miscarriage. (A) Schematic diagram of UM case‐control studies. (B) The levels of Zn (µm) in HC and UM women's serum samples of case‐control Group 1 (*n* = 50). (C) Distribution of Zn concentrations (µm) in HC and UM women's serum and urine samples of Group 1 (*n* = 50) and Group 2 (*n* = 40). IQR, interquartile range. (D) The levels of Zn (µm) in HC and UM women's serum samples of case‐control Group 2 (*n* = 40). (E) Schematic diagram of ZnCl_2_‐exposed mouse model. Pregnant mice were treated with 0, 6, 33, or 107 mg/kg/day ZnCl_2_ by oral gavage for a continuous 13 days (each *n* = 6). (F) The increase in body weight of ZnCl_2_‐exposed pregnant mice (the weight on D14 – the initial weight on D1) (each *n* = 6). (G,H) Representative images of mouse embryo resorption (indicated by red arrows) (G) and the average miscarriage rates (H) of ZnCl_2_‐exposed mice (each *n* = 6). (I) The HE staining of the placenta of ZnCl_2_‐exposed pregnant mice on Day 14. The decidua (D), spongiotrophoblast (SP), labyrinth (LB), and edema (E) were labeled (scale bar, 100 µm).

To explore the potential correlation between Zn exposure and unexplained miscarriage (UM), we newly collected serum and urine samples from UM patients and their matched healthy control (HC) group (*n* = 50) from Shenzhen city (case‐control Group 1, southeast China) (Figure 1A). The known causes, such as autoimmune abnormality, uterine abnormalities or cervical incompetence, the symptoms of endocrine or metabolic diseases, luteal phase defects, and antiphospholipid antibody syndrome, have been excluded, as the similar methods described previously [10, 15]. To reduce the effects of potential confounders, variables were collected based on prior studies and their potential associations with miscarriage [53, 54], such as baseline characteristics, clinical information, and lifestyle (Table S4). These parameters did not show statistically significant differences (Table S4). The Zn levels in serum and urine samples were detected by ICP‐MS. The median of Zn levels was 17.70 µm in the UM group and 13.89 µm in the HC group in serum samples (Figure 1B,C), and 10.25 µm in the UM group and 9.85 µm in the HC group in urine samples (Figure S1A,C). Zn levels in serum or urine samples were statistically significantly higher in UM versus HC groups (Figure 1B; Figure S1A). In the unadjusted model, the univariate logistic regression model analysis showed that higher Zn levels in serum (*OR* = 4.235, 95% CI, 2.378–7.543) or urine (*OR *= 1.769, 95% CI, 1.129–2.771) samples were associated with miscarriage (Figure S1B, Table S6). Based on directed acyclic graph analysis and previous literature [53, 54], the variables such as age, BMI, education, household income, smoking, and drinking were considered as potential confounders (Figure S1C). To reduce their effects, multivariate logistic regression model analysis by adjusting for all these confounders showed that Zn levels were associated with miscarriage (the adjusted OR of 4.707 with 95% CI of 2.394–9.256 in serum; and the adjusted OR of 1.907 with 95% CI of 1.128–3.224 in urine, Figure S1B, Table S7). To further examine whether the estimated association differed among subpopulations, stratification analysis showed that the stratifying factors did not significantly alter the association between Zn levels and miscarriage (all *P* for interaction > 0.05, Figure S1D,E), confirming the robustness of these results. Collectively, all the statistical analyses confirmed that Zn exposure was positively associated with unexplained miscarriage.

To further explore this, we also recruited another case‐control group from Lanzhou city (northwest China, 2350 km far away from Shenzhen city, Group 2) and collected HC and UM serum and urine samples (*n* = 40), based on the same methods as Group 1. The known causes have been excluded. Their parameters did not show statistically significant differences (Table S5). Zn levels in serum or urine samples were statistically significantly higher in UM versus HC groups (Figure 1C,D; Figure S1F). Univariate logistic regression model analysis showed that higher levels of Zn in serum (*OR* = 1.486, 95% CI, 1.241–1.778) or urine (*OR* = 1.446, 95% CI, 1.147–1.824) were associated with unexplained miscarriage (Figure S1B). After adjusting for these confounders, multivariate logistic regression analysis showed that the adjusted OR values were 1.792 with 95% CI of 1.363–2.356 in serum (Figure S1B, Table S7) and 1.490 with 95% CI of 1.155–1.922 in urine (Figure S1B, Table S7) in group 2, showing that higher Zn levels were positively associated with unexplained miscarriage. To further examine whether the estimated association differed among subpopulations, the stratification analysis showed that the stratifying factors did not significantly alter the association between Zn levels and miscarriage (all P for interaction > 0.05, Figure S1G,H), confirming the robustness of these results. Taken together, both case‐control groups showed that higher levels of Zn in serum or urine samples were positively associated with unexplained miscarriage.

#### 3.1.2 Exposure to Higher Levels of Zn Induced Unexplained Miscarriage

To directly explore the causality whether Zn exposure during pregnancy might induce miscarriage, we constructed a ZnCl_2_‐exposed mouse model by treating pregnant mice with 0, 6, 33, or 107 mg/kg/day ZnCl_2_ to mimic control, the real environmental exposure dose, median dose, or adverse outcome exposure dose, respectively, as described in the Method section (Figure 1E). Treatment with 107 mg/kg/day ZnCl_2_ (17.8‐fold REED) did not result in acute mortality or overt signs of severe poisoning, and did also not obviously affect mouse hair/behavioral phenotypes (Figure S1I), showing that this 17.8‐fold REED of ZnCl_2_ might not cause detectable systemic toxicity in this model. Notably, ZnCl_2_ exposure elevated Zn content in mouse placental tissues (Figure S1J), reduced the mouse body weight gain (Figure 1F; Figure S1K), increased embryo absorption (Figure 1G), and elevated average miscarriage rates (Figure 1H), indicating that exposure to higher levels of Zn induced mouse miscarriage. In addition, HE staining also showed that placental tissues became loose and produced edema after Zn exposure (Figure 1I). Collectively, these results confirmed that exposure to higher levels of Zn caused mouse miscarriage.

### 3.2 Section II Zn Exposure Induced Miscarriage by Promoting Disulfidptosis

#### 3.2.1 Disulfidptosis Might be Involved in Zn Exposure‐Induced Miscarriage

To discover which kind of phenotype might contribute to this Zn exposure‐induced miscarriage, two pairs of random HC and UM villous tissues from the case‐control Group 1 were used for proteomic analysis, giving 754 up‐regulated proteins and 396 down‐regulated proteins with a difference > twofold and *p* < 0.05 (Figure S2A). Placental tissues from two random pairs of 107 versus 0 mg/kg/day ZnCl_2_‐exposed mice were used for proteomic analysis, giving 399 up‐regulated proteins and 539 down‐regulated proteins with a difference > twofold and *p* < 0.05 (Figure S2B). Trophoblast cells play important roles in healthy pregnancy, and trophoblast Swan 71 cells have been widely used as a cell model in miscarriage studies [9]. To correlate Zn exposure, trophoblast cell dysfunctions, and miscarriage, we also treated Swan 71 cells with 100 versus 0 µm ZnCl_2_ and used them for proteomic analysis, giving 140 up‐regulated and 134 down‐regulated proteins with differences > twofold and *p*‐values < 0.05 in ZnCl_2_‐exposed Swan 71 cells (Figure S2C). In the intersection of three proteomic data, there were 5 up‐regulated and 3 down‐regulated proteins (Figure S2D). GO and KEGG (Figure S2E–J) analysis of these differentially expressed proteins (DEPs) in UM versus HC villous tissues, 107 versus 0 mg/kg/day ZnCl_2_‐exposed mouse placental tissues, and 100 versus 0 µm ZnCl_2_‐exposed Swan 71 cells showed that cell adhesion, cell death, mitochondrial membrane, metabolic pathways, and actin cytoskeleton were top significantly regulated, implying that disulfidptosis might be top significantly altered. PPI (Protein–Protein Interaction Networks) analysis showed that FLNA, MYH9, Drebrin, and F‐actin were associated and significantly differentially expressed (Figure S2K). Collectively, these sequencing data implied that Zn exposure, trophoblast cell or villous tissue disulfidptosis, and miscarriage were positively associated.

#### 3.2.2 Zn Exposure Caused Trophoblast Cell Disulfidptosis

To experimentally validate this, in trophoblast cells, we found that Zn exposure suppressed trophoblast cell viability (Figure S2L). Moreover, the cell viability could be well recovered by treatment with disulfide stress inhibitor tris‐(2‐carboxyethyl)‐phosphine (TCEP), necroptosis inhibitor Necrostatin‐1 (Nec‐1), or apoptosis inhibitor Z‐VAD‐fmk, but not or less by ferroptosis inhibitor Ferrostatin‐1 (Ferr‐1) or pyroptosis inhibitor Ac‐YVAD‐cmk (Figure 2B). In this study, we focused on disulfidptosis and explored how Zn exposure caused disulfidptosis to induce miscarriage. To identify this, we further detected the biomarkers of disulfidptosis. Zn exposure increased the levels of cystine intake and NADP^+^/NADPH ratios (Figure 2C,D). Non‐reducing WB analysis also showed that Zn exposure promoted the formation of disulfide bonds (i.e., Cys‐crosslink) in FLNA, MYH9, Drebrin, and F‐actin (Figure 2E,F). Confocal assays further confirmed that Zn exposure promoted F‐actin contraction and F‐actin detachment from the plasma membrane (F‐actin in red and membrane in green, Figure 2G; Figure S2M,N). Collectively, all these results confirmed that Zn exposure could cause trophoblast cell disulfidptosis.

FIGURE 2Zn exposure induced miscarriage by promoting disulfidptosis. (A) Schematic diagram of cellular studies using ZnCl_2_‐exposed human trophoblast Swan 71 cells. (B) CCK8 assay analysis of cell viability of 100 µm ZnCl_2_‐exposed Swan 71 cells with treatment with Nec‐1, Z‐VAD‐fmk, TCEP, Fer‐1, or Ac‐YVAD‐cmk for 0, 12, 24, 36, or 48 h. (C) Cystine levels in 0, 50, 100, 200, or 500 µm ZnCl_2_‐exposed Swan 71 cells. (D) The NADP^+^/NADPH ratios in 0, 50, 100, 200, or 500 µm ZnCl_2_‐exposed Swan 71 cells. (E,F) Western blot analysis of Cys‐crosslinked (non‐reducing) or monomeric (reduced) FLNA, MYH9, Drebrin, and F‐actin protein levels in 0, 50, 100, 200, or 500 µm ZnCl_2_‐exposed Swan 71 cells and the relative quantification of Cys‐crosslinked proteins (*n* = 6). (G) Fluorescence intensity of F‐actin (red, labeled with Acti‐stain 555 phalloidin) in 0 or 200 µm ZnCl_2_‐exposed Swan 71 cells. Scale bar, 30 µm. (H) Fluorescence intensity of Swan 71 cells with overexpression of pEGFP‐F‐actin‐wt or pEGFP‐F‐actin‐mut (mut: Cys‐17 to Ala). Scale bar, 30 µm. (I) Time‐lapse image analysis of F‐actin bodies in Swan 71 cells. Scale bar, 30 µm. (J,K) FRAP assay analysis of fluorescence intensity recovery and quantification of F‐actin bodies in Swan 71 cells after photobleaching. Time 0 (s) indicated the photobleaching pulse. Scale bar, 30 µm. (L) Fluorescence intensity of F‐actin in 0 or 100 µm ZnCl_2_‐exposed Swan 71 cells with treatment with 50 µm TPEN or 1 mm TCEP and with overexpression of pEGFP‐F‐actin‐wt. Scale bar, 30 µm.

**Figure:**
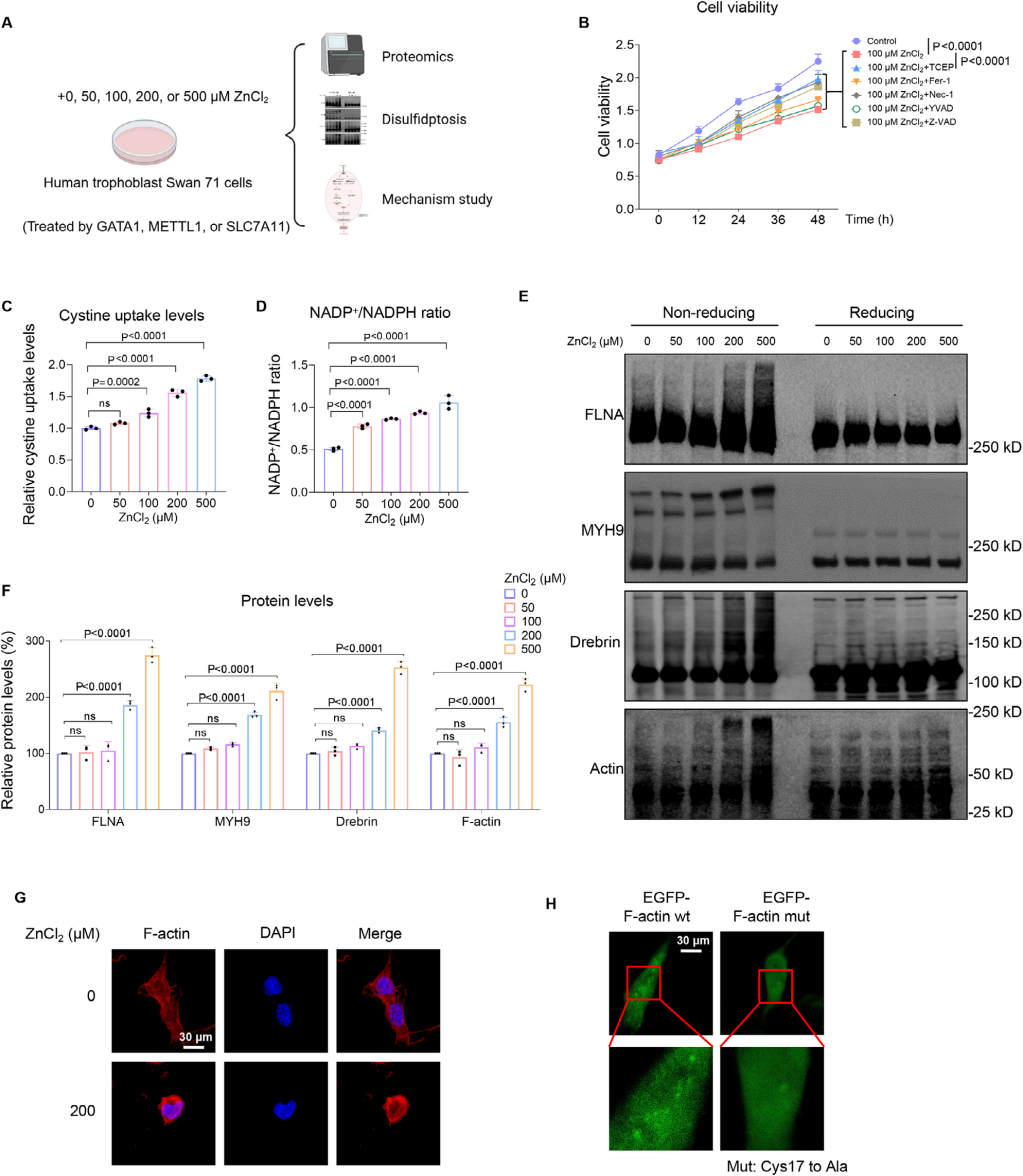

**Figure:**
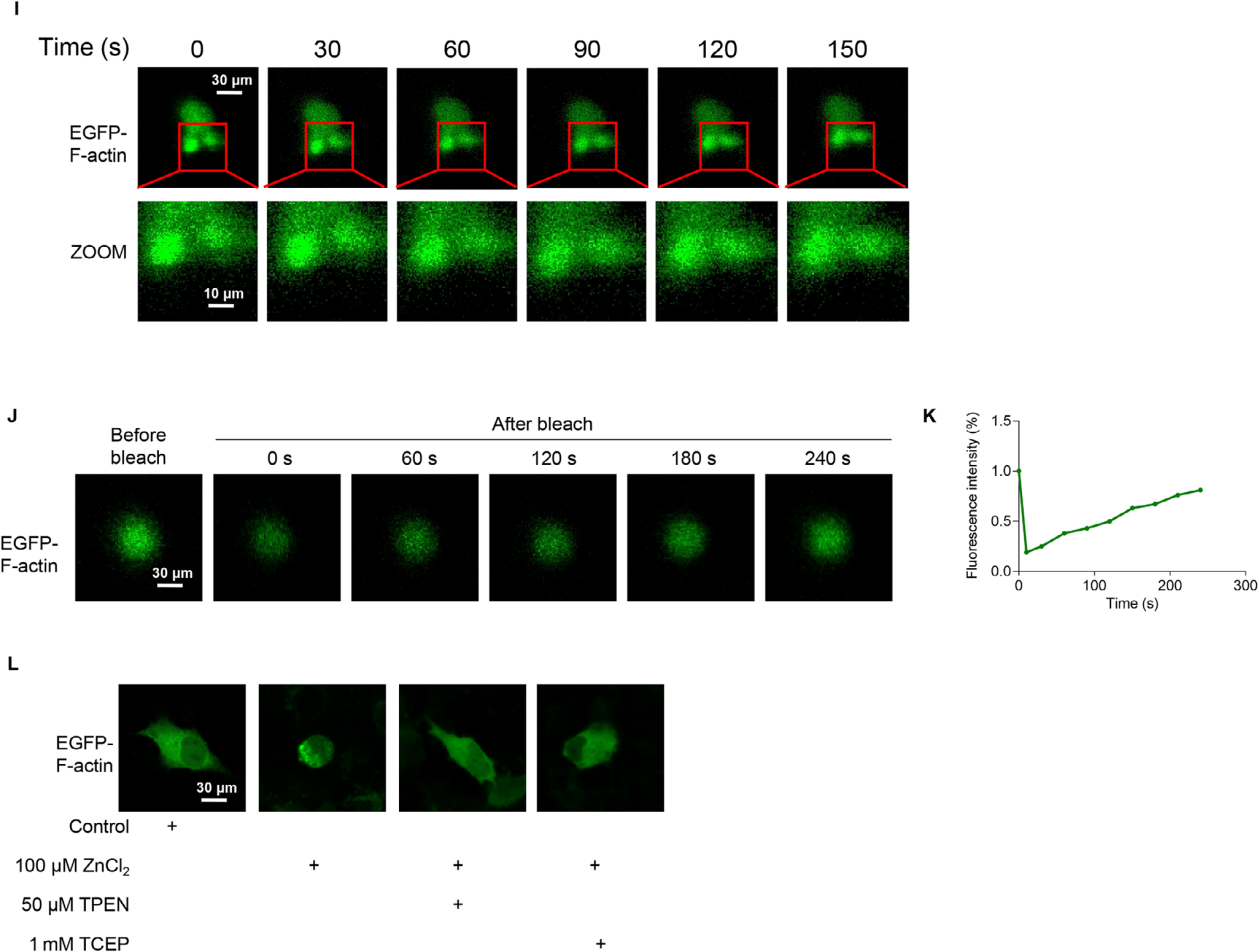

It has been reported that formation of disulfide bonds causes protein disorder [55], a significant feature that drives liquid‐liquid phase separation (LLPS) [56]. Predicted by PONDR (http://www.pondr.com/) and FoldUnfold (http://bioinfo.protres.ru/ogu/), F‐actin might undergo LLPS through its intrinsically disordered regions (IDRs). Con‐focal fluorescent microscopy 3D image revealed that GFP‐labeled F‐actin, but not its mutant (deletion of the disordered regions containing 1–6, 43–56, 98–114, 228–249, and 302–317 residues), could form bodies (green puncta) in cells (Figure S2O). It has been identified by mass spectrometry that the C17 site in F‐actin is responsible for the disulfide bond formation [31]. Then, we also constructed the mutant F‐actin by replacing Cys‐17 to Ala (F‐actin‐mut). It was found that more F‐actin bodies were formed in trophoblast cells with overexpression of F‐action‐wt but less were formed in cells with overexpression of F‐action‐mut (Figure 2H). To further validate whether these bodies were formed through LLPS, we found that these bodies could be fused together with time within living trophoblast cells (Figure 2I). Furthermore, fluorescence recovery after photobleaching (FRAP) assays revealed that the fluorescent signal of these bodies could be recovered after photobleaching (Figure 2J,K). Furthermore, Zn exposure promoted the formation of F‐actin bodies (Figure 2L; Figure S2P); and co‐treatment with TPEN or TCEP suppressed the body formation (Figure 2L). Taken together, these results supported that Zn exposure promoted the formation of F‐actin bodies through LLPS, which was involved in Zn exposure‐induced trophoblast cell disulfidptosis.

#### 3.2.3 Zn Exposure, Disulfidptosis, and Unexplained Miscarriage Were Closely Associated in UM Case‐Control Study

**FIGURE 3:**
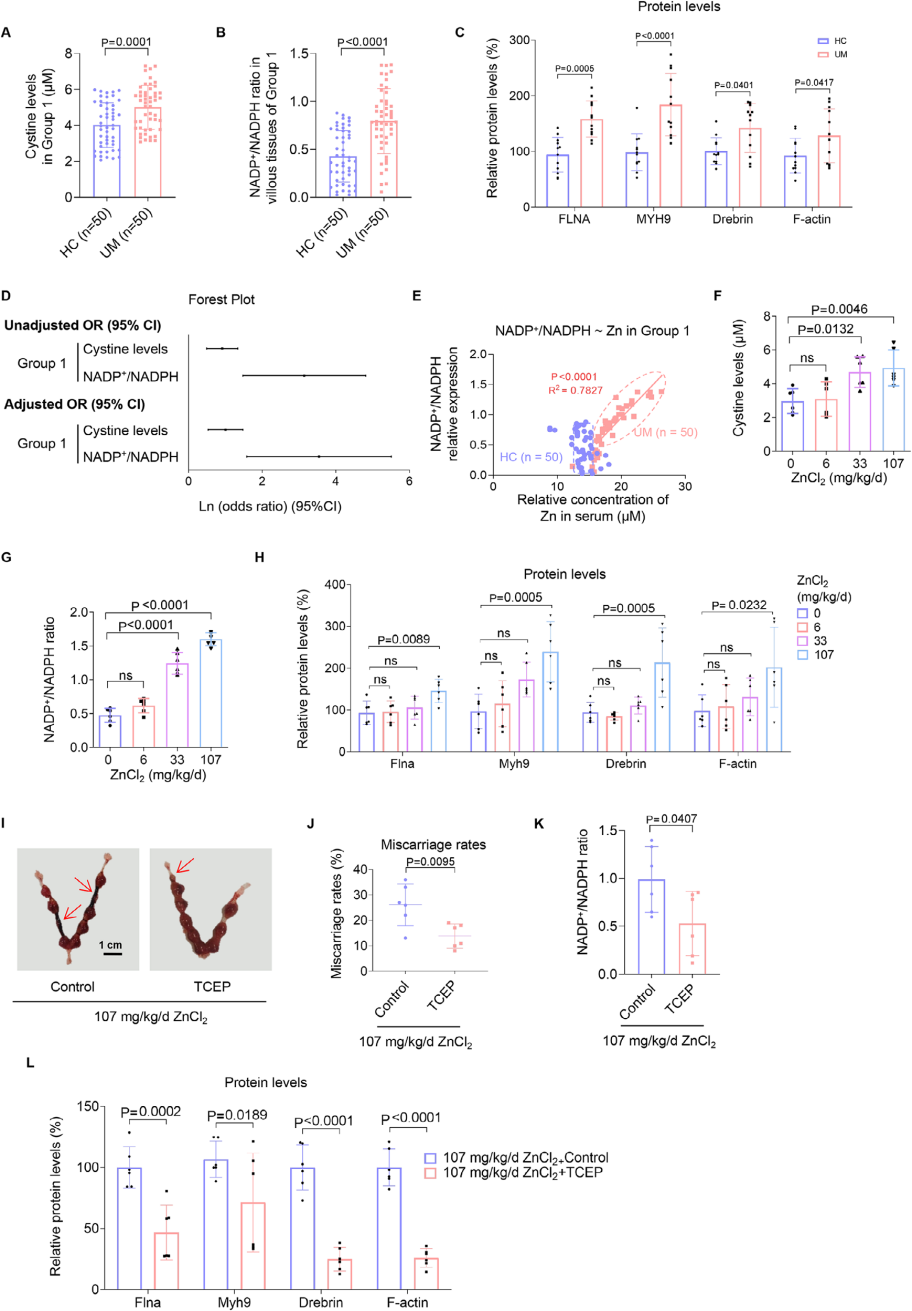
Validation of disulfidptosis in human villous tissues and in the mouse placenta. (A) Cystine levels in villous tissues of Group 1 (*n* = 50). (B) The NADP^+^/NADPH ratio in villous tissues of Group 1 (*n* = 50). (C) Western blot analysis of the protein levels of Cys‐crosslinked (non‐reducing) FLNA, MYH9, Drebrin, and F‐actin in villous tissues of Group 1 (*n* = 12). (D) Univariate and multivariate logistic regression analysis of the association of cystine levels and NADP^+^/NADPH ratio in villous tissues of Group 1 (*n *= 50) with miscarriage. (E) Pearson correlation analysis of the levels of NADP^+^/NADPH ratios with serum Zn levels in HC and UM villous tissues of Group 1 (each *n* = 50). (F) The cystine levels in 0, 6, 33, or 107 mg/kg/day ZnCl_2_‐exposed mouse placental tissues (each *n* = 6). (G) NADP^+^/NADPH ratios in 0, 6, 33, or 107 mg/kg/day ZnCl_2_‐exposed mouse placental tissues (each *n* = 6). (H) Western blot analysis of the protein levels of Cys‐crosslinked (non‐reducing) murine Flna, Myh9, Drebrin, and F‐actin in 0, 6, 33, or 107 mg/kg/day ZnCl_2_‐exposed mouse placental tissues (each *n* = 6). (I) Representative images of mouse embryo resorption (indicated by red arrows) in 107 mg/kg/day ZnCl_2_‐exposed mice with TCEP treatment. (J) The average miscarriage rates of 107 mg/kg/day ZnCl_2_‐exposed mice with TCEP treatment (each *n* = 6). (K) NADP^+^/NADPH ratios in 107 mg/kg/day ZnCl_2_‐exposed mice with TCEP treatment (each *n* = 6). (L) Western blot analysis of the protein levels of Cys‐crosslinked (non‐reducing) murine Flna, Myh9, Drebrin, and F‐actin in 107 mg/kg/day ZnCl_2_‐exposed mouse placental tissues with TCEP treatment (each *n* = 6).

To experimentally validate the levels of disulfidptosis in HC and UM villous tissues, we found that the levels of cystine intake and NADP^+^/NADPH ratios were higher in UM versus HC villous tissues in both case‐control Groups 1 and 2 (Figure 3A,B; Figure S3A,B). Non‐reducing WB analysis also showed that more disulfide bonds were formed in FLNA, MYH9, Drebrin, and F‐actin proteins in UM versus HC villous tissues in both groups (Figure 3C; Figure S3C–E). These results confirmed that the levels of disulfidptosis were higher in UM versus HC villous tissues. Univariate and multivariate logistic regression model analysis showed that higher levels of cystine intake and NADP^+^/NADPH ratios were associated with unexplained miscarriage in both groups (Figure 3D; Figure S3F, Table S7). Collectively, both case‐control studies showed that the levels of disulfidptosis were higher in UM versus HC villous tissues, and higher levels of disulfidptosis in villous tissues were associated with unexplained miscarriage.

Since Zn levels were higher in UM versus HC serum or urine samples, we further analyzed the correlation between Zn levels and disulfidptosis levels (as indicated by higher levels of cystine intake, NADP^+^/NADPH ratios, and disulfide‐bond formation in FLNA, MYH9, Drebrin, F‐actin proteins). Pearson correlation analysis showed that their levels were all correlated in the UM group in both case‐control groups (Figure 3E; Figure S3G–K), indicating the higher levels of Zn in serum or urine samples were positively associated with higher levels of disulfidptosis in UM villous tissues. Taken together, combined with cellular assays, these results implied that Zn exposure, disulfidptosis, and unexplained miscarriage were positively and closely associated.

#### 3.2.4 Zn Exposure Induced Mouse Miscarriage by Promoting Mouse Placental Disulfidptosis in ZnCl_2_‐Exposed Mouse Model

We further validated disulfidptosis in the ZnCl_2_‐exposed mouse miscarriage model. The mRNA and amino acid sequences of Flna, Myh9, Drebrin, and F‐actin were all conserved in rhesus, mouse, dog, and elephant (Figure S3L, Table S8). Zn exposure increased the levels of cystine intake and NADP^+^/NADPH ratios in mouse placental tissues (Figure 3F,G) and promoted the formation of disulfide bonds in Flna, Myh9, Drebrin, and F‐actin proteins (Figure 3H; Figure S3M) in mouse placental tissues, indicating that Zn exposure caused mouse placental disulfidptosis and induced miscarriage.

To further explore whether Zn exposure induced miscarriage through placental disulfidptosis, we constructed a miscarriage intervention model by intraperitoneally injecting TCEP (a reducing agent to prevent disulfide stress) in ZnCl_2_‐exposed mice to study the effects of placental disulfidptosis on mouse miscarriage. TCEP treatment reduced embryo resorption and miscarriage rates in Zn‐exposed mice (Figure 3I,J). Meanwhile, this treatment also efficiently reduced NADP^+^/NADPH ratios in mouse placental tissues (Figure 3K) and suppressed the formation of disulfide bonds in Flna, Myh9, Drebrin, and F‐actin proteins (Figure 3L; Figure S3N) in mouse placental tissues, indicating that the reduced disulfidptosis levels in mouse placental tissues could efficiently alleviate mouse miscarriage. Taken together, these results showed that Zn exposure induced mouse miscarriage by promoting mouse placental disulfidptosis.

### 3.3 Section III Zn Exposure Caused Disulfidptosis by up‐Regulating SLC7A11

#### 3.3.1 Zn Exposure Caused Trophoblast Cell Disulfidptosis by up‐Regulating SLC7A11

To explore which molecule might be involved in Zn exposure‐caused disulfidptosis, in the intersection of proteomic data of UM versus HC villous tissues, 107 versus 0 mg/kg/day ZnCl_2_‐exposed mouse placental tissues, and ZnCl_2_‐exposed trophoblast cells, SLC7A11 was identified as one of the top highly expressed proteins (Figure S2D). SLC7A11 is a cystine transporter that leads to intracellular disulfide stress and disulfidptosis [31]. RT‐qPCR and WB assays confirmed that Zn exposure up‐regulated SLC7A11 mRNA and protein levels in human trophoblast cells (Figure 4A,B; Figure S4A). Overexpression of SLC7A11 decreased cell viability, increased the levels of cystine intake and NADP^+^/NADPH ratios (Figure 4C–E; Figure S4B,C), and promoted the formation of disulfide bonds in FLNA, MYH9, Drebrin, and F‐actin proteins (Figure 4F; Figure S4D), all of which led to disulfidptosis in trophoblast cells. In ZnCl_2_‐exposed trophoblast cells, co‐knockdown of SLC7A11 could restore (i.e., increase) cell viability, decrease the levels of cystine intake and NADP^+^/NADPH ratios (Figure 4G,H; Figure S4E–G), and suppressed the formation of disulfide bonds in FLNA, MYH9, Drebrin, and F‐actin proteins (Figure 4I; Figure S4H), and thus restore (i.e., reduce) disulfidptosis caused by Zn exposure. Therefore, these results showed that Zn exposure up‐regulated SLC7A11 expression levels and thus caused trophoblast cell disulfidptosis.

FIGURE 4Zn exposure caused disulfidptosis by up‐regulating SLC7A11. (A,B) Western blot analysis of SLC7A11 protein levels in 0, 50, 100, 200, or 500 µm ZnCl_2_‐exposed Swan 71 cells and its relative quantification. (C) CCK8 assay analysis of cell viability of Swan 71 cells with SLC7A11 overexpression within 48 h. (D) Cystine levels in Swan 71 cells with SLC7A11 overexpression. (E) The NADP^+^/NADPH ratios in Swan 71 cells with SLC7A11 overexpression. (F) The protein levels of Cys‐crosslinked FLNA, MYH9, Drebrin, and F‐actin in Swan 71 cells with SLC7A11 overexpression. (G) CCK8 assay analysis of cell viability of 100 µm ZnCl_2_‐exposed Swan 71 cells with SLC7A11 knockdown within 48 h. (H) The NADP^+^/NADPH ratios in 100 µm ZnCl_2_‐exposed Swan 71 cells with SLC7A11 knockdown. (I) The protein levels of Cys‐crosslinked FLNA, MYH9, Drebrin, and F‐actin in 100 µm ZnCl_2_‐exposed Swan 71 cells with SLC7A11 knockdown. (J,K) SLC7A11 protein levels in HC and UM villous tissues and its relative quantification (*n* = 12). (L) Univariate and multivariate logistic regression analysis of the association of SLC7A11 mRNA in villous tissues with miscarriage in Groups 1 and 2. (M,N) Western blot analysis of Slc7a11 protein levels in 0, 6, 33, or 107 mg/kg/day ZnCl_2_‐exposed mouse placental tissues (each *n* = 6) and its relative quantification. (O) Western blot analysis of Slc7a11 protein levels in mouse trophoblast cells with AS‐Slc7a11 transfection and its relative quantification. (P,Q) Embryo resorption (indicated by red arrows, P) and the average miscarriage rates (Q) in 107 mg/kg/day ZnCl_2_‐exposed mice with overexpression of murine Slc7a11 (each *n* = 6). (R,S) Western blot analysis of Slc7a11 protein levels in placental tissues of 107 mg/kg/day ZnCl_2_‐exposed mice with Slc7a11 knockdown and its relative quantification (*n* = 6). (T) The protein levels of Cys‐crosslinked Flna, Myh9, Drebrin, and F‐actin in placental tissues of 107 mg/kg/day ZnCl_2_‐exposed mouse with Slc7a11 overexpression (*n* = 6).

**Figure:**
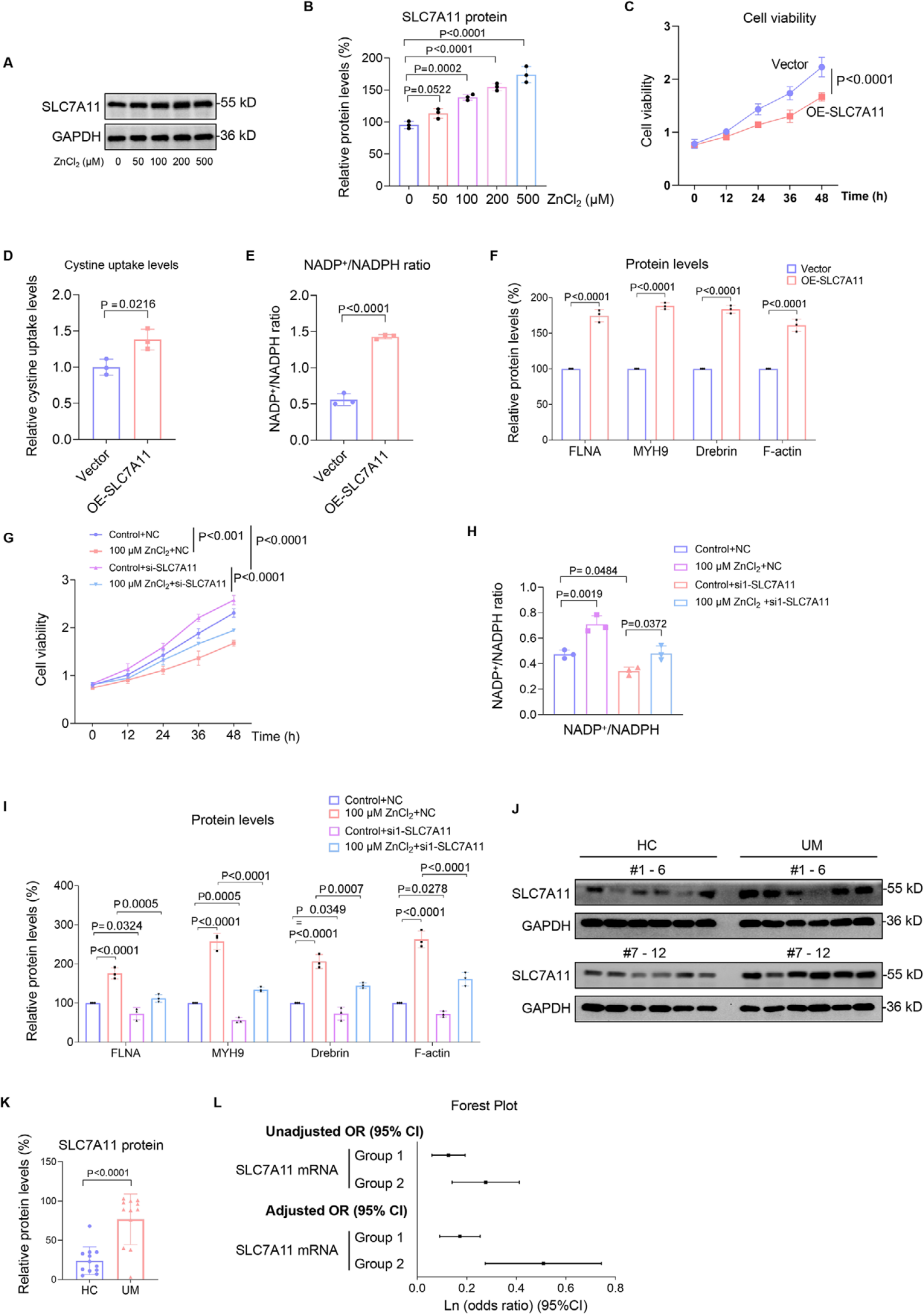

**Figure:**
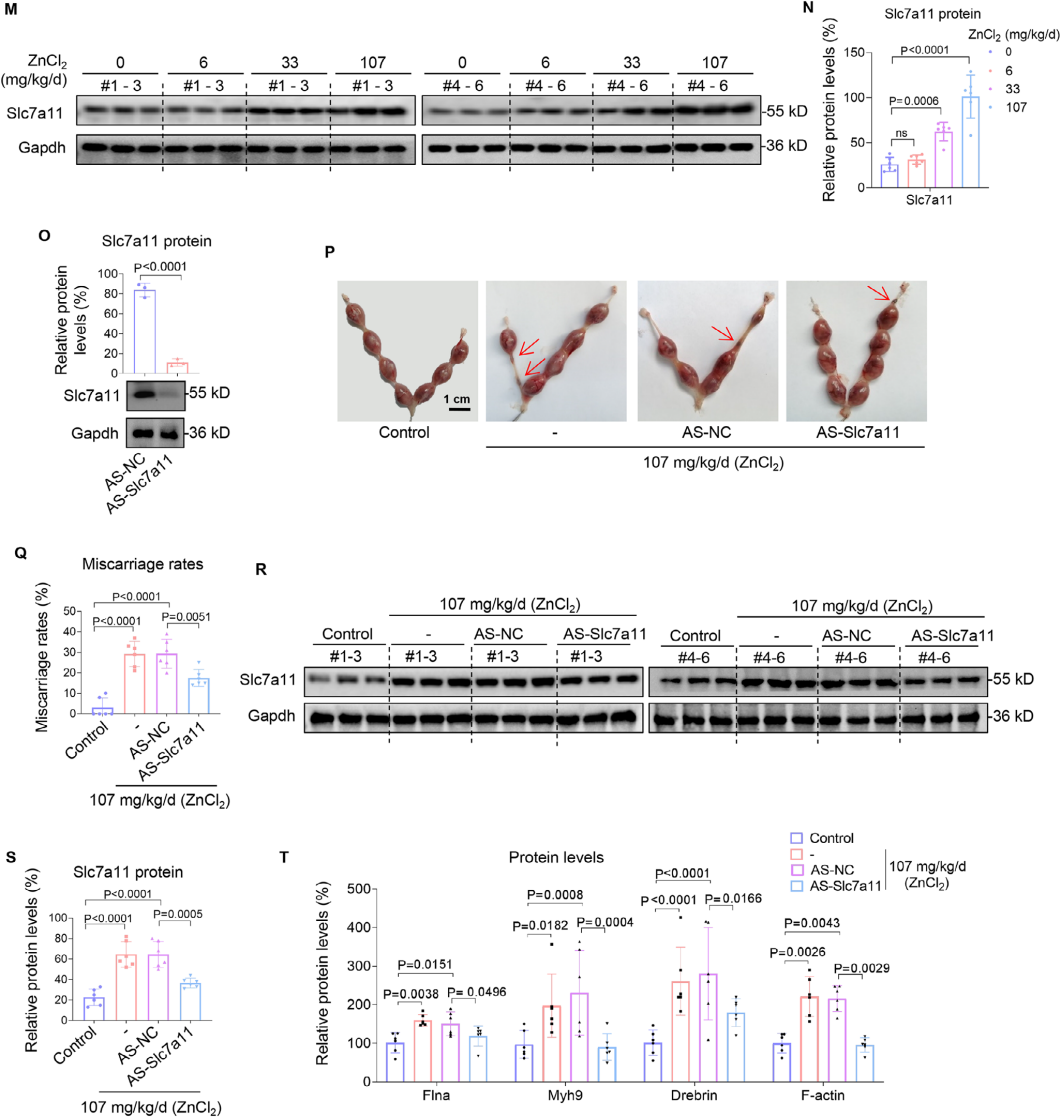

#### 3.3.2 Zn Exposure, SLC7A11 Expression Levels, and Miscarriage Were Positively Associated in UM Women Villous Tissues

In UM and HC villous tissues, we also found that the mRNA and protein levels of SLC7A11 were significantly highly in UM versus HC groups in both case‐control groups (Figure 4J,K; Figure S4I–M). Univariate and multivariate logistic regression analysis showed that higher levels of SLC7A11 mRNA in villous tissues were associated with unexplained miscarriage in both Groups 1 and 2 (Figure 4L, Table S7). Collectively, these results supported that the expression levels of SLC7A11 were higher in UM versus HC villous tissues, and the higher levels of SLC7A11 were positively associated with unexplained miscarriage.

To correlate the levels of Zn exposure and SLC7A11, Pearson correlation analysis showed that the levels of Zn in serum were positively correlated with the mRNA or protein levels of SLC7A11 in villous tissues in the UM group in both Groups 1 and 2 (Figure S4N–Q). The datapoints in the HC and UM groups were relative separated. Therefore, based on cellular results, Zn exposure might cause disulfidptosis in villous tissues by up‐regulating SLC7A11 expression levels, ultimately leading to miscarriage.

#### 3.3.3 Zn Exposure Caused Mouse Miscarriage by Promoting SLC7A11‐Mediated Disulfidptosis in ZnCl_2_‐Exposed Mouse Model

To explore the regulatory roles of SLC7A11 in disulfidptosis and miscarriage in this ZnCl_2_‐exposed mouse model, SLC7A11 expression levels were detected in mouse placental tissues. SLC7A11 mRNA and amino acid sequences were all conserved in rhesus, mouse, dog, and elephant (Figure S4R, Table S8). In the ZnCl_2_‐exposed mouse model, Zn exposure up‐regulated the mRNA and protein levels of SLC7A11 in mouse placental tissues (Figure 4M,N; Figure S4S). To investigate the causality whether SLC7A11 might induce miscarriage, we constructed a mouse miscarriage intervention model, in which ZnCl_2_‐exposed pregnant mice were intraperitoneally injected with AS‐Slc7a11 (knockdown of murine Slc7a11), with AS‐NC as a control. As validation, knockdown of murine Slc7a11 down‐regulated Slc7a11 mRNA and protein levels in mouse trophoblast cells (Figure 4O; Figure S4T). ZnCl_2_ exposure increased embryo adsorption and elevated miscarriage rates; however, knockdown of Slc7a11 efficiently reversed these changes (Figure 4P,Q). Furthermore, knockdown of Slc7a11 also down‐regulated the mRNA and protein levels of Slc7a11 (Figure 4R,S; Figure S4U), decreased the levels of cystine levels and NADP^+^/NADPH ratios (Figure S4V,W), and suppressed the formation of disulfide bonds in Flna, Myh9, Drebrin, and F‐actin (Figure 4T; Figure S4X) in placental tissues in ZnCl_2_‐exposed mouse model. Therefore, these results indicated that knockdown of murine Slc7a11 could reduce placental disulfidptosis and thus alleviate mouse miscarriage in the ZnCl_2_‐exposed mouse miscarriage model. In another word, Zn exposure up‐regulated Slc7a11 expression levels to cause placental disulfidptosis and ultimately induced mouse miscarriage.

### 3.4 Section IV Zn Exposure up‐Regulated SLC7A11 Levels at Both Transcription (by GATA1) and Post‐Transcription (by METTL1) Levels

#### 3.4.1 Zn Exposure Promoted GATA1‐Mediated SLC7A11 mRNA Transcription in Human Trophoblast Cells

**FIGURE 5:**
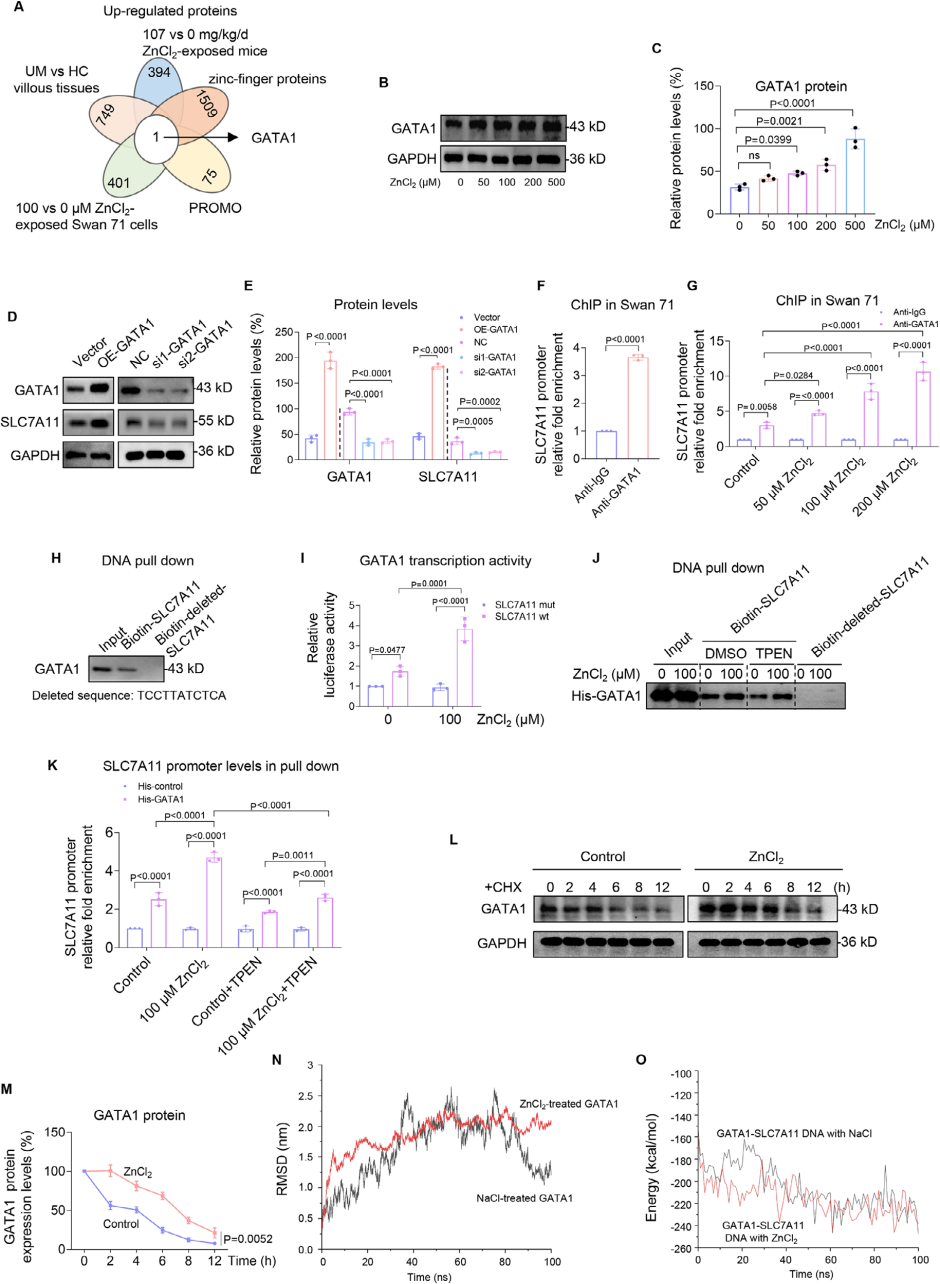
Zn exposure promoted GATA1‐mediated SLC7A11 mRNA transcription in human trophoblast cells. (A) In the intersection of the DEPs in UM versus HC villous tissues, 107 versus 0 mg/kg/day ZnCl_2_‐exposed mouse placental tissues, and 100 µm ZnCl_2_‐exposed trophoblast cells (three of which were shown in Figure S2D), the transcription factors of SLC7A11 predicted by PROMO, and Zn‐finger proteins (700 proteins), the sole GATA1 was identified. (B,C) Western blot analysis of GATA1 protein levels in 0, 50, 100, 200, or 500 µm ZnCl_2_‐exposed Swan 71 cells and its relative quantification. (D,E) Western blot analysis of the protein levels of GATA1 and SLC7A11 in Swan 71 cells with GATA1 overexpression or knockdown and their relative quantification. (F) GATA1 ChIP assay analysis of the SLC7A11 promoter region enriched by GATA1 in Swan 71 cells. (G) GATA1 ChIP assay analysis of the SLC7A11 promoter region enriched by GATA1 in ZnCl_2_‐exposed Swan 71 cells. (H) The protein levels of GATA1 pulled down by a biotin‐labeled DNA probe containing the SLC7A11 promoter region, but not the deletion in Swan 71 cells in DNA pull‐down assays. (I) Dual‐luciferase reporter assay analysis of the transcription activity of GATA1 on wild‐type (wt) or mutant (mut) promoter sequence of SLC7A11 in 100 µm ZnCl_2_‐exposed Swan 71 cells. (J) The protein levels of His‐GATA1 pulled down by biotin‐labeled DNA probe containing or without SLC7A11 promoter sequence in 100 µm ZnCl_2_‐exposed Swan 71 cells with TPEN treatment in DNA pull‐down assays. (K) The levels of SLC7A11 promoter sequence pulled down by His‐GATA1 in 100 µm ZnCl_2_‐exposed Swan 71 cells with TPEN treatment. (L,M) The protein levels of remaining GATA1 in 100 µm ZnCl_2_‐exposed Swan 71 cells with CHX (10 µm) treatment within 12 h and its relative quantification. (N) The root mean square deviation (RMSD) analysis of the protein structural stability of GATA1 (UniProt ID: P15976) treated with 200 µm ZnCl_2_ (with NaCl as control) by molecular dynamics (MD) simulations using GROMACS within 100 ns. (O) The binding energy of ZnCl_2_‐ or NaCl‐treated (as control group) GATA1‐SLC7A11 promoter region was analyzed by gmx_MMPBSA v1.6.1 (single trajectory method).

Subsequently, we explored how Zn exposure up‐regulated the expression levels of SLC7A11. First, we considered its mRNA transcription. In the intersection of proteomic data of UM versus HC villous tissues, 107 versus 0 mg/kg/day ZnCl_2_‐exposed mice placental tissues, and ZnCl_2_‐exposed trophoblast cells, the transcription factors of SLC7A11 predicted by PROMO, as well as Zn‐finger proteins (1510 proteins), the sole GATA1 was identified (Figure 5A). Here, we considered the Zn‐finger protein because Zn binds with them and might alter their conformation and functions with varying Zn exposure levels. Therefore, this inspired us to explore whether Zn exposure might regulate GATA1 expression levels and its functions. Experimentally, Zn exposure up‐regulated the mRNA and protein levels of GATA1 (Figure 5B,C; Figure S5A). Overexpression of GATA1 up‐regulated, whereas knockdown of GATA1 down‐regulated, the mRNA and protein levels of SLC7A11 (Figure 5D,E; Figure S5B–E). Treatment with 4‐HRP (an inhibitor of GATA1 [57]) down‐regulated the protein levels of GATA1 and SLC7A11 (Figure S5F–H). GATA1 ChIP assays showed that the promoter region of SLC7A11 could be enriched by GATA1 (Figure 5F), which was further enhanced with Zn exposure (Figure 5G) but was weakened with 4‐HRP treatment (Figure S5I). DNA pulldown assays also showed that a DNA probe containing the wild‐type promoter sequence of SLC7A11 could, but the probe without this sequence (Table S8) could not, pull down GATA1 protein (Figure 5H). Dual‐luciferase assays showed that GATA1 showed transcription activity at the wild‐type, but not the mutant, sequence of the SLC7A11 promoter region, a transcription activity that was further enhanced with Zn exposure (Figure 5I). To further identify the binding functions of GATA, we purified His‐tag labeled GATA1 protein (His‐GATA1) from *E. Coli*. DNA pull‐down assays showed that His‐GATA1 protein could be pull down by a DNA probe containing the promoter sequence of SLC7A11, but not by the probe without this sequence (Table S9), which was further enhanced with ZnCl_2_ treatment but was reduced with TPEN co‐treatment (Zn ion chelating agent) (Figure 5J). Meanwhile, the purified His‐GATA1 protein could also pull down DNA containing the SLC7A11 promoter region, which was further enhanced with ZnCl_2_ treatment but was reduced with TPEN co‐treatment (Figure 5K). Therefore, the presence of Zn enhanced the binding between the GATA1 protein and the promoter region of SLC7A11. Taken together, these results suggested that GATA1 acted as a transcription factor of SLC7A11 and promoted its transcription. Zn exposure not only up‐regulated GATA1 expression levels but also enhanced its protein conformation, both of which promoted GATA1‐mediated SLC7A11 transcription and up‐regulated SLC7A11 expression levels.

Meanwhile, we also found that Zn exposure enhanced the protein stability of GATA1 (Figure 5L,M). Since GATA1 is a Zn‐binding protein [58], next, we explored whether Zn might regulate GATA1 protein structure stability by molecular dynamic (MD) stimulation. The 3D structure of GATA1 (UniProt ID: P15976) was constructed by AlphaFold3. The Ramachandran plot showed the strong overall quality of the GATA1 protein 3D structure (Figure S5J). Then, MD simulations of ZnCl_2_‐treated GATA1 (with NaCl‐treated GATA1 as control) showed that the values of root mean square deviation (RMSD) of Zn‐treated GATA1 were lower and had less fluctuations (especially in 40–100 ns) than the control protein (Figure 5N), showing that Zn exposure might significantly enhance the structural stability of GATA1 protein. Collectively, these results demonstrated that Zn exposure might enhance the structural stability of GATA1 protein, which might explain that Zn exposure increased GATA1 protein stability.

Subsequently, we further investigated the effects of Zn exposure on the binding between the GATA1 protein and the SLC7A11 promoter region. The complex structures of the DNA‐binding region (residues 200–318) of GATA1 and the SLC7A11 promoter region (5’‐TCCTTATCTCA‐3’) were constructed by AlphaFold3 and optimized by MD simulations. The GATA1‐SLC7A11 promoter complex contained 12 H‐bonds, 8 salt bridges, and 1 π‐cation interaction (Figure S5K), supporting that GATA1 could bind with the SLC7A11 promoter region. Then, we compared the structural difference between the ZnCl_2_‐treated GATA1‐SLC7A11 promoter region and the NaCl‐treated GATA1‐SLC7A11 promoter region as a control group. Compared with the control group, Zn‐treated GATA1‐SLC7A11 promoter region had more H‐bonds (25 vs. 16), more salt bridges (11 vs. 6), and more π‐cation interaction (2 vs. 0) (Figure S5L,M), suggesting that Zn exposure increased the binding stability of GATA1 with the SLC7A11 promoter region. The average binding energy of Zn‐treated GATA1‐SLC7A11 promoter region was −214.97 kcal/mol, lower than −205.46 kcal/mol for the control group (Figure 5O, Table S10), indicating that Zn exposure strengthened the binding between GATA1 and SLC7A11 promoter region, which might explain that Zn exposure promoted GATA1‐mediated SLC7A11 transcription.

To explore the upstream of GATA1, as analyzed by PROMO, SP1 might be a transcription factor of GATA1. In trophoblast cells (without Zn exposure), overexpression of SP1 up‐regulated, whereas knockdown of SP1 down‐regulated, the mRNA and protein levels of GATA1 (Figure S5N–S). SP1 ChIP assays showed that the promoter region of GATA1 could be enriched by SP1 (Figure S5T). Moreover, Zn exposure up‐regulated the mRNA and protein levels of SP1 (Figure S5U–W). Therefore, these results suggested that SP1 acted as a transcription factor of GATA1 and promoted GATA1 transcription.

It has been reported that GATA1 up‐regulates zinc importer SLC39A8 to elevate cellular Zn levels in erythroblast cells [59]; and GATA1 and heme collaboratively up‐regulate zinc exporter SLC30A1 to reduce cellular Zn levels [59]. In trophoblast cells, we also found that overexpression of GATA1 increased, whereas down‐regulation of GATA1 reduced, cellular Zn levels (Figure S5X,Y). Treatment with 4‐HRP (an inhibitor of GATA1 [57]) reduced cellular Zn levels (Figure S5Z). These results suggested that both Zn and GATA1 might form a positive regulatory feedback loop.

#### 3.4.2 Zn Exposure Increased METTL1‐Mediated m7G Modification Levels on SLC7A11 mRNA and Promoted SLC7A11 mRNA Stability

**FIGURE 6:**
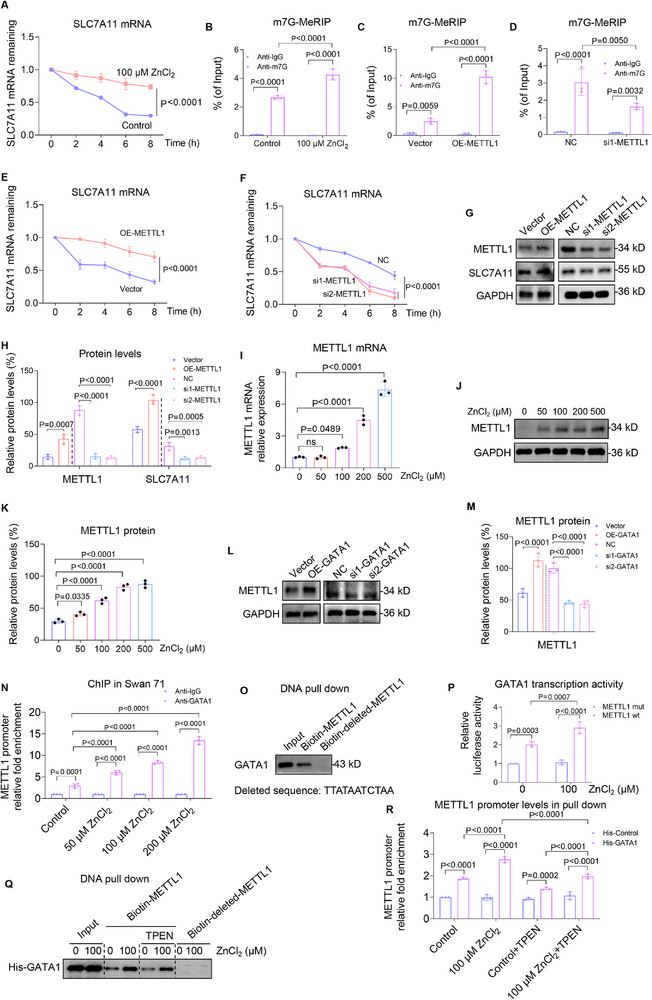
Zn exposure increased METTL1‐mediated m7G modification levels on SLC7A11 mRNA and promoted SLC7A11 mRNA stability. (A) The levels of the remaining SLC7A11 mRNA in 100 µm ZnCl_2_‐exposed Swan 71 cells with treatment with 5 µg/mL actinomycin D within 48 h. (B) MeRIP analysis of the m7G modification levels on SLC7A11 mRNA in 100 µm ZnCl_2_‐exposed Swan 71 cells. (C) MeRIP analysis of the m7G modification levels on SLC7A11 mRNA in Swan 71 cells with METTL1 overexpression. (D) MeRIP analysis of the m7G modification levels on SLC7A11 mRNA in Swan 71 cells with METTL1 knockdown. (E) The levels of the remaining SLC7A11 mRNA in Swan 71 cells with METTL1 overexpression. (F) The levels of the remaining SLC7A11 mRNA in Swan 71 cells with METTL1 knockdown. (G,H) Western blot analysis of the protein levels of METTL1 and SLC7A11 in Swan 71 cells with METTL1 overexpression or knockdown and their relative quantification. (I) METTL1 mRNA levels in 0, 50, 100, 200, or 500 µm ZnCl_2_‐exposed Swan 71 cells. (J,K) METTL1 protein levels in 0, 50, 100, 200, or 500 µm ZnCl_2_‐exposed Swan 71 cells and its relative quantification. (L,M) Western blot analysis of METTL1 protein levels in Swan 71 cells with GATA1 overexpression or knockdown and its relative quantification. (N) GATA1 ChIP assay analysis of METTL1 promoter region enriched by GATA1 in ZnCl_2_‐exposed Swan 71 cells. (O) The protein levels of His‐GATA1 pulled down by a biotin‐labeled DNA probe containing or without the METTL1 promoter sequence in DNA pull‐down assays. (P) Dual‐luciferase reporter assay analysis of the transcription activity of GATA1 using wild‐type (wt) or mutant (mut) promoter sequence of METTL1 in 100 µm ZnCl_2_‐exposed Swan 71 cells. (Q) The protein levels of His‐GATA1 pulled down by biotin‐labeled DNA probe containing or without METTL1 promoter sequence in 100 µm ZnCl_2_‐exposed Swan 71 cells with TPEN treatment in DNA pull‐down assays. (R) The levels of the METTL1 promoter sequence pulled down by His‐GATA1 in 100 µm ZnCl_2_‐exposed Swan 71 cells with TPEN treatment.

Second, we also investigated whether Zn exposure might also affect SLC7A11 mRNA stability. We found that Zn exposure enhanced SLC7A11 mRNA stability (Figure 6A). It has been reported that m7G modification on mRNA increases mRNA stability. Predicted by m7GHub V2.0 (www.rnamd.org/m7GHub2/index.html), there is the presence of m7G modification sites on SLC7A11 mRNA. M7G MeRIP assays confirmed the presence of m7G modification on SLC7A11 mRNA; and Zn exposure further increased the m7G modification levels on SLC7A11 mRNA (Figure 6B). METTL1 is a typical m7G modification writer and was also present in the intersection of the proteomic data of UM versus HC villous tissues, 107 versus 0 mg/kg/day ZnCl_2_‐exposed mouse placental tissues, and 100 versus 0 µm ZnCl_2_‐exposed trophoblast cells (Figure S2D). First, overexpression of METTL1 (Figure S6A) increased m7G modification on SLC7A11 mRNA (Figure 6C), increased SLC7A11 mRNA stability (Figure 6E), and up‐regulated SLC7A11 mRNA and protein levels (Figure 6G,H; Figure S6B); whereas knockdown of METTL1 gave the opposite results (Figure 6D,F–H; Figure S6C,D). Second, Zn exposure up‐regulated METTL1 mRNA and protein levels (Figure 6I–K), as well as m7G modification levels on SLC7A11 mRNA (Figure 6B). Therefore, these results confirmed that Zn exposure up‐regulated METTL1 expression levels, increased METTL1‐mediated m7G modification levels on SLC7A11 mRNA, and enhanced its mRNA stability, and finally up‐regulated SLC7A11 expression levels.

Moreover, we further explored how Zn exposure up‐regulated METTL1 expression levels. Predicted by PROMO, GATA1 might also serve as a transcription factor of METTL1 (Figure S6E). Overexpression of GATA1 up‐regulated, whereas knockdown of GATA1 down‐regulated, the mRNA and protein levels of GATA1 and METTL1 (Figure 6L,M; Figure S6F–G). Furthermore, treatment with 4‐HRP (GATA1 inhibitor) down‐regulated the protein levels of METTL1 (Figure S6H,I). GATA1 ChIP assays showed the promoter region of METTL1 could be enriched by GATA1 protein, which was further enhanced with Zn treatment (Figure 6N) but was weakened with 4‐HRP treatment (Figure S6J). DNA pulldown assays showed that a DNA probe containing the promoter sequence of METTL1 could, but a DNA probe without this sequence (Table S9) could not, pull down GATA1 protein (Figure 6O). Dual‐luciferase assays showed that GATA1 showed transcription activity at wild‐type, but not mutant, sequence in the METTL1 promoter region, a transcription activity that was further enhanced with Zn treatment (Figure 6P). DNA pull‐down assays using the purified His‐GATA1 protein showed that His‐GATA1 protein could be pull down by a DNA probe containing the promoter sequence of METTL1, but could not by the probe without this sequence (Table S9), which was further enhanced with ZnCl_2_ treatment but was reduced with TPEN co‐treatment (Figure 6Q). In turn, the purified His‐GATA1 protein could also pull down a DNA probe containing the SLC7A11 promoter region sequence, which was further enhanced with ZnCl_2_ treatment but was reduced with TPEN co‐treatment (Figure 6R). Therefore, the presence of Zn enhanced the binding between the GATA1 protein and the promoter region of METTL1. Taken together, these results suggested that GATA1 acted as a transcription factor of METTL1 and promoted its transcription. Combined with the above results, Zn exposure not only up‐regulated GATA1 expression levels but also enhanced its protein conformation, both of which promoted GATA1‐mediated METTL1 transcription and up‐regulated its expression levels.

We also studied the effect of Zn on the binding of the GATA1 protein with the METTL1 promoter region by MD stimulation. First, the complex structures of the DNA‐binding region of GATA1 (residues 200–318) and the METTL1 promoter region (5’‐TTATAATCTAA‐3’) were constructed by AlphaFold3 and optimized by MD simulations. The complex contained 15 H‐bonds, 6 salt bridges, and 1 π‐cation interaction in the GATA1‐METTL1 promoter complex (Figure S6K), supporting that GATA1 could bind with the METTL1 promoter region. Then, we compared the structural difference between the ZnCl_2_‐treated GATA1‐METTL1 promoter region and the NaCl‐treated GATA1‐METTL1 promoter region as a control group. Compared with the control group, the Zn‐treated group had more H‐bonds (26 vs. 16) interactions (Figure S6L,M). The average binding energy of ZnCl_2_‐treated GATA1‐METTL1 promoter region was −210.98 kcal/mol, lower than −202.84 kcal/mol for NaCl‐treated control group (Figure S6N, Table S11), indicating that Zn exposure strengthened the binding between GATA1 and METTL1 promoter region, which might explain that Zn exposure promoted GATA1‐mediated METTL1 transcription.

#### 3.4.3 Knockdown of GATA1 or METTL1 Suppressed Disulfidptosis in ZnCl_2_‐Exposed Trophoblast Cells

**FIGURE 7:**
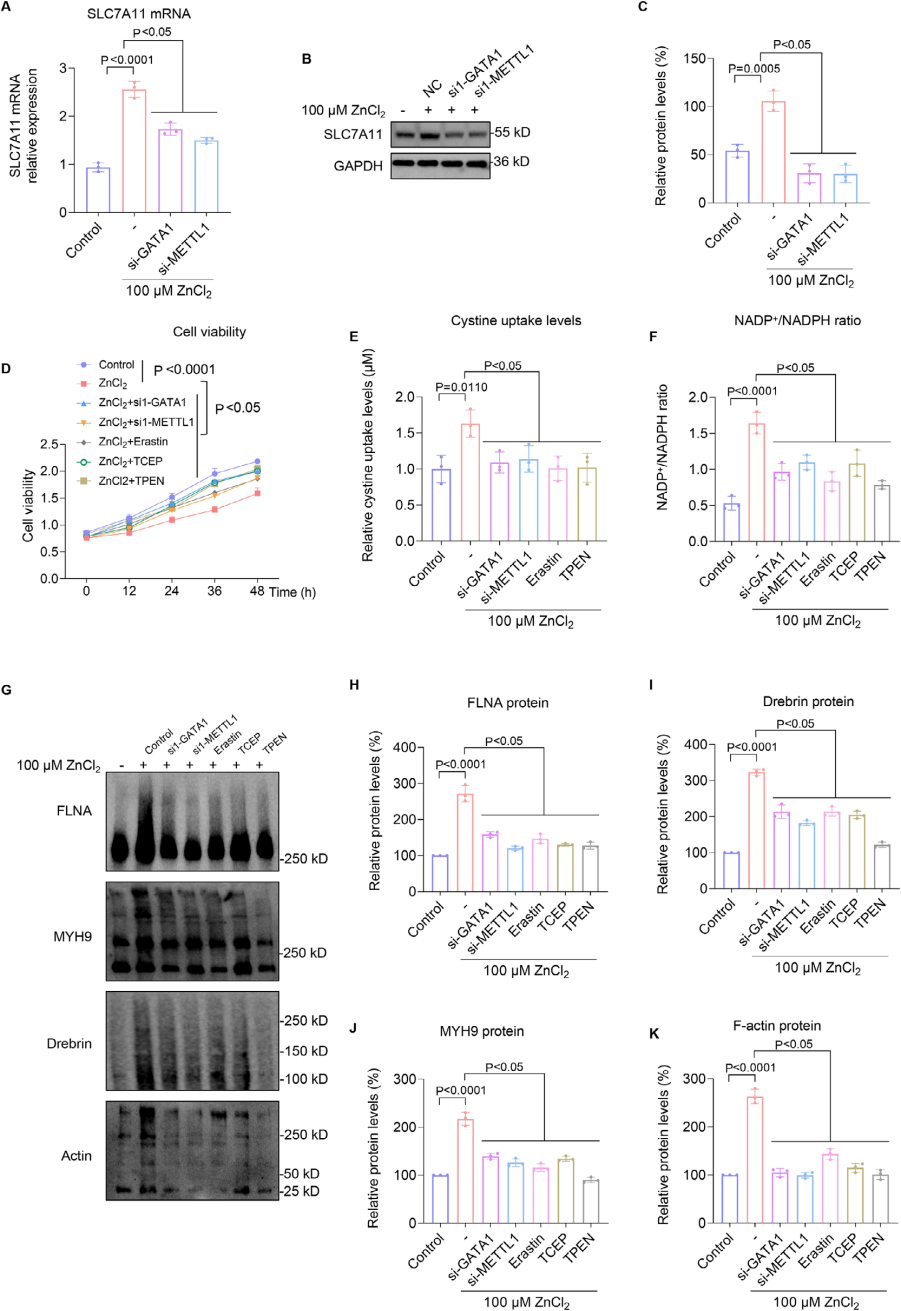
Knockdown of GATA1 or METTL1 suppressed disulfidptosis in ZnCl_2_‐exposed trophoblast cells. (A) RT‐qPCR analysis of SLC7A11 mRNA levels in 100 µm ZnCl_2_‐exposed Swan 71 cells with GATA1 or METTL1 knockdown. (B,C) Western blot analysis of SLC7A11 protein levels in 100 µm ZnCl_2_‐exposed Swan 71 cells with knockdown of GATA1 or METTL1 and its relative quantification. (D) CCK8 assay analysis of cell viability of 100 µm ZnCl_2_‐exposed Swan 71 cells with knockdown of GATA1 or METTL1 or treatment with Erastin, TCEP, or TPEN within 24 h. (E) Cystine levels in 100 µm ZnCl_2_‐exposed Swan 71 cells with knockdown of GATA1 or METTL1 or treatment with Erastin or TPEN. (F) The NADP^+^/NADPH ratios in 100 µm ZnCl_2_‐exposed Swan 71 cells with knockdown of GATA1 or METTL1 or treatment with Erastin, TCEP, or TPEN. (G–K) Western blot analysis of Cys‐crosslinked FLNA, MYH9, Drebrin, and F‐actin in 100 µm ZnCl_2_‐exposed Swan 71 cells with knockdown of GATA1 or METTL1 or treatment with Erastin, TCEP, or TPENGATA1 or METTL1 and their relative quantification.

Having known the roles of GATA1 or METTL1 in the regulation of SLC7A11 expression levels, we further explored whether knockdown of GATA1 or METTL1 might reduce disulfidptosis levels in ZnCl_2_‐exposed trophoblast cells. First, ZnCl_2_ exposure up‐regulated the mRNA and protein levels of SLC7A11; and co‐knockdown of GATA1 or METTL1 down‐regulated SLC7A11 mRNA and protein levels (Figure 7A–C). Zn exposure reduced cell viability, co‐knockdown of GATA1 or METTL1 could restore (i.e., increase) cell viability; and co‐treatment with Erastin (SLC7A11 inhibitor), TCEP (reductant), or TPEN (Zn chelator) could also restore (i.e., increase) cell viability (Figure 7D). Zn exposure increased the levels of cystine intake and NADP^+^/NADPH ratios, and co‐knockdown of GATA1 or METTL1 or co‐treatment with Erastin, TCEP, or TPEN could reduce their levels (Figure 7E,F). Zn exposure promoted the formation of disulfide bonds in FLNA, MYH9, Drebrin, and F‐actin proteins; and co‐knockdown of GATA1 or METTL1 suppressed their formation (Figure 7G–K). Collectively, these results confirmed that Zn exposure caused trophoblast cell disulfidptosis by up‐regulated the expression levels of GATA1 and METTL1.

### 3.5 Section V GATA1/METTL1/SLC7A11 Axis was Highly Expressed in UM versus HC Women Villous Tissues and in ZnCl_2_‐Exposed Mouse Placental Tissues

#### 3.5.1 GATA1/METTL1/SLC7A11 Axis was Highly Expressed in UM versus HC Women Villous Tissues

To explore the potential association between GATA1 or METTL1 expression levels and unexplained miscarriage, we detected their mRNA and protein levels in HC and UM women villous tissues in both case‐control Groups 1 and 2. The mRNA and protein levels of GATA1 or METTL1 were highly in UM versus HC groups in both groups (Figure 8A,B; Figure S7A–F). Univariate and multivariate logistic regression analysis showed that higher levels of GATA1 or METTL1 were associated with unexplained miscarriage and could act as a risk factor for unexplained miscarriage (Figure 8C, Table S7). Collectively, these results confirmed that the expression levels of GATA1 or METTL1 were positively associated with unexplained miscarriage.

FIGURE 8GATA1/METTL1/SLC7A11 axis was highly expressed in UM versus HC women villous tissues and in ZnCl_2_‐exposed mouse placental tissues. (A,B) The protein levels of GATA1 and METTL1 in HC and UM women villous tissues and their relative quantification (each *n* = 12). (C) Univariate and multivariate logistic regression analysis of the association of GATA1 and METTL1 mRNA in villous tissues with miscarriage in Group 1 (*n* = 50) and Group 2 (*n* = 40). (D) GATA1 ChIP assay analysis of SLC7A11 promoter region enriched by GATA1 in HC and UM villous tissues (*n* = 6). (E) GATA1 ChIP assay analysis of METTL1 promoter region enriched by GATA1 in HC and UM villous tissues (*n* = 6). (F,G) The protein levels of GATA1 that were pulled down by biotin‐labeled DNA probe containing SLC7A11 or METTL1 promoter sequence in UM versus HC villous tissues in Group 1 in DNA pull‐down assays and their relative quantification (each *n* = 6). (H) MeRIP analysis of the m7G modification levels on SLC7A11 mRNA in UM versus HC villous tissues in Group 1. (I) The protein levels of murine Gata1 and Mettl1 in 0, 6, 33, or 107 mg/kg/day ZnCl_2_‐exposed mouse placental tissues (each *n* = 6). (J) Gata1 ChIP assay analysis of the levels of Mettl1 promoter region enriched by Gata1 in 0, 6, 33, or 107 mg/kg/day ZnCl_2_‐exposed mouse placental tissues (each *n* = 6). (K) Gata1 ChIP assay analysis of the levels of Slc7a11 promoter region enriched by Gata1 in 0, 6, 33, or 107 mg/kg/day ZnCl_2_‐exposed mouse placental tissues (each *n* = 6). (L) MeRIP analysis of the m7G modification levels on Slc7a11 mRNA in 0, 6, 33, or 107 mg/kg/day ZnCl_2_‐exposed mouse placental tissues (each *n* = 6).

**Figure:**
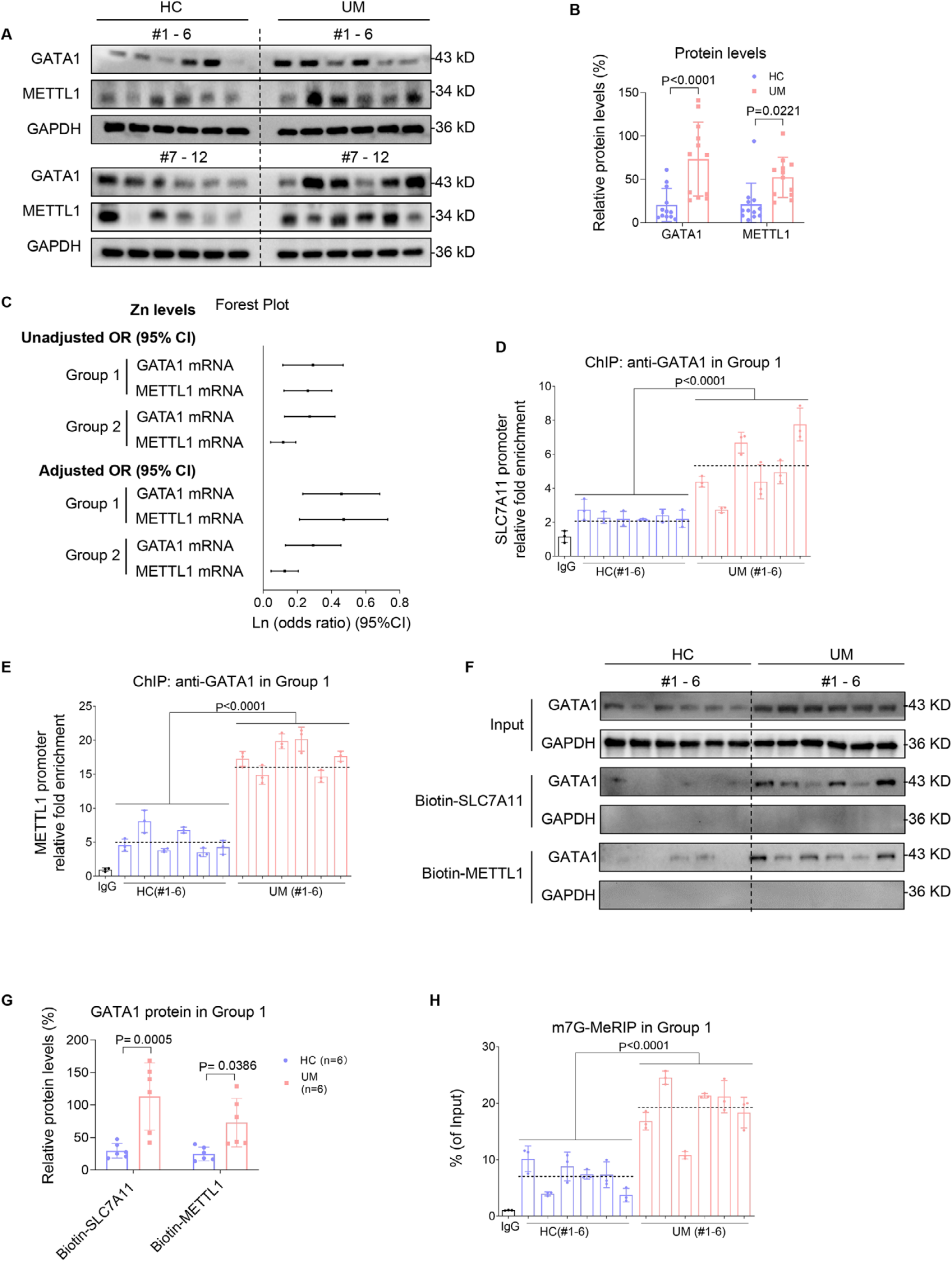

**Figure:**
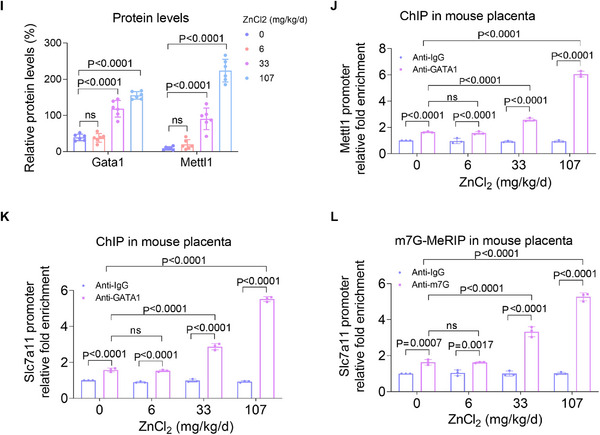

To explore the mechanism underlying the up‐regulation of SLC7A11 expression levels in villous tissues, GATA1 ChIP assays showed that the levels of SLC7A11 or METTL1 promoter regions enriched by GATA1 were higher in UM versus HC villous tissues (Figure 8D,E; Figure S7G,H). DNA pull‐down assays showed that the protein levels of GATA1 that were pulled down by DNA probe containing promoter regions of SLC7A11 or METTL1 were higher in UM versus HC group (Figure 8F,G; Figure S7I,J). M7G MeRIP assays showed that the levels of m7G modification on SLC7A11 mRNA were higher in UM versus HC group (Figure 8H; Figure S7K). Combined with the cellular results, these tissue results showed that GATA1‐mediated SLC7A11 or METTL1 transcription and METTL1‐mediated SLC7A11 mRNA stability were promoted in UM versus HC villous tissues, explaining higher expression levels of SLC7A11 in the UM versus the HC group.

Subsequently, the correlation among the levels of Zn exposure, GATA1, METTL1, and SLC7A11 expression levels in villous tissues was analyzed by Pearson correlation analysis. Zn levels were positively correlated with GATA1 or METTL1 mRNA or protein levels in UM villous tissues (Figure S7L–S). Moreover, the protein levels of GATA1 or METTL1 were also positively correlated with those of SLC7A11 in UM villous tissues (Figure S7T–W). The datapoints in the HC and UM groups were relative separated. Therefore, combined with the cellular results, we proposed that Zn exposure caused disulfidptosis in villous tissues to induce miscarriage by up‐regulating the GATA1/METTL1/SLC7A11 axis.

#### 3.5.2 Murine Gata1/Mettl1/Slc7a11 Axis Was Highly Expressed in ZnCl_2_‐Exposed Mouse Placental Tissues

To explore the roles of Gata1 and Mettl1 in the regulation of Slc7a11 expression levels in the mouse model, we also detected their expression levels in ZnCl_2_‐exposed mouse placental tissues. The mRNA and amino acid sequences of GATA1 and METTL1 were all conserved in rhesus, mouse, dog, and elephant (Figure S8A, Table S8). ZnCl_2_ exposure up‐regulated the mRNA and protein levels of Gata1 and Mettl1 in mouse placental tissues (Figure 8I; Figure S8B–D). Gata1 ChIP assays showed that the levels of Slc7a11 or Mettl1 promoter regions that were enriched by Gata1 protein were higher in ZnCl_2_‐exposed mouse placental tissues (Figure 8J,K). m7G MeRIP assays also showed that the levels of m7G modification on Slc7a11 mRNA were higher in ZnCl_2_‐exposed mouse placental tissues (Figure 8L). These results were consistent with those in ZnCl_2_‐exposed trophoblast cells and in UM versus HC villous tissues. Collectively, these results demonstrated that Gata1‐mediated Slc7a11 or Mettl1 transcription and Mettl1‐meidated Slc7a11 mRNA stability were enhanced in ZnCl_2_‐exposed mouse placental tissues, explaining the up‐regulated Slc7a11 expression levels in ZnCl_2_‐exposed mouse placental tissues.

### 3.6 Section VI Miscarriage Treatment

#### 3.6.1 Miscarriage Treatment by Targeting Gata1, Mettl1, or NADPH in ZnCl_2_‐Exposed Mouse Model

**FIGURE 9:**
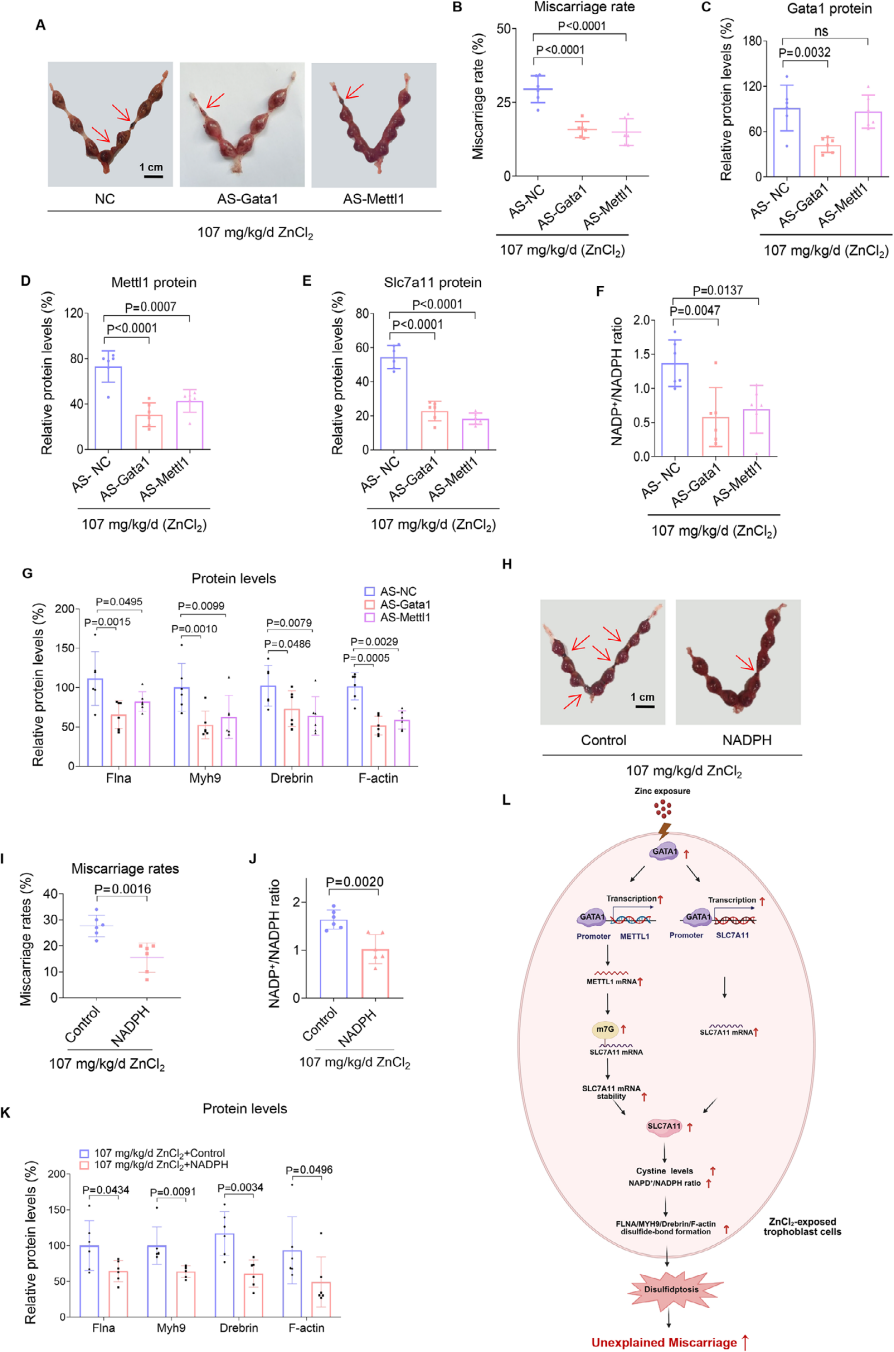
Miscarriage treatment by targeting Gata1, Mettl1, or NADPH. (A,B) Embryo resorption (indicated by red arrows) and the average miscarriage rates in 107 mg/kg/day ZnCl_2_‐exposed mice with knockdown of Gata1 or Mettl1 (each *n* = 6). (C–E) The protein levels of Gata1, Mettl1, and Slc7a11 in 107 mg/kg/day ZnCl_2_‐exposed mouse placental tissues with knockdown of Gata1 or Mettl1 (*n* = 6). (F) The NADP^+^/NADPH ratios in 107 mg/kg/day ZnCl_2_‐exposed mouse placental tissues with knockdown of Gata1 or Mettl1 (*n* = 6). (G) The protein levels of Cys‐crosslinked Flna, Myh9, Drebrin, and F‐actin in 107 mg/kg/day ZnCl_2_‐exposed mouse placental tissues with knockdown of Gata1 or Mettl1. (H,I) Embryo resorption (indicated by red arrows) and the average miscarriage rates in 107 mg/kg/day ZnCl_2_‐exposed mice with NADPH supplement (each *n* = 6). (J) The NADP^+^/NADPH ratios in 107 mg/kg/day ZnCl_2_‐exposed mouse placental tissues with NADPH supplement (each *n* = 6). (K) The protein levels of Cys‐crosslinked Flna, Myh9, Drebrin, and F‐actin in 107 mg/kg/day ZnCl_2_‐exposed mouse placental tissues with NADPH supplement. (L) The proposed regulatory mechanism.

Having known the essential roles of GATA1 and METTL1 in disulfidptosis and miscarriage, finally, we investigated the roles of GATA1 and METTL1 in the treatment against miscarriage. For this aim, we constructed two mouse miscarriage intervention models, in which ZnCl_2_‐exposed pregnant mice were intraperitoneally injected with AS‐Gata1 (knockdown of Gata1) or AS‐Mettl1 (knockdown of Mettl1), with AS‐NC as a control. As validation, knockdown of murine Gata1 or Mettl1 down‐regulated their mRNA and protein levels in mouse trophoblast cells (Figure S9A–E). In ZnCl_2_‐exposed pregnant mice, knockdown of GATA1 or METTL1 reduced embryo adsorption and miscarriage rates (Figure 9A,B). Analysis of the placental tissues showed that knockdown of Gata1 or Mettl1 reduced the mRNA and protein levels of Gata1, Mettl1, and Slc7a11 in ZnCl_2_‐exposed mouse placental tissues (Figure 9C–E; Figure S9F–I), except for that knockdown of Mettl1 did not affect the mRNA or protein levels of Gata1 (Figure 9C; Figure S9F,I). Knockdown of Gata1 or Mettl1 reduced the levels of cystine intake (Figure S9J) and NADP^+^/NADPH ratios (Figure 9F) and also suppressed the formation of disulfide bonds in Flna, Myh9, Drebrin, and F‐actin proteins (Figure 9G; Figure S9K). Collectively, these results indicated that knockdown of the murine Gata1/Mettl1/Slc7a11 axis could efficiently suppress placental disulfidptosis and thus alleviate mouse miscarriage in this ZnCl_2_‐exposed mouse miscarriage model.

Since the ratios of NADP^+^/NADPH were increased in the ZnCl_2_‐exposed mouse miscarriage model, we further explored whether supplement with NADPH might also be used for treatment against miscarriage. To test this, we constructed another mouse miscarriage treatment model, in which ZnCl_2_‐exposed pregnant mice were intraperitoneally injected with NADPH, with normal saline as solvent (Figure S9L). Supplement with NADPH could also efficiently reduce embryo adsorption and miscarriage rates in this ZnCl_2_‐exposed mouse model (Figure 9H,I). Analysis of the placental tissues showed that supplement with NADPH up‐regulated NADPH levels and thus reduced the ratios of NADP^+^/NADPH (Figure 9J), but did not alter the protein levels of murine Slc7a11 (Figure S9M,N). Supplement with NADPH also suppressed the formation of disulfide bonds in Flna, Myh9, Drebrin, and F‐actin proteins (Figure 9K; Figure S9O). Therefore, these results indicated that supplement with NADPH could also reduce mouse placental disulfidptosis to alleviate mouse miscarriage in this ZnCl_2_‐exposed mouse miscarriage model.

## 4 Discussion

### 4.1 Zn Exposure Causes Disulfidptosis to Induce Miscarriage

Zn has dual effects on antioxidant capacity and acts as a pro‐oxidant agent under overload conditions [60]. Elemental Zn < 40 mg/day (tolerable upper intake level, UL) shows a greater reducing effect on MDA levels, and higher doses > 40 mg/day Zn are related to oxidative stress conditions [61]. Zn overload inhibits the activity of some enzymes in glycolysis reactions [60] and thus reduces NADH levels, leading to the cessation of ATP production and the production of reactive oxygen species (ROS) [60]. Moreover, intake of metallic dietary supplements containing 300 mg Zn per day might induce anemia, neutropenia, and immune suppression [22]. Epidemiological studies have shown that excessive Zn exposure during early pregnancy is associated with a higher risk of adverse pregnancy outcomes, such as preterm birth [23, 27]. Higher maternal Zn levels have been associated with intrauterine growth restriction (IUGR), giving babies with smaller gestational age [62]. Other studies also show that higher Zn levels in maternal circulation are associated with unfavorable pregnancy outcomes [63]. For example, plasma Zn levels are significantly higher in pregnant women with preeclampsia (PE) compared with those with healthy pregnancies [63]. Maternal serum Zn levels are also higher in women who have babies with a smaller gestational age compared with those who have healthy pregnancies [64, 65]. The elevated maternal Zn levels suppress placental trophoblast proliferation [66]. Animal model studies also show that high levels of Zn exposure disrupt spermatogenic cells and increase abnormal sperm morphology in male mice, and reduce sperm count and motility in male rats [28]. ZnO nanoparticles (NPs), which release Zn ions from NPs [29], inhibit follicle growth, limit oocyte differentiation, damage ovarian cells, cause adverse pregnancy outcomes, and even cause neurological damage in offspring [30]. However, whether and how Zn exposure during pregnancy might induce unexplained miscarriage remains completely unknown. In this study, we find for the first time that Zn levels are significantly higher in UM versus HC serum samples, and higher Zn levels are positively associated with unexplained miscarriage in two case‐control groups in two different cities (with a distance of 2350 km). Meanwhile, the levels of disulfidptosis are higher in the UM versus the HC group. Higher levels of Zn in serum, higher levels of disulfidptosis in villous tissues, and unexplained miscarriage are positively associated. Moreover, in the mouse model, we confirm that Zn exposure causes disulfidptosis in mouse placental tissue and thus induces mouse miscarriage, and reducing placental disulfidptosis could efficiently relieve mouse miscarriage. Cellular assays further discover that Zn exposure leads to in trophoblast cell disulfidptosis, which might induce miscarriage. Therefore, it is the first time that we confirm that Zn exposure leads to disulfidptosis and thus induces unexplained miscarriage based on the epidemiological analysis, mouse assays, and cellular mechanistic study.

In our recent studies, we also find that hypoxia leads to ferroptosis to induce miscarriage [17]; BaP (benzo(a)pyrene) or BPDE (benzo(a)pyrene‐7,8‐dihydrodiol‐9,10‐epoxide) exposure leads to ferroptosis in vascular endothelial cells, inhibits their angiogenesis, and thus induces miscarriage [11]. Moreover, BaP/BPDE exposure also inhibits trophoblast cell migration/invasion, suppresses homologous recombination repair, or induces pyroptosis, any of which might induce unexplained miscarriage [7, 9, 13]. Nanoplastics can also inhibit trophoblast cell migration/invasion and migrasome formation or lead to apoptosis, which further induces miscarriage [15, 16]. Copper exposure also leads to mouse placental cuproptosis and induces miscarriage [43]. Here, we find that Zn exposure causes disulfidptosis to induce miscarriage, discovering a new risk factor and a novel pathogenesis for unexplained miscarriage.

### 4.2 Novel Biological Mechanisms of Zn Exposure‐Caused Disulfidptosis

SLC7A11 plays a crucial role in cystine uptake and depletion of intracellular NADPH, leading to accumulation of disulfide molecules, which further triggers the formation of disulfide bonds between actin cytoskeleton proteins, eventually resulting in disulfidptosis [31, 32]. Therefore, SLC7A11 acts as a critical cystine transporter that effectively regulates disulfidptosis in kidney cancer cells [31]. In this study, we find that Zn exposure up‐regulates GATA1 expression levels, which promotes GATA1‐mediated transcription of METTL1 and SLC7A11 and thus up‐regulates their mRNA levels (Figure 9L). Meanwhile, METTL1 also performs m7G modification on SLC7A11 mRNA and enhances SLC7A11 mRNA stability. Therefore, Zn exposure up‐regulates SLC7A11 mRNA levels at both transcription and post‐transcription levels, as well as its protein levels, which further promotes cystine uptake and reduces NADP^+^/NADPH ratios, leading to disulfidptosis and miscarriage. The cellular mechanisms are consistent with those in UM villous tissues and in Zn‐exposed mouse placental tissues. Knockdown of murine Slc7a11, Gata1, or Mettl1, or supplement with NADPH could effectively suppress mouse placental disulfidptosis and alleviate mouse miscarriage. Therefore, this study discovers novel pathogenesis and biological mechanisms of Zn exposure‐caused disulfidptosis and Zn exposure‐induced miscarriage.

### 4.3 M7G Modification is Associated with Miscarriage

M7G modification on mRNAs can regulate mRNA stability, and the abnormal M7G modification has been found to be associated with various diseases, such as tumors and Alzheimer's disease [67, 68]. Herein, we find for the first time that m7G modification levels on SLC7A11 mRNA are significantly higher in UM versus HC villous tissues, and its higher levels are positively associated with unexplained miscarriage. Moreover, in cellular assays, Zn exposure up‐regulates METTL1 expression levels and m7G modification levels on SLC7A11 mRNA, which up‐regulates SLC7A11 mRNA levels and ultimately induces disulfidptosis. In the ZnCl_2_‐exposed mouse model, Zn exposure also up‐regulates m7G modification levels on SLC7A11 mRNA and thus up‐regulates SLC7A11 mRNA levels, which results in placental disulfidptosis and induces mouse miscarriage. In addition to m7G modification, we also find that m6A modification is also closely associated with unexplained miscarriage. For example, m6A modification promotes the RNA stability of lnc‐HZ01, lnc‐HZ09, and lnc‐HZ14, and up‐regulates their levels and ultimately inhibits trophoblast proliferation (by lnc‐HZ01), migration/invasion (by lnc‐HZ09), and leading to trophoblast pyroptosis (by lnc‐HZ14), respectively, further inducing unexplained miscarriage [9, 13, 47]. Moreover, environmental BaP/BPDE exposure also up‐regulates m6A modification levels [9, 13, 47]. Therefore, RNA modifications are closely associated with unexplained miscarriage, and environmental toxicants might alter their modification levels. The relationship among environmental toxicants, the types and levels of RNA modification, and miscarriage should be extensively explored.

### 4.4 Miscarriage Treatment

Recently, increasing studies have uncovered the biological mechanisms of unexplained miscarriage [7, 8, 9, 10, 11, 12, 13, 14, 15, 16, 17, 18]. For examples, fructose‐1,6‐diphosphate has been reported to prevent miscarriage by inducing COX‐2 macrophage differentiation in the decidua [69]. Also, increasing succinate levels intravenously during the first trimester reduces the risk of recurrent miscarriage [70]. In our recent studies, we have found that supplement with GPX4 could suppress ferroptosis, recover angiogenesis, and alleviate BaP‐induced miscarriage in a mouse model [11]. Knockdown of either murine lnc‐hz06 or Ncoa4 could efficiently suppress ferroptosis and alleviate miscarriage in hypoxic mouse model [17]; knockdown of murine Ahr efficiently recovers homologous recombination repair in placental tissues and alleviates miscarriage in a BaP‐exposed mouse miscarriage model [7]; knockdown of Nlrp3 could reduce placental pyroptosis and alleviate BaP‐induced mouse miscarriage [13]; supplementing with murine SOX2 or ROCK1 could rescue migration/invasion and migrasome formation and alleviate polystyrene nanoplastics (PS‐NPs)‐induced miscarriage [15]; supplement with Bcl‐2 suppresses apoptosis in PS‐NPs‐exposed trophoblast cells and reduces apoptosis and alleviates miscarriage in PS‐NPs‐exposed pregnant mouse model [16]; and treatment with TTM (a cuproptosis inhibitor) suppresses placental cuproptosis and alleviates miscarriage in CuCl_2_‐exposed mouse model [43]. In this study, we find that knockdown of SLC7A11, GATA1, or METTL1, or supplement with NADPH could inhibit placental disulfidptosis and alleviate mouse miscarriage in ZnCl_2_‐exposed mouse model, providing new targets for effective treatment against unexplained miscarriage, enriching the therapeutic approach for unexplained miscarriage.

### 4.5 Limitations and Prospects

We collect women's characteristics from two case‐control groups. Other more confounders might also be considered; the possibility of misclassifications might not be completely avoided; and some factors (e.g., recall bias) might affect the reliability of the self‐reported data. Although Zn levels are higher in UM versus HC serum samples and higher Zn levels are associated with miscarriage, it cannot exclude that other toxicants (such as other metal ions or other toxicants) might also be associated with miscarriage or directly induce miscarriage. The transport pathway and organ enrichment of Zn in the human body remain unclear and need to be explored urgently.

In addition to the current study, environmental Zn exposure might also lead to other dysfunctions of human trophoblast cells and induce other adverse pregnancy outcomes. Moreover, Zn exposure might also cause other diseases rather than female reproduction outcomes. To detect Zn levels or other metal ion levels in serum or urine might be concerned in annual routine physical examination. The alluvial plot shows that Zn, GATA1, METTL1, and SLC7A11 are associated with various diseases (Figure S9P), including prenatal exposure delayed effects, nerve degeneration, learning disabilities, fatty liver, neoplasms, etc. [71, 72]. The association and causation among chemical‐gene‐phenotype‐disease should be further widely investigated. It has been reported that high SLC7A11‐induced disulfidptosis might be originated from NADPH depletion [32] and/or glucose starvation [31]. Then, we also studied the impact of Zn exposure on glucose metabolism. Zn exposure down‐regulated the mRNA and protein levels of glucose transporter‐1 (GLUT1) in human trophoblast cells (Figure S10A–C) and thus reduced the levels of glucose in trophoblast cells (Figure S10D). These findings suggested that, in addition to high SLC7A11‐induced NADPH depletion, Zn exposure might also result in glucose starvation, which further induces disulfidptosis. In addition to disulfidptosis, Zn exposure might also cause necroptosis or apoptosis, as shown in the cell viability assays (Figure 2B), which are deserved to be further explored. We have also analyzed another two top‐regulated pathways (such as PI3K‐AKT and p53) in the sequencing data (Figure S2H,J). Assays confirmed that Zn exposure up‐regulates the mRNA and protein levels of PI3K, AKT, and p53 (Figure S10E–K). These findings suggested that, in addition to disulfidptosis, other pathways such as PI3K‐AKT and p53 signaling, might also contribute to the Zn exposure‐induced miscarriage.

Moreover, in this study, we used short‐term and high‐dose Zn‐exposed animal and cell models, which might be higher than real environmental exposure doses. The long‐term and low‐dose of Zn exposure effects on women miscarriage should be further explored. In this study, we collected one‐spot serum and urine samples on the miscarriage day. It has been reported that different physiological or pathological statuses, including diet, inflammation, interactions with other trace metals, and tissue‐specific homeostasis might affect zinc levels in vivo [73, 74]. Therefore, it will be better to collect multiple serum or urine samples at different times per day to reduce the fluctuation of Zn in serum or urine samples. Moreover, the confounders such as diet, inflammation, interactions with other trace metals should also be collected and considered in further studies. Finally, the treatment against unexplained miscarriage should be further explored for better clinical translation.

## 5 Conclusion

Based on the epidemiological studies, mouse models, and cellular assays, we reach the consistent conclusion that Zn exposure causes disulfidptosis and thus induces unexplained miscarriage. Mechanistically, Zn exposure up‐regulates GATA1 expression levels, which promotes GATA1‐mediated METTL1 and SLC7A11 transcription. Moreover, Zn exposure also promotes METTL1‐mediated m7G modification on SLC7A11 mRNA and thus enhances SLC7A11 mRNA stability. Therefore, Zn exposure up‐regulates SLC7A11 mRNA levels at both transcription and post‐transcription levels, which further increase cystine intake and NADP^+^/NADPH ratios, resulting in disulfidptosis and inducing miscarriage. Knockdown of murine Gata1, Mettl1, or Slc7a11, or supplement with NADPH, effectively suppresses placental disulfidptosis and alleviates mouse miscarriage in the ZnCl_2_‐exposed mouse model. This study not only discovers new health risk effects of Zn exposure and novel pathogenesis and biological mechanisms of Zn exposure‐induced unexplained miscarriage but also provides potential targets for treatment against unexplained miscarriage.

## Author Contributions

H.Z., W.H., Y.S., Y.W., H.Y., D.Z., and G.G. conceived the project, designed the experiments, analyzed results, and wrote the manuscript. W.H., Y.S., Y.W., and H.Y. performed the majority of experiments. Y.L., X.Y., and Q. K. participated in partial experiments. X. Y. made stimulation and calculation. W. W., X.C. J.Z., Q.L., N.J, and L.Z. contributed data analysis and constructive comments. All authors read and provided suggestions during manuscript preparation.

## Conflicts of Interest

The authors declare no conflicts of interest.

## Supporting information




**Supporting File 1**: advs75261‐sup‐0001‐SuppMat.docx.

**Supporting File 2**: advs75261‐sup‐0002‐FigureS1‐10.pdf.

## Data Availability

All data and materials presented in this manuscript are available from the corresponding author (H. Zhang) upon a reasonable request under a completed Material Transfer Agreement. Any additional information required to reanalyze the data reported in this work paper is available from the corresponding author upon request.
