# Supplementary material for: Zinc Exposure Causes Disulfidptosis to Induce Miscarriage by Up‐Regulating GATA1/METTL1/SLC7A11 Axis
Source: Adv Sci (Weinh). 2026 May 7;13(42):e14513. doi: 10.1002/advs.202514513 (PMC13334944; doi:10.1002/advs.202514513)
Supplement: Supplementary file 1 — Supporting File 1: advs75261‐sup‐0001‐SuppMat.docx. [file ADVS-13-e14513-s002.docx]

**Zinc exposure causes disulfidptosis to induce miscarriage by up-regulating GATA1/METTL1/SLC7A11 axis**

Wenxin Huang^1,#^, Yi Sun^1,#^, Yanxin Wang^1,#^, Haijun Yan^1,#^, Xiaoping Yue^4^, Weidong Wu^5^, Xueyu Chen^2^, Jinfang Zhang^6^, Yanbing Lin^1^, Qiong Lei^7^, Nan Ji^8^, Shuaishuai Xing^1^, Liqin Zeng^6^, Qingzheng Kang^1^, Depeng Zhao^2,^*, Geng Guo^3,^*, and Huidong Zhang^1,^*

**Table S1. DNA sequences used for construction of pcDNA3.1 overexpression plasmids.**

| **Plasmid name** | **Gene name** | **Sequence region** |
| --- | --- | --- |
| pcDNA3.1-SLC7A11 | SLC7A11 | CDS region (NM_014331.4) |
| pcDNA3.1-GATA1 | GATA1 | CDS region (NM_002049.4) |
| pcDNA3.1-METTL1 | METTL1 | CDS region (NM_005371.6) |

**Table S2. RNA sequences used for RNA transfection.**

| **Name** | **Sense (5'-3')** | **Antisense (5'-3')** |
| --- | --- | --- |
| si1-SLC7A11 | CCCUGGAGUUAUGCAGCUAAUTT | AUUAGCUGCAUAACUCCAGGGTT |
| si2-SLC7A11 | CCUGCGUAUUAUCUCUUUAUUTT | AAUAAAGAGAUAAUACGCAGGTT |
| si1-GATA1 | UGUACCCAUUGCUCAACUGUATT | UACAGUUGAGCAAUGGGUACATT |
| si2-GATA1 | UACAGUUGAGCAAUGGGUACATT | AAUACCAUCCUUCCGCAUGGUTT |
| si1-METTL1 | GAUGACCCAAAGGAUAAGAAATT | UUUCUUAUCCUUUGGGUCAUCTT |
| si2-METTL1 | CGACUGGAUGUGCACUCAUUUTT | AAAUGAGUGCACAUCCAGUCGTT |
| NC | UUCUCCGAACGUGUCACGUTT | ACGUGACACGUUCGGAGAATT |

**Table S3. Primer sequences used for RT-qPCR.**

| **Species** | **Gene** | **Forward (5'-3')** | **Reverse (5'-3')** |
| --- | --- | --- | --- |
| Human | SLC7A11 | TCTCCAAAGGAGGTTACCTGC | AGACTCCCCTCAGTAAAGTGAC |
|  | m7G-SLC7A11 | GTTTGTAATGATAGGGCGGC | CTTCACAGCGATCTAATTACTACTC |
|  | ChIP-SLC7A11 | GACCAGCCCTATTGTCCTCC | GCTGGGCTAAAACATTGAAGG |
|  | Biotin-SLC7A11 | Biotin-AGAAACACACTGGGGAG | AATGAGATTTGCTGGGCT |
|  | METTL1 | GGCAACGTGCTCACTCCAA | CACAGCCTATGTCTGCAAACT |
|  | ChIP-METTL1 | CGAGTGCTTTCAGTTTGAGGT | TTTGTAGGAAGCGGGGTAGG |
|  | Biotin-METTL1 | Biotin-CGAGTGCTTTCAGTTTGAG | ACCTCAGGTGATCCATCT |
|  | GATA1 | CTGTCCCCAATAGTGCTTATGG | GAATAGGCTGCTGAATTGAGGG |
|  | GAPDH | TGTGTCCGTCGTGGATCTGA | GCAGCTGTGACACACAGTA |
| Mouse | Slc7a11 | AGGGCATACTCCAGAACACG | GGACCAAAGACCTCCAGAATG |
|  | Mellt1 | GAGCCATGATGATCCAAAGGATG | GTGCCACTAACAAGCCACCAT |
|  | Gata1 | TGTCCTCACCATCAGATTCCA | TCCCTCCATACTGTTGAGCAG |
|  | Gapdh | TGGCCTTCCGTGTTCCTAC | GAGTTGCTGTTGAAGTCGCA |

**Table S4. The potential confounders in HC and UM women in Group 1.**

| **Characteristic** | **HC*^b^* (n = 50)** | **UM*^e^* (n = 50)** | **P-value*^f^*** |
| --- | --- | --- | --- |
| **Age** | 27.4 ± 2.28*^c^* | 27.8 ± 1.88 | 0.347 |
| **Gestational week** | 7.08 ± 0.61 | 6.91 ± 0.47 | 0.138 |
| **BMI*^a^*** | 23.9 ± 2.23 | 23.4 ± 2.05 | 0.246 |
| **Anemia** |  |  | 0.2945 |
| No | 4*^d^* | 44 |  |
| Yes | 3 | 6 |  |
| **Education** |  |  | 0.509 |
| < High school | 16 | 13 |  |
| ≥ High school | 34 | 37 |  |
| **Residence** |  |  | 0.547 |
| Rural | 21 | 24 |  |
| Urban | 29 | 26 |  |
| **Household income, RMB Yuan/month** | |  | 0.054 |
| < 5000 | 7 | 15 |  |
| ≥ 5000 | 43 | 35 |  |
| **Smoking in the past 3 months** | |  | >0.999 |
| No | 32 | 32 |  |
| Yes | 18 | 18 |  |
| **Drinking in the past 3 months** | |  | 0.648 |
| No | 38 | 36 |  |
| Yes | 12 | 14 |  |

*^a^*BMI: Body Mass Index.

*^b^*HC: healthy control group.

*^c^*mean ± standard deviation (n = 50 in each UM or HC group).

*^d^*number of women

*^e^*UM: unexplained miscarriage group.

*^f^*Student's t-test or Chi-square test.

**Table S5. The potential confounders in HC and UM women in Group 2.**

| **Characteristic** | **HC*^b^* (n = 40)** | **UM*^e^* (n = 40)** | **P-value*^f^*** |
| --- | --- | --- | --- |
| **Age** | 27.5 ± 1.72*^c^* | 27.7 ± 1.48 | 0.454 |
| **Gestational week** | 7.19 ± 0.57 | 6.95 ± 0.62 | 0.068 |
| **BMI*^a^*** | 23.7 ± 3.01 | 22.6 ± 2.93 | 0.098 |
| **Anemia** |  |  | 0.235 |
| No | 38*^d^* | 35 |  |
| Yes | 2 | 5 |  |
| **Education** |  |  | 0.237 |
| < High school | 16*^i^* | 11 |  |
| ≥ High school | 24 | 29 |  |
| **Residence** |  |  | 0.361 |
| Rural | 18 | 14 |  |
| Urban | 22 | 26 |  |
| **Household income, RMB** | |  | 0.813 |
| < 5000 | 13 | 14 |  |
| ≥ 5000 | 27 | 26 |  |
| **Smoking in the past 3 months** | |  | 0.496 |
| No | 22 | 25 |  |
| Yes | 18 | 15 |  |
| **Drinking in the past 3 months** | |  | 0.805 |
| No | 29 | 28 |  |
| Yes | 11 | 12 |  |

*^a^*BMI: Body Mass Index.

*^b^*HC: healthy control group.

*^c^*mean ± standard deviation (n = 50 in each UM or HC group).

*^d^*number of women.

*^e^*UM: unexplained miscarriage group.

*^f^*Student's t-test or Chi-square test.

**Table S6 The calculation of the exposure doses of Zn in ZnCl_2_-exposed mouse model.**

| **Group** | **Human real exposure dose** | Mouse exposure dose | | **-fold REED**^e^ |
| --- | --- | --- | --- | --- |
|  |  | **Zn**  **(mg/kg/day)** | **ZnCl_2_^c^**  **(mg/kg/day)** |  |
| 1 | **Control, n**o exposure | 0 | 0 | 0 |
| 2 | Chongqing residents daily Zn intake in real environments (0.243 mg/L/day)^22^ | 3^a^ | 6 | 1 |
| 3 | Mice with 28 mg/kg ZnCl_2_ suppressed the functions of hepatic^45^ | 16 | 33^d^ | 5.5 |
| 4 | Oral exposure dose with adverse outcomes (300 mg/70 kg/d Zn)^22^ | 51.6^b^ | 107 | 17.8 |

^a^Mouse real Zn exposure dose = human real Zn exposure dose (0.243 mg/kg/day) × 12 (body surface area coefficient) ≈ 3 mg/kg/day.

^b^Mouse Zn exposure dose = human oral Zn exposure dose with adverse outcomes (300 mg / 70 kg (human average weight) / 1 day ≈ 4.3 mg/kg/day) × 12 (body surface area coefficient) = 51.6 mg/kg/day.

^c^ZnCl_2_ exposure dose = Zn exposure dose / 65.38 g/mol (Zn atomic weight) × 136.3 g/mol (ZnCl_2_ molecular weight).

^d^Mouse ZnCl_2_ exposure dose = 1 / 10 × LD50 (329 mg/kg/day ZnCl_2_) ≈ 33 mg/kg/day.

^e^REED meant really environmental exposure dose. The fold number was calculated by mouse ZnCl_2_ exposure dose in each group / 6 mg/kg/d.

**Table S7. Multivariate logistic regression analysis of the characteristic and miscarriage***^a^***.**

| **Groups** | **Characteristic** | **Unadjusted OR (95% CI)** | **Adjusted OR (95% CI)** |
| --- | --- | --- | --- |
| Group 1 | Zn levels in serum | 4.235 (2.378 - 7.543) | 4.707 (2.394 - 9.256) |
|  | Zn levels in urine | 1.769 (1.129 - 2.771) | 1.907 (1.128 - 3.224) |
|  | Cystine levels | 1.883 (1.329 - 2.668) | 1.936 (1.330 - 2.819) |
|  | NADP^+^/NADPH ratio | 44.984 (8.863 - 228.324) | 86.558 (13.607 - 550.616) |
|  | SLC7A11 mRNA levels | 1.134 (1.061 - 1.213) | 1.188 (1.095 - 1.289) |
|  | GATA1 mRNA levels | 1.338 (1.122 - 1.597) | 1.580 (1.262 - 1.979) |
|  | METTL1 mRNA levels | 1.299 (1.129 - 1.495) | 1.602 (1.237 - 2.074) |
| Group 2 | Zn levels in serum | 1.486 (1.241 - 1.778) | 1.792 (1.363 - 2.356) |
|  | Zn levels in urine | 1.446 (1.147 - 1.824) | 1.490 (1.155 - 1.922) |
|  | Cystine levels | 2.492 (1.633 - 3.803) | 2.723 (1.698 - 4.369) |
|  | NADP^+^/NADPH ratio | 23.151 (4.361 - 122.906) | 34.583 (4.856 - 246.280) |
|  | SLC7A11 mRNA levels | 1.318 (1.151 - 1.510) | 1.664 (1.316 - 2.103) |
|  | GATA1 mRNA levels | 1.313 (1.133 - 1.522) | 1.340 (1.139 - 1.576) |
|  | METTL1 mRNA levels | 1.124 (1.045 - 1.210) | 1.134 (1.047 - 1.228) |

*^a^*Adjusted for age, BMI, education, household income, smoking, and drinking.

**Table S8. Sequence conservation analysis of the mRNA and protein in human and in other species^a^**

|  |  |  | **Rhesus** | **Mouse** | **Dog** | **Elephant** |
| --- | --- | --- | --- | --- | --- | --- |
| **mRNA** | SLC7A11 | Per.Ident*^b^* | 95.79% | 85.02% | 92.41% | 83.31% |
|  | GATA1 | Per.Ident | 96.86% | 83.05% | 88.90% | 88.00% |
|  | METTL1 | Per.Ident | 96.56% | 87.17% | 91.4% | 85.68% |
|  | FLNA | Per.Ident | 96.52% | 87.32% | 89.97% | 89.94% |
|  | MYH9 | Per.Ident | 96.78% | 90.37% | 89.74% | 92.48% |
|  | Drebrin | Per.Ident | 96.38% | 82.55% | 85.69% | 85.79% |
|  | F-actin | Per.Ident | 97.36% | 90.36% | 92.35% | 89.28% |
| **Protein** | SLC7A11 | Per.Ident*^b^* | 98.00% | 89.31% | 91.62% | 93.41% |
|  | GATA1 | Per.Ident | 93.62% | 85.96% | 95.15% | 97.82% |
|  | METTL1 | Per.Ident | 98.55% | 90.30% | 90.58% | 76.36% |
|  | FLNA | Per.Ident | 96.52% | 97.32% | 97.6% | 97.86% |
|  | MYH9 | Per.Ident | 99.80% | 97.09% | 98.27% | 96.22% |
|  | Drebrin | Per.Ident | 32.07% | 88.44% | 33.25% | 34.94% |
|  | F-actin | Per.Ident | 100.0% | 100.0% | 100.0% | 97.60% |

*^a^*Sequence conservation of SLC7A11, GATA1, METTL1, FLNA, MYH9, Drebrin, and F-actin was explored using NCBI Blast (<https://blast.ncbi.nlm.nih.gov/Blast.cgi>).

*^b^*Per.Ident means the ratio of the number of the matched bases to that of the total bases.

**Table S9. The promoter sequence of SLC7A11 or METTL1.**

| **Name** | **Sense (5'-3')** |
| --- | --- |
| The promoter sequence of SLC7A11 | TCCTTATCTCA |
| The promoter sequence of METTL1 | TTATAATCTAA |

**Table S10. The binding energy between GATA1 and SLC7A11 promoter region.**

| Key residue | Total Energy (kcal/mol) | | Δ Energy (kcal/mol) |
| --- | --- | --- | --- |
|  | NaCl-GATA1-SLC7A11 | ZnCl_2_-GATA1-SLC7A11 |  |
| Total | -205.46 | -214.97 | -9.51 |
| LEU:248 | -0.39 | -1.61 | -1.21 |
| TYR:285 | -1.98 | -2.83 | -0.85 |
| LEU:288 | -2.87 | -2.46 | 0.42 |
| HSD:289 | -1.59 | -1.71 | -0.11 |
| PRO:294 | -0.16 | -0.26 | -0.10 |
| MET:297 | -1.47 | -2.41 | -0.94 |
| ARG:298 | -1.18 | -1.35 | -0.16 |
| LYS:299 | -2.56 | -3.56 | -1.00 |
| ILE:302 | -1.72 | -1.59 | 0.13 |
| THR:304 | -2.30 | -1.72 | 0.58 |
| ARG:307 | -10.12 | -10.32 | -0.20 |
| LYS:308 | -1.83 | -2.48 | -0.65 |
| SER:310 | -1.17 | -0.71 | 0.46 |
| GLY:311 | -1.54 | -0.12 | 1.42 |
| LYS:312 | -1.99 | -0.92 | 1.08 |
| GLY:313 | -1.12 | -1.99 | -0.87 |
| LYS:314 | -2.05 | -4.39 | -2.34 |
| LYS:315 | -2.05 | -5.03 | -2.98 |
| LYS:316 | -1.52 | -1.63 | -0.12 |
| ARG:317 | -4.31 | -5.85 | -1.54 |

**Table S11. The binding energy between GATA1 and METTL1 promoter region.**

| Key residue | Total Energy (kcal/mol) | | Δ Energy (kcal/mol) |
| --- | --- | --- | --- |
|  | NaCl-GATA1-METTL1 | ZnCl_2_-GATA1-METTL1 |  |
| Total | -202.84 | -210.98 | -8.14 |
| ARG:239 | -0.30 | -4.59 | -4.29 |
| PRO:240 | -0.04 | -1.26 | -1.22 |
| LEU:241 | -0.03 | -0.31 | -0.28 |
| ILE:242 | -0.30 | -2.31 | -2.00 |
| ARG:243 | -0.38 | -5.26 | -4.88 |
| PRO:244 | -0.37 | -0.24 | 0.13 |
| LYS:245 | -2.80 | -1.23 | 1.56 |
| LYS:246 | -0.83 | -1.20 | -0.37 |
| LEU:248 | -0.95 | -0.45 | 0.50 |
| ILE:249 | -0.91 | -0.47 | 0.45 |
| ARG:270 | -6.08 | -5.92 | 0.16 |
| ARG:271 | -3.17 | -3.34 | -0.17 |
| ASN:280 | -2.97 | -3.09 | -0.12 |
| LEU:284 | -3.82 | -3.93 | -0.11 |
| TYR:285 | -1.36 | -0.84 | 0.51 |
| LYS:287 | -2.13 | -2.39 | -0.25 |
| LEU:288 | -0.97 | -0.79 | 0.18 |
| ARG:305 | -11.04 | -12.17 | -1.13 |
| LYS:312 | -3.45 | -6.28 | -2.83 |
| LYS:315 | -1.98 | -2.25 | -0.27 |
