# Supplementary material for: Zinc Exposure Causes Disulfidptosis to Induce Miscarriage by Up‐Regulating GATA1/METTL1/SLC7A11 Axis
Source: Adv Sci (Weinh). 2026 May 7;13(42):e14513. doi: 10.1002/advs.202514513 (PMC13334944; doi:10.1002/advs.202514513)
Supplement: Supplementary file 2 — Supporting File 2: advs75261‐sup‐0002‐FigureS1‐10.pdf. [file ADVS-13-e14513-s001.pdf]

Fig.S1-1

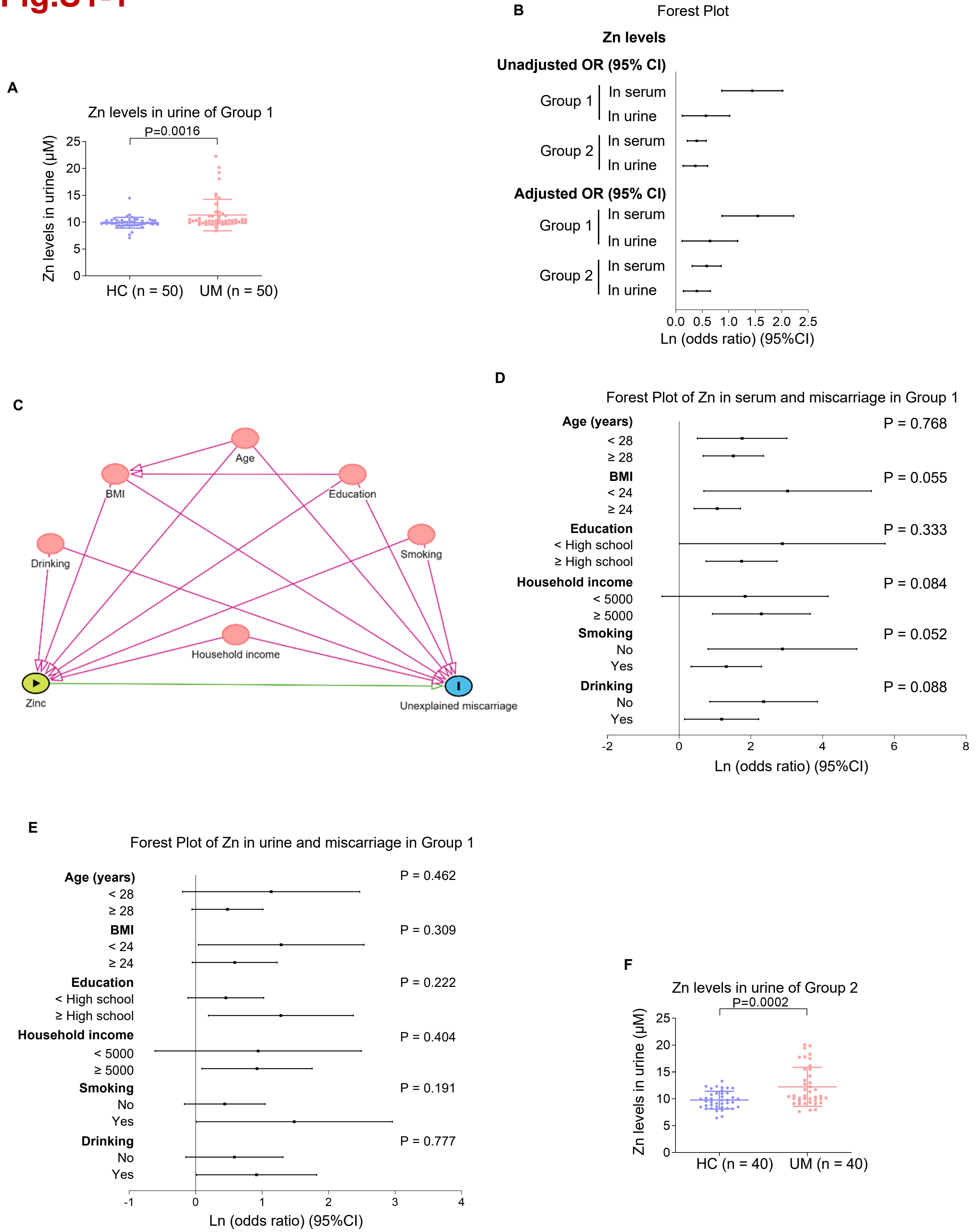
A
Zn levels in urine of Group 1
P=0.0016
Zn levels in urine (μM)
25
20
15
10
5
0
HC (n = 50)
UM (n = 50)
B
Forest Plot
Zn levels
Unadjusted OR (95% CI)
Group 1
In serum
In urine
Group 2
In serum
In urine
Adjusted OR (95% CI)
Group 1
In serum
In urine
Group 2
In serum
In urine
0.0 0.5 1.0 1.5 2.0 2.5
Ln (odds ratio) (95%CI)
C
Age
BMI
Education
Drinking
Smoking
Household income
Zinc
Unexplained miscarriage
D
Forest Plot of Zn in serum and miscarriage in Group 1
Age (years)
< 28
≥ 28
P = 0.768
BMI
< 24
≥ 24
P = 0.055
Education
< High school
≥ High school
P = 0.333
Household income
< 5000
≥ 5000
P = 0.084
Smoking
No
Yes
P = 0.052
Drinking
No
Yes
P = 0.088
-2 0 2 4 6 8
Ln (odds ratio) (95%CI)
E
Forest Plot of Zn in urine and miscarriage in Group 1
Age (years)
< 28
≥ 28
P = 0.462
BMI
< 24
≥ 24
P = 0.309
Education
< High school
≥ High school
P = 0.222
Household income
< 5000
≥ 5000
P = 0.404
Smoking
No
Yes
P = 0.191
Drinking
No
Yes
P = 0.777
-1 0 1 2 3 4
Ln (odds ratio) (95%CI)
F
Zn levels in urine of Group 2
P=0.0002
Zn levels in urine (μM)
25
20
15
10
5
0
HC (n = 40)
UM (n = 40)

Fig.S1-2

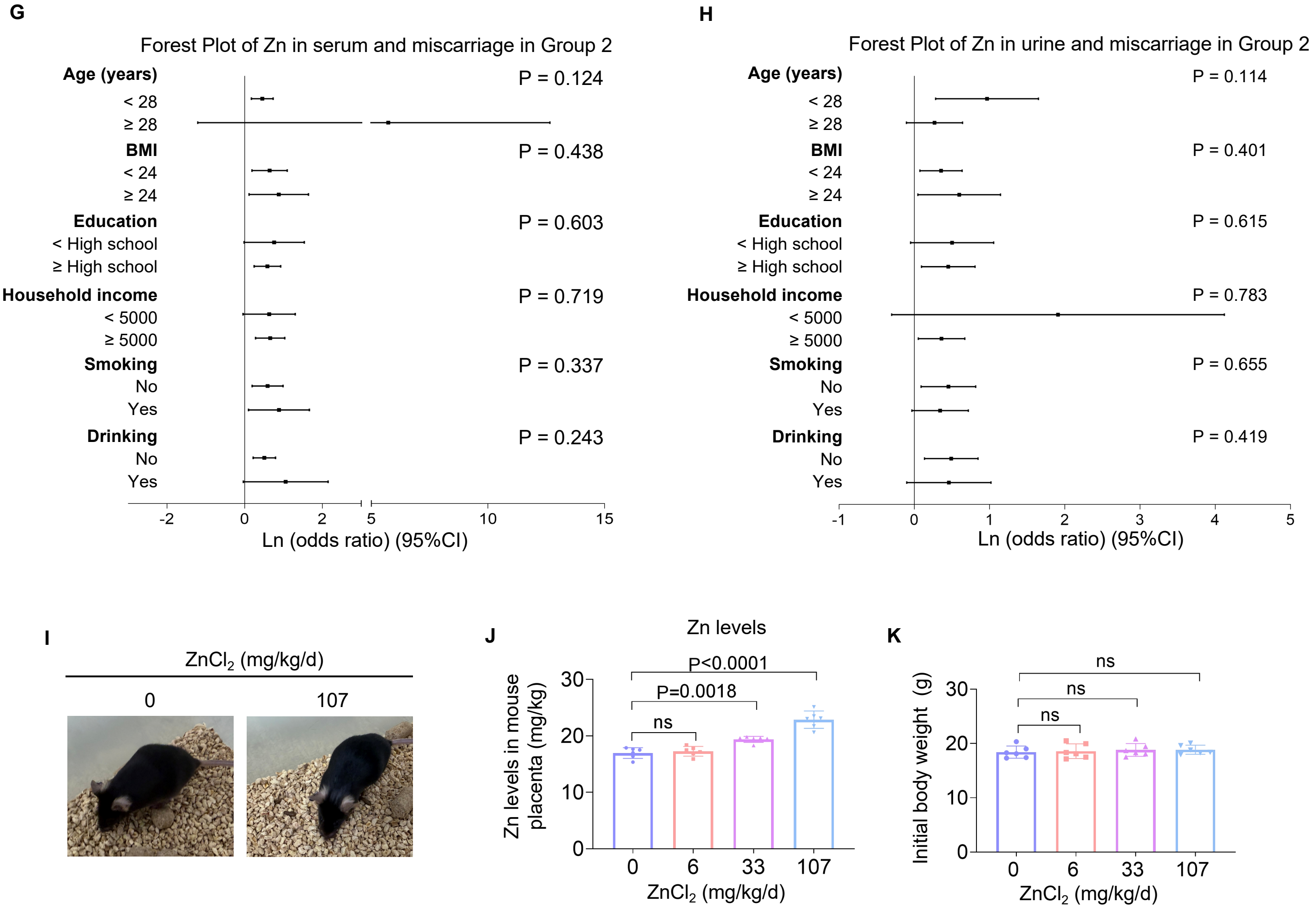
G
Forest Plot of Zn in serum and miscarriage in Group 2
Age (years)
< 28
≥ 28
P = 0.124
BMI
< 24
≥ 24
P = 0.438
Education
< High school
≥ High school
P = 0.603
Household income
< 5000
≥ 5000
P = 0.719
Smoking
No
Yes
P = 0.337
Drinking
No
Yes
P = 0.243
-2 0 2 5 10 15
Ln (odds ratio) (95%CI)
H
Forest Plot of Zn in urine and miscarriage in Group 2
Age (years)
< 28
≥ 28
P = 0.114
BMI
< 24
≥ 24
P = 0.401
Education
< High school
≥ High school
P = 0.615
Household income
< 5000
≥ 5000
P = 0.783
Smoking
No
Yes
P = 0.655
Drinking
No
Yes
P = 0.419
-1 0 1 2 3 4 5
Ln (odds ratio) (95%CI)
I
$ZnCl_2$ (mg/kg/d)
0
107
J
Zn levels
Zn levels in mouse placenta (mg/kg)
P<0.0001
P=0.0018
ns
0 10 20 30
0 6 33 107
$ZnCl_2$ (mg/kg/d)
K
Initial body weight (g)
ns
ns
ns
0 10 20 30
0 6 33 107
$ZnCl_2$ (mg/kg/d)

Fig.S2-1

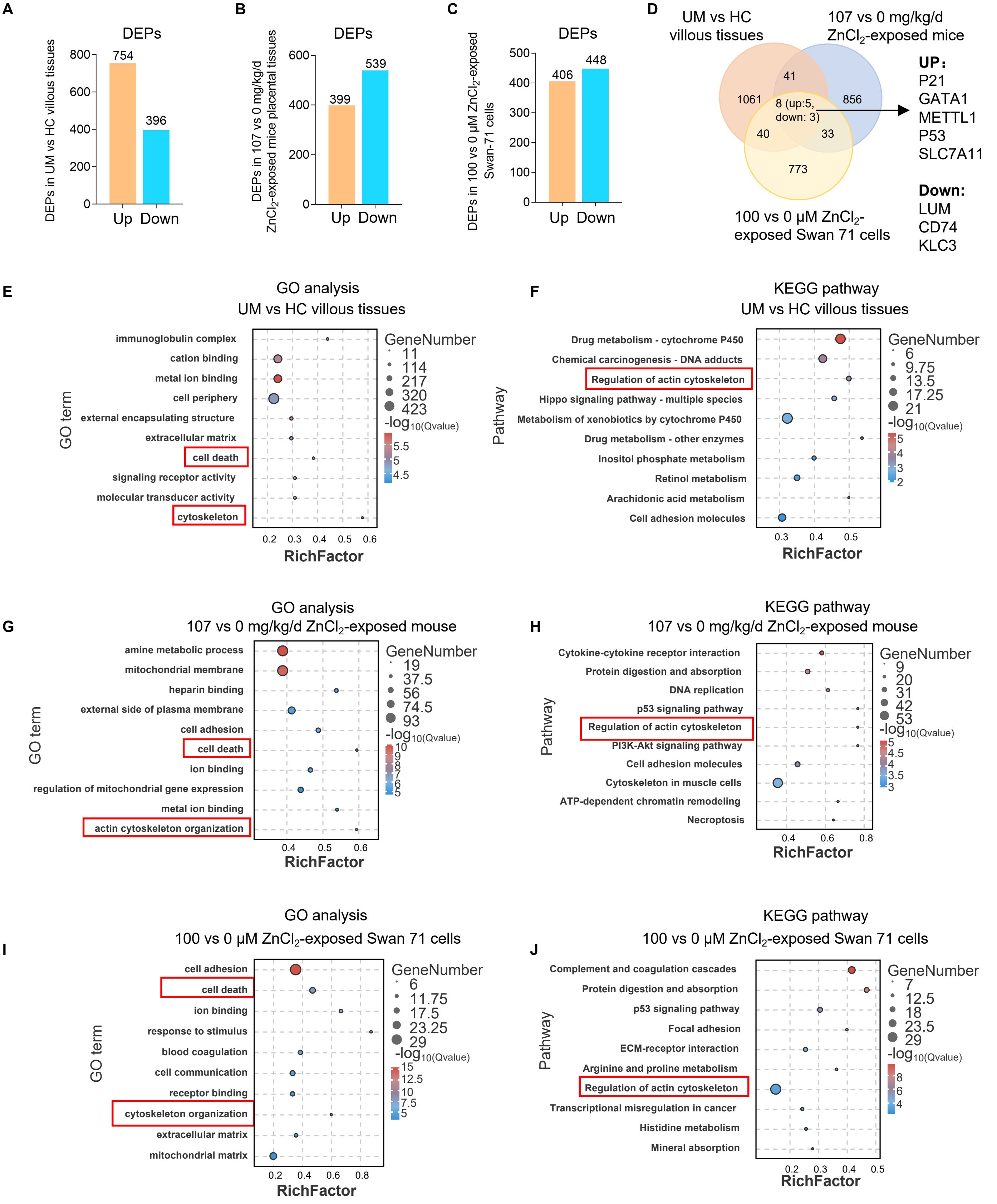
A
DEPs
DEPs in UM vs HC villous tissues
800
600
400
200
0
754
396
Up
Down
B
DEPs
DEPs in 107 vs 0 mg/kg/d ZnCl2-exposed mice placental tissues
600
400
200
0
399
539
Up
Down
C
DEPs
DEPs in 100 vs 0 μM ZnCl2-exposed Swan-71 cells
500
400
300
200
100
0
406
448
Up
Down
D
UM vs HC villous tissues
107 vs 0 mg/kg/d ZnCl2-exposed mice
100 vs 0 μM ZnCl2-exposed Swan 71 cells
1061
41
856
8 (up:5, down: 3)
40
33
773
UP:
P21
GATA1
METTL1
P53
SLC7A11
Down:
LUM
CD74
KLC3
E
GO analysis
UM vs HC villous tissues
GO term
immunoglobulin complex
cation binding
metal ion binding
cell periphery
external encapsulating structure
extracellular matrix
cell death
signaling receptor activity
molecular transducer activity
cytoskeleton
RichFactor
0.2
0.3
0.4
0.5
0.6
GeneNumber
11
114
217
320
423
-log10(Qvalue)
5.5
5
4.5
F
KEGG pathway
UM vs HC villous tissues
Pathway
Drug metabolism - cytochrome P450
Chemical carcinogenesis - DNA adducts
Regulation of actin cytoskeleton
Hippo signaling pathway - multiple species
Metabolism of xenobiotics by cytochrome P450
Drug metabolism - other enzymes
Inositol phosphate metabolism
Retinol metabolism
Arachidonic acid metabolism
Cell adhesion molecules
RichFactor
0.3
0.4
0.5
GeneNumber
6
9.75
13.5
17.25
21
-log10(Qvalue)
5
4
3
2
G
GO analysis
107 vs 0 mg/kg/d ZnCl2-exposed mouse
GO term
amine metabolic process
mitochondrial membrane
heparin binding
external side of plasma membrane
cell adhesion
cell death
ion binding
regulation of mitochondrial gene expression
metal ion binding
actin cytoskeleton organization
RichFactor
0.4
0.5
0.6
GeneNumber
19
37.5
56
74.5
93
-log10(Qvalue)
10
9
8
7
6
5
H
KEGG pathway
107 vs 0 mg/kg/d ZnCl2-exposed mouse
Pathway
Cytokine-cytokine receptor interaction
Protein digestion and absorption
DNA replication
p53 signaling pathway
Regulation of actin cytoskeleton
PI3K-Akt signaling pathway
Cell adhesion molecules
Cytoskeleton in muscle cells
ATP-dependent chromatin remodeling
Necroptosis
RichFactor
0.4
0.6
0.8
GeneNumber
9
20
31
42
53
-log10(Qvalue)
5
4.5
4
3.5
3
I
GO analysis
100 vs 0 μM ZnCl2-exposed Swan 71 cells
GO term
cell adhesion
cell death
ion binding
response to stimulus
blood coagulation
cell communication
receptor binding
cytoskeleton organization
extracellular matrix
mitochondrial matrix
RichFactor
0.2
0.4
0.6
0.8
GeneNumber
6
11.75
17.5
23.25
29
-log10(Qvalue)
15
12.5
10
7.5
5
J
KEGG pathway
100 vs 0 μM ZnCl2-exposed Swan 71 cells
Pathway
Complement and coagulation cascades
Protein digestion and absorption
p53 signaling pathway
Focal adhesion
ECM-receptor interaction
Arginine and proline metabolism
Regulation of actin cytoskeleton
Transcriptional misregulation in cancer
Histidine metabolism
Mineral absorption
RichFactor
0.2
0.3
0.4
0.5
GeneNumber
7
12.5
18
23.5
29
-log10(Qvalue)
8
6
4

Fig.S2-2

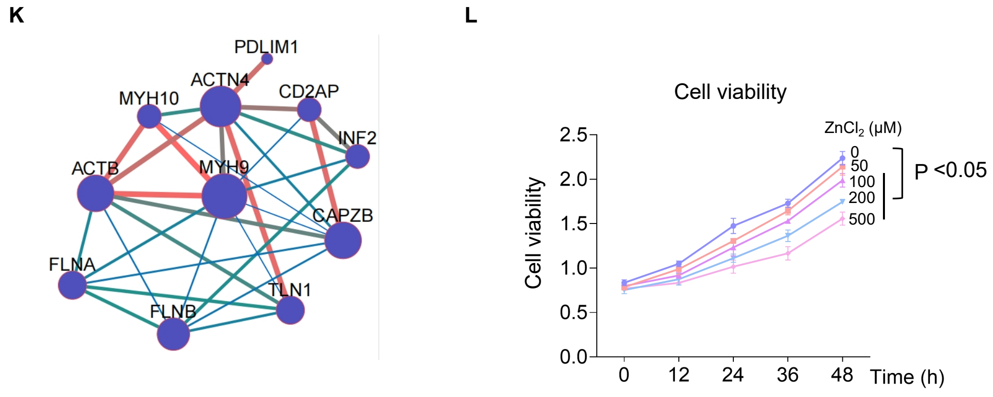
K
PDLIM1
ACTN4
MYH10
CD2AP
INF2
ACTB
MYH9
CAPZB
FLNA
TLN1
FLNB
L
Cell viability
ZnCl2 (μM)
0
50
100
200
500
P <0.05
Cell viability
2.5
2.0
1.5
1.0
0.5
0.0
0
12
24
36
48
Time (h)

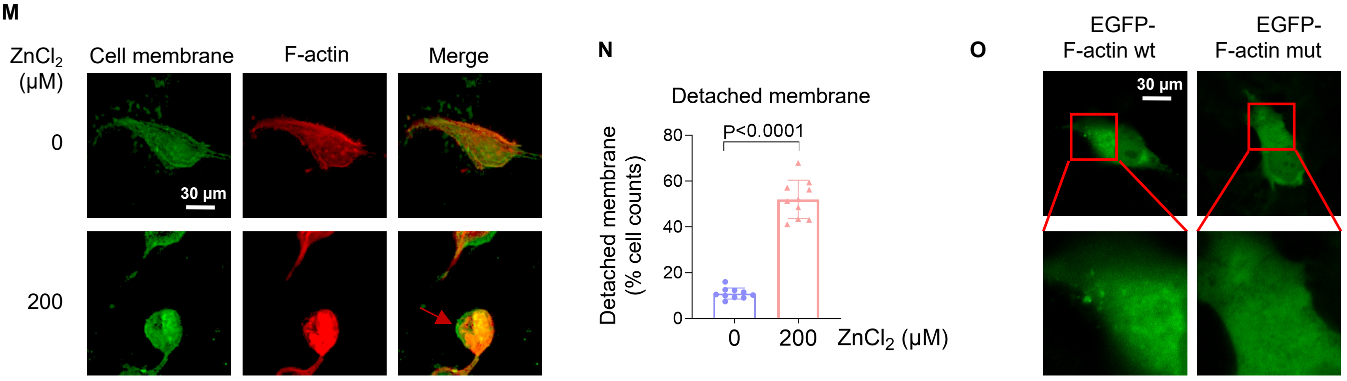
M
ZnCl2 (μM)
Cell membrane
F-actin
Merge
0
30 μm
200
N
Detached membrane
P<0.0001
Detached membrane (% cell counts)
80
60
40
20
0
0
200
ZnCl2 (μM)
O
EGFP-F-actin wt
EGFP-F-actin mut
30 μm

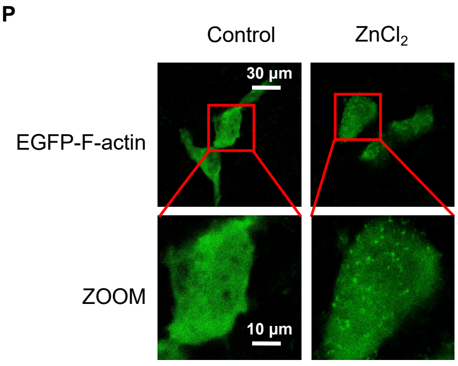
P
Control
ZnCl2
EGFP-F-actin
30 μm
ZOOM
10 μm

Fig. S3-1

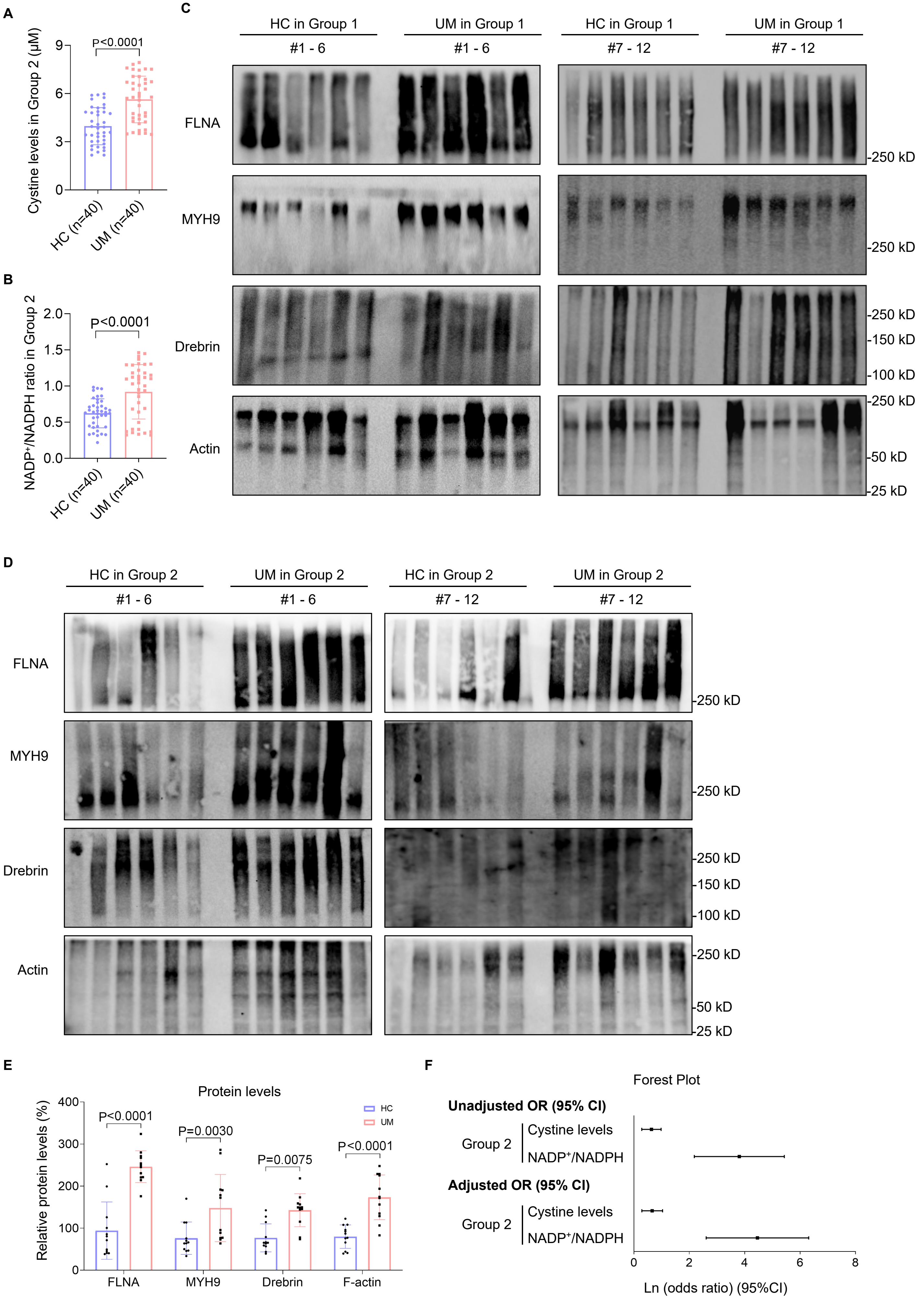
A
Cystine levels in Group 2 (μM)
P<0.0001
HC (n=40)
UM (n=40)
B
NADP+/NADPH ratio in Group 2
P<0.0001
HC (n=40)
UM (n=40)
C
HC in Group 1
#1 - 6
UM in Group 1
#1 - 6
HC in Group 1
#7 - 12
UM in Group 1
#7 - 12
FLNA
MYH9
Drebrin
Actin
250 kD
150 kD
100 kD
50 kD
25 kD
D
HC in Group 2
#1 - 6
UM in Group 2
#1 - 6
HC in Group 2
#7 - 12
UM in Group 2
#7 - 12
FLNA
MYH9
Drebrin
Actin
250 kD
150 kD
100 kD
50 kD
25 kD
E
Protein levels
Relative protein levels (%)
HC
UM
P<0.0001
P=0.0030
P=0.0075
P<0.0001
FLNA
MYH9
Drebrin
F-actin
F
Forest Plot
Unadjusted OR (95% CI)
Group 2
Cystine levels
NADP+/NADPH
Adjusted OR (95% CI)
Group 2
Cystine levels
NADP+/NADPH
Ln (odds ratio) (95%CI)

Fig.S3-2

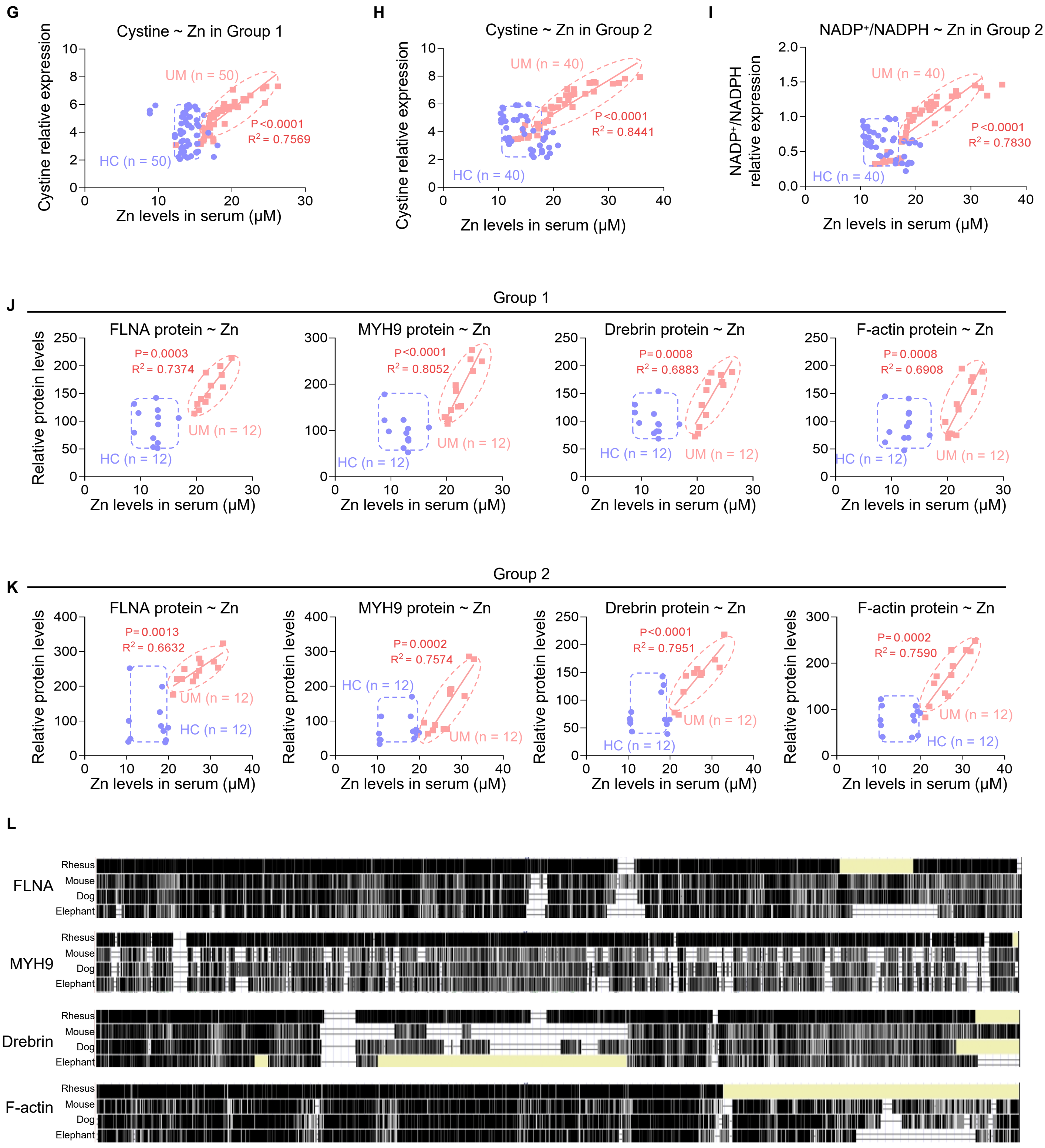
G
Cystine ~ Zn in Group 1
UM (n = 50)
P<0.0001
R2 = 0.7569
HC (n = 50)
Cystine relative expression
Zn levels in serum (μM)
H
Cystine ~ Zn in Group 2
UM (n = 40)
P<0.0001
R2 = 0.8441
HC (n = 40)
Cystine relative expression
Zn levels in serum (μM)
I
NADP+/NADPH ~ Zn in Group 2
UM (n = 40)
P<0.0001
R2 = 0.7830
HC (n = 40)
NADP+/NADPH relative expression
Zn levels in serum (μM)
J
Group 1
FLNA protein ~ Zn
P=0.0003
R2 = 0.7374
MYH9 protein ~ Zn
P<0.0001
R2 = 0.8052
Drebrin protein ~ Zn
P=0.0008
R2 = 0.6883
F-actin protein ~ Zn
P=0.0008
R2 = 0.6908
UM (n = 12)
HC (n = 12)
Relative protein levels
Zn levels in serum (μM)
K
Group 2
FLNA protein ~ Zn
P=0.0013
R2 = 0.6632
MYH9 protein ~ Zn
P=0.0002
R2 = 0.7574
Drebrin protein ~ Zn
P<0.0001
R2 = 0.7951
F-actin protein ~ Zn
P=0.0002
R2 = 0.7590
UM (n = 12)
HC (n = 12)
Relative protein levels
Zn levels in serum (μM)
L
FLNA
MYH9
Drebrin
F-actin
Rhesus
Mouse
Dog
Elephant

Fig.S3-3

M

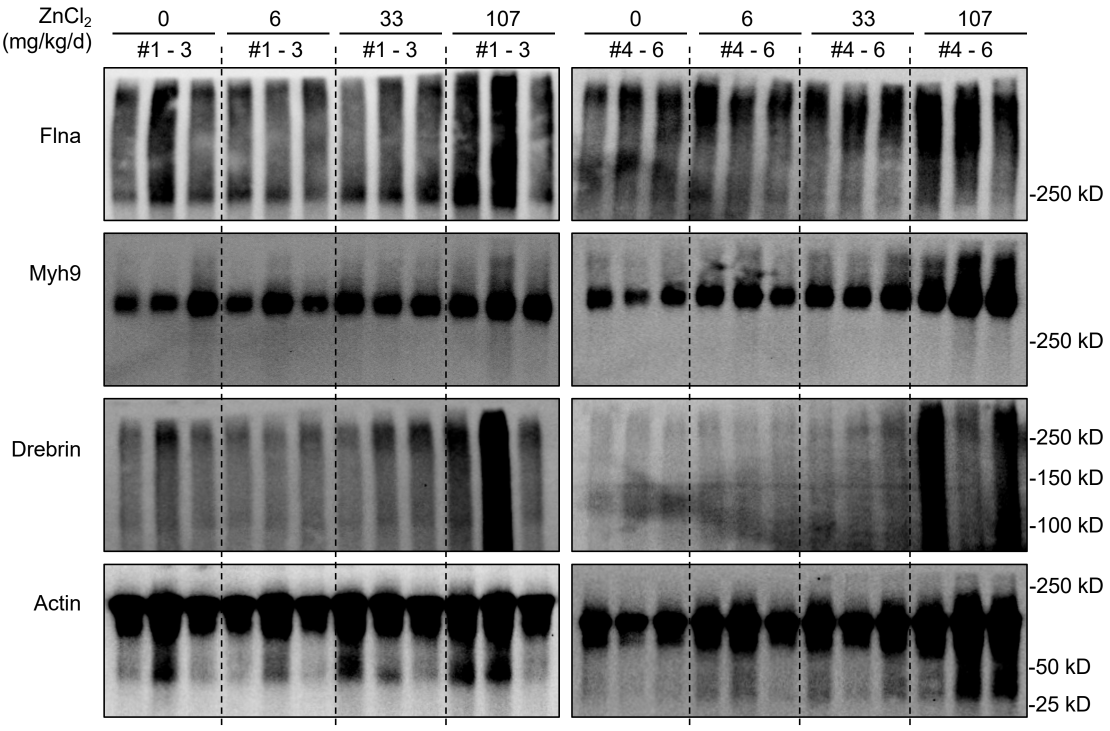
ZnCl$_2$ (mg/kg/d)
0
6
33
107
#1 - 3
0
6
33
107
#4 - 6
Flna
Myh9
Drebrin
Actin
-250 kD
-150 kD
-100 kD
-50 kD
-25 kD

N

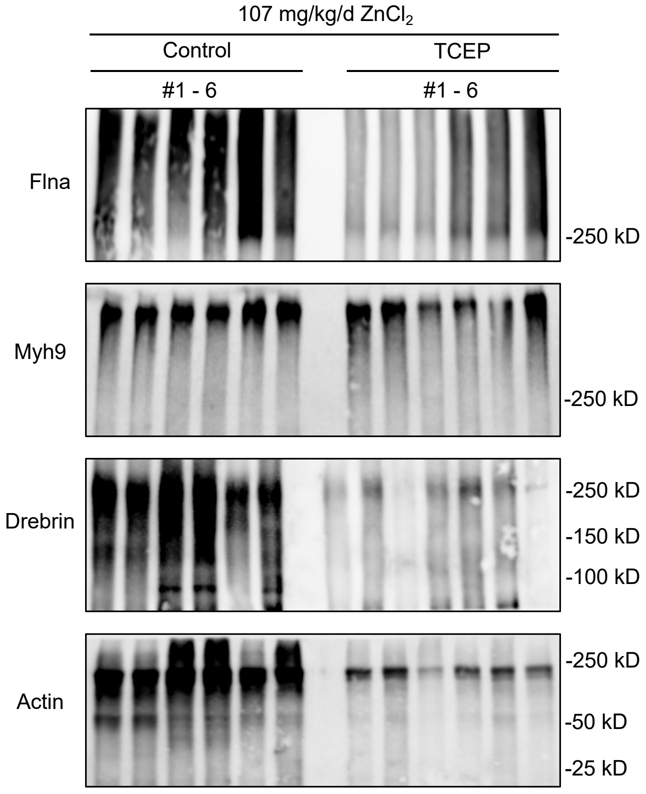
107 mg/kg/d ZnCl$_2$
Control
TCEP
#1 - 6
Flna
Myh9
Drebrin
Actin
-250 kD
-150 kD
-100 kD
-50 kD
-25 kD

Fig.S4-1

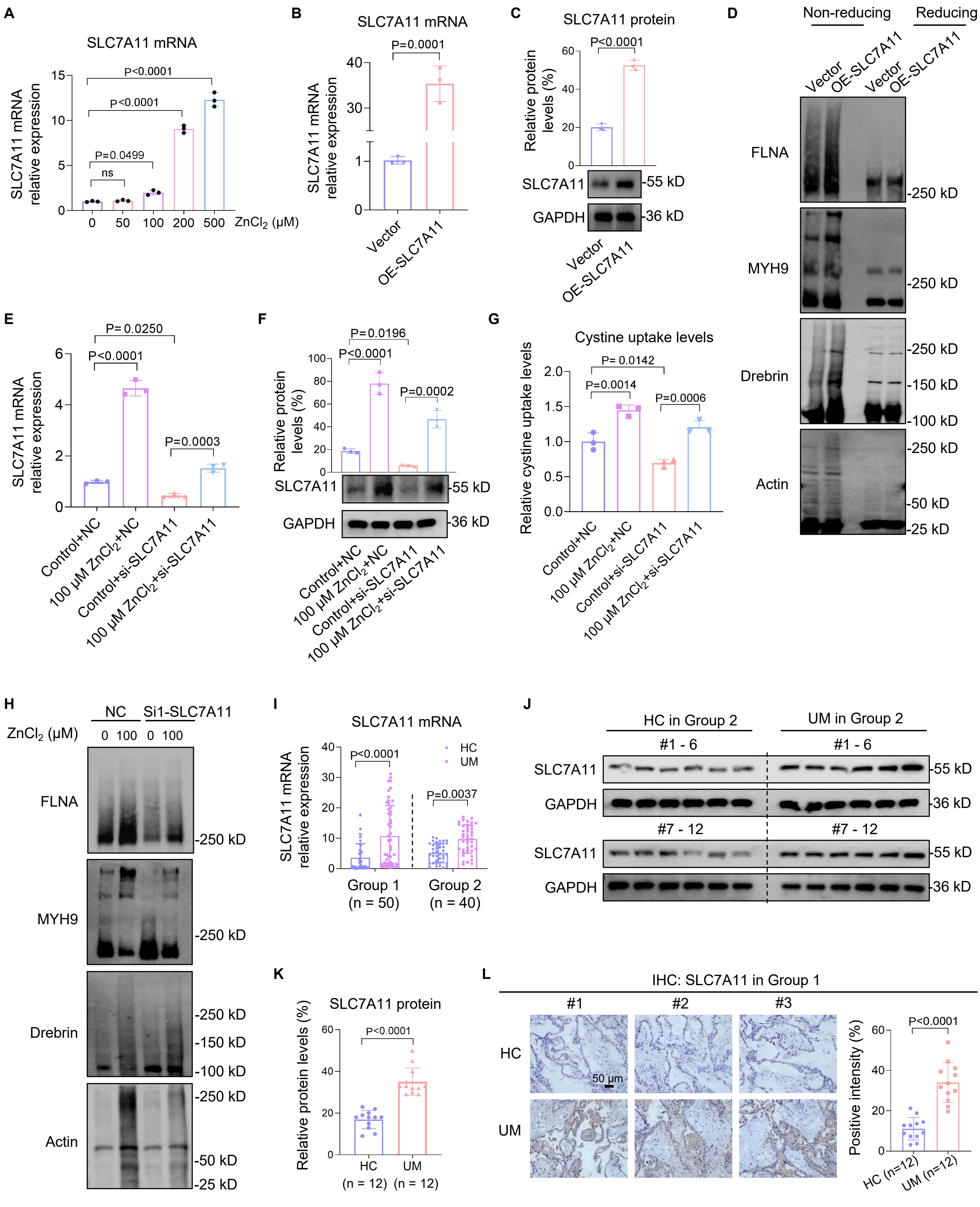
A
SLC7A11 mRNA
SLC7A11 mRNA relative expression
P<0.0001
P<0.0001
P=0.0499
ns
0 50 100 200 500 ZnCl2 (μM)
B
SLC7A11 mRNA
SLC7A11 mRNA relative expression
P=0.0001
Vector
OE-SLC7A11
C
SLC7A11 protein
Relative protein levels (%)
P<0.0001
SLC7A11
GAPDH
-55 kD
-36 kD
Vector
OE-SLC7A11
D
Non-reducing
Reducing
Vector
OE-SLC7A11
Vector
OE-SLC7A11
FLNA
MYH9
Drebrin
Actin
-250 kD
-250 kD
-250 kD
-150 kD
-100 kD
-250 kD
-50 kD
-25 kD
E
SLC7A11 mRNA relative expression
P= 0.0250
P<0.0001
P=0.0003
Control+NC
100 μM ZnCl2+NC
Control+si-SLC7A11
100 μM ZnCl2+si-SLC7A11
F
Relative protein levels (%)
P=0.0196
P<0.0001
P=0.0002
SLC7A11
GAPDH
-55 kD
-36 kD
Control+NC
100 μM ZnCl2+NC
Control+si-SLC7A11
100 μM ZnCl2+si-SLC7A11
G
Cystine uptake levels
Relative cystine uptake levels
P= 0.0142
P=0.0014
P=0.0006
Control+NC
100 μM ZnCl2+NC
Control+si-SLC7A11
100 μM ZnCl2+si-SLC7A11
H
NC
Si1-SLC7A11
ZnCl2 (μM)
0 100 0 100
FLNA
MYH9
Drebrin
Actin
-250 kD
-250 kD
-250 kD
-150 kD
-100 kD
-250 kD
-50 kD
-25 kD
I
SLC7A11 mRNA
SLC7A11 mRNA relative expression
HC
UM
P<0.0001
P=0.0037
Group 1 (n = 50)
Group 2 (n = 40)
J
HC in Group 2
UM in Group 2
#1 - 6
#1 - 6
#7 - 12
#7 - 12
SLC7A11
GAPDH
SLC7A11
GAPDH
-55 kD
-36 kD
-55 kD
-36 kD
K
SLC7A11 protein
Relative protein levels (%)
P<0.0001
HC (n = 12)
UM (n = 12)
L
IHC: SLC7A11 in Group 1
#1
#2
#3
HC
UM
50 μm
Positive intensity (%)
P<0.0001
HC (n=12)
UM (n=12)

Fig.S4-2

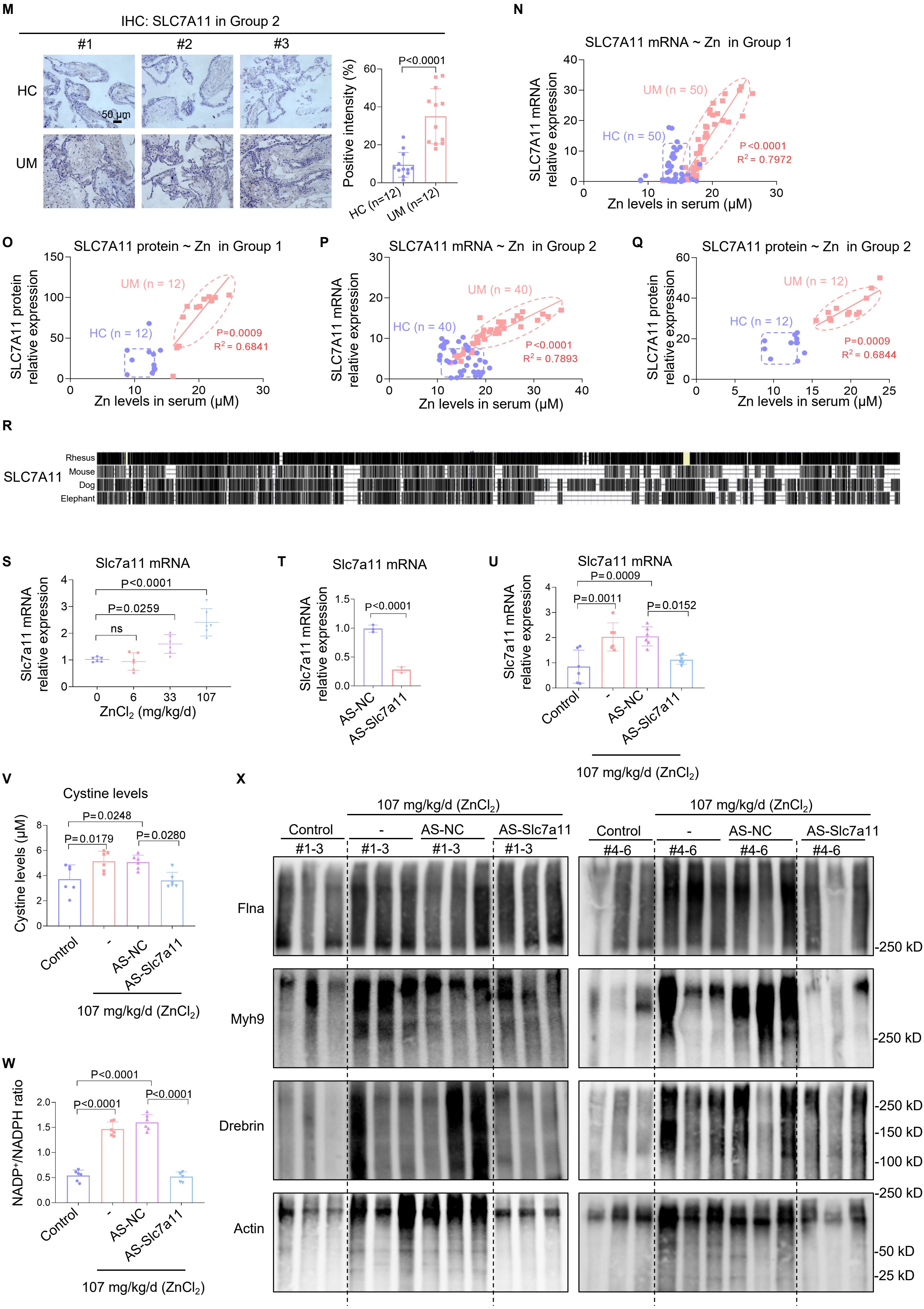
M
IHC: SLC7A11 in Group 2
#1
#2
#3
HC
UM
50 μm
Positive intensity (%)
P<0.0001
HC (n=12)
UM (n=12)
N
SLC7A11 mRNA ~ Zn in Group 1
SLC7A11 mRNA relative expression
UM (n = 50)
HC (n = 50)
P<0.0001
R2 = 0.7972
Zn levels in serum (μM)
O
SLC7A11 protein ~ Zn in Group 1
SLC7A11 protein relative expression
UM (n = 12)
HC (n = 12)
P=0.0009
R2 = 0.6841
Zn levels in serum (μM)
P
SLC7A11 mRNA ~ Zn in Group 2
SLC7A11 mRNA relative expression
UM (n = 40)
HC (n = 40)
P<0.0001
R2 = 0.7893
Zn levels in serum (μM)
Q
SLC7A11 protein ~ Zn in Group 2
SLC7A11 protein relative expression
UM (n = 12)
HC (n = 12)
P=0.0009
R2 = 0.6844
Zn levels in serum (μM)
R
SLC7A11
Rhesus
Mouse
Dog
Elephant
S
Slc7a11 mRNA
Slc7a11 mRNA relative expression
P<0.0001
P=0.0259
ns
ZnCl2 (mg/kg/d)
T
Slc7a11 mRNA
Slc7a11 mRNA relative expression
P<0.0001
AS-NC
AS-Slc7a11
U
Slc7a11 mRNA
Slc7a11 mRNA relative expression
P=0.0009
P=0.0011
P=0.0152
Control
-
AS-NC
AS-Slc7a11
107 mg/kg/d (ZnCl2)
V
Cystine levels
Cystine levels (μM)
P=0.0248
P=0.0179
P=0.0280
Control
-
AS-NC
AS-Slc7a11
107 mg/kg/d (ZnCl2)
W
NADP+/NADPH ratio
P<0.0001
P<0.0001
P<0.0001
Control
-
AS-NC
AS-Slc7a11
107 mg/kg/d (ZnCl2)
X
107 mg/kg/d (ZnCl2)
Control
-
AS-NC
AS-Slc7a11
#1-3
#4-6
Flna
Myh9
Drebrin
Actin
-250 kD
-150 kD
-100 kD
-50 kD
-25 kD

Fig.S5-1

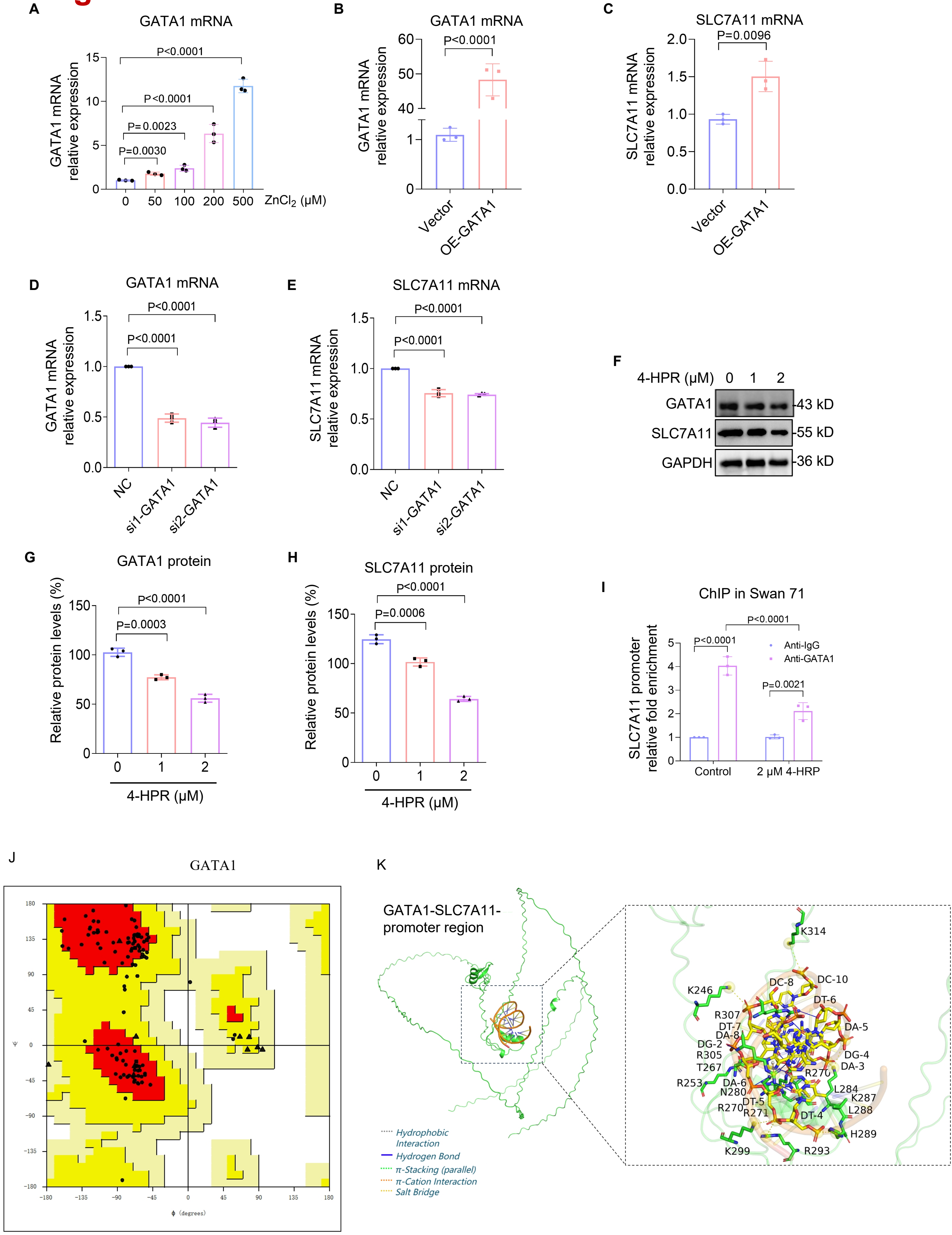
A
GATA1 mRNA
GATA1 mRNA relative expression
P<0.0001
P<0.0001
P= 0.0023
P=0.0030
0 50 100 200 500 ZnCl2 (μM)
B
GATA1 mRNA
GATA1 mRNA relative expression
P<0.0001
Vector
OE-GATA1
C
SLC7A11 mRNA
SLC7A11 mRNA relative expression
P=0.0096
Vector
OE-GATA1
D
GATA1 mRNA
GATA1 mRNA relative expression
P<0.0001
P<0.0001
NC
si1-GATA1
si2-GATA1
E
SLC7A11 mRNA
SLC7A11 mRNA relative expression
P<0.0001
P<0.0001
NC
si1-GATA1
si2-GATA1
F
4-HPR (μM) 0 1 2
GATA1
SLC7A11
GAPDH
43 kD
55 kD
36 kD
G
GATA1 protein
Relative protein levels (%)
P<0.0001
P=0.0003
4-HPR (μM)
H
SLC7A11 protein
Relative protein levels (%)
P<0.0001
P=0.0006
4-HPR (μM)
I
ChIP in Swan 71
SLC7A11 promoter relative fold enrichment
P<0.0001
P<0.0001
P=0.0021
Anti-IgG
Anti-GATA1
Control
2 μM 4-HRP
J
GATA1
Ψ
ϕ (degrees)
K
GATA1-SLC7A11-promoter region
K314
K246
DC-8
DC-10
DT-6
R307
DT-7
DA-8
DA-5
DG-2
R305
T267
DG-4
DA-3
R270
R253
DA-6
N280
L284
K287
R270
DT-5
R271
DT-4
L288
H289
K299
R293
Hydrophobic Interaction
Hydrogen Bond
π-Stacking (parallel)
π-Cation Interaction
Salt Bridge

Fig.S5-2

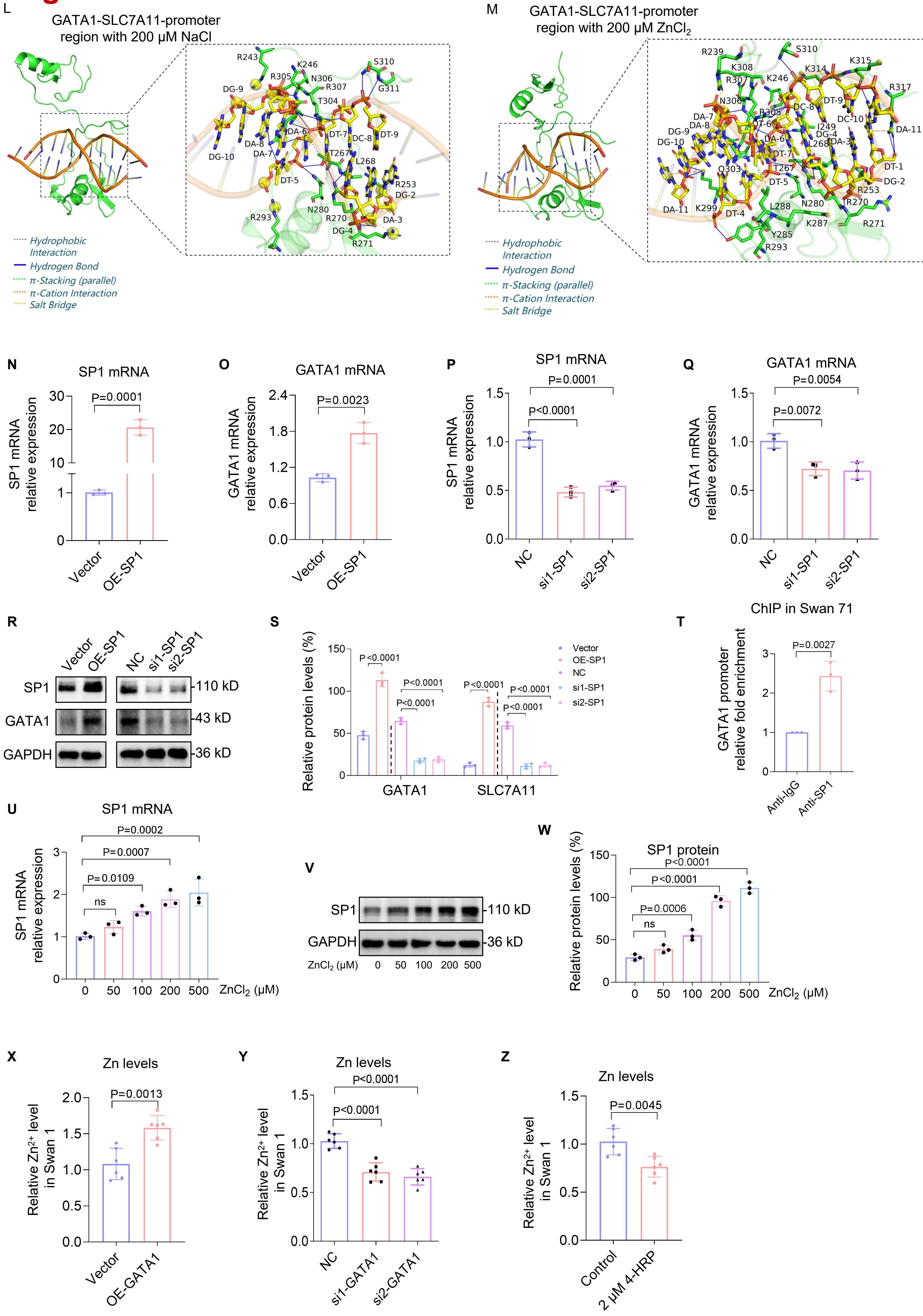
L
GATA1-SLC7A11-promoter region with 200 μM NaCl
R243
K246
S310
R305
N306
R307
G311
DG-9
T304
DA-6
DT-7
DC-8
DT-9
DG-10
DA-8
DA-7
T267
L268
DT-5
R253
DG-2
N280
R293
R270
DA-3
DG-4
R271
Hydrophobic Interaction
Hydrogen Bond
π-Stacking (parallel)
π-Cation Interaction
Salt Bridge
M
GATA1-SLC7A11-promoter region with 200 μM ZnCl2
R239
S310
K308
K314
K315
R307
K246
R317
N306
DT-9
R305
DC-8
DA-7
DA-8
DT-6
I249
DC-10
DG-9
DG-4
DA-11
DG-10
DA-6
L268
DA-3
DT-7
Q303
T267
DT-1
DT-5
DG-2
R253
N280
R270
DA-11
K299
DT-4
L288
K287
R271
Y285
R293
Hydrophobic Interaction
Hydrogen Bond
π-Stacking (parallel)
π-Cation Interaction
Salt Bridge
N
SP1 mRNA
SP1 mRNA relative expression
P=0.0001
Vector
OE-SP1
O
GATA1 mRNA
GATA1 mRNA relative expression
P=0.0023
Vector
OE-SP1
P
SP1 mRNA
SP1 mRNA relative expression
P=0.0001
P<0.0001
NC
si1-SP1
si2-SP1
Q
GATA1 mRNA
GATA1 mRNA relative expression
P=0.0054
P=0.0072
NC
si1-SP1
si2-SP1
R
Vector
OE-SP1
NC
si1-SP1
si2-SP1
SP1
GATA1
GAPDH
110 kD
43 kD
36 kD
S
Relative protein levels (%)
P<0.0001
P<0.0001
P<0.0001
P<0.0001
P<0.0001
P<0.0001
Vector
OE-SP1
NC
si1-SP1
si2-SP1
GATA1
SLC7A11
T
ChIP in Swan 71
GATA1 promoter relative fold enrichment
P=0.0027
Anti-IgG
Anti-SP1
U
SP1 mRNA
SP1 mRNA relative expression
P=0.0002
P=0.0007
P=0.0109
ns
ZnCl2 (μM)
V
SP1
GAPDH
110 kD
36 kD
ZnCl2 (μM)
0
50
100
200
500
W
SP1 protein
Relative protein levels (%)
P<0.0001
P<0.0001
P=0.0006
ns
ZnCl2 (μM)
X
Zn levels
Relative Zn2+ level in Swan 1
P=0.0013
Vector
OE-GATA1
Y
Zn levels
Relative Zn2+ level in Swan 1
P<0.0001
P<0.0001
NC
si1-GATA1
si2-GATA1
Z
Zn levels
Relative Zn2+ level in Swan 1
P=0.0045
Control
2 μM 4-HRP

Fig.S6-1

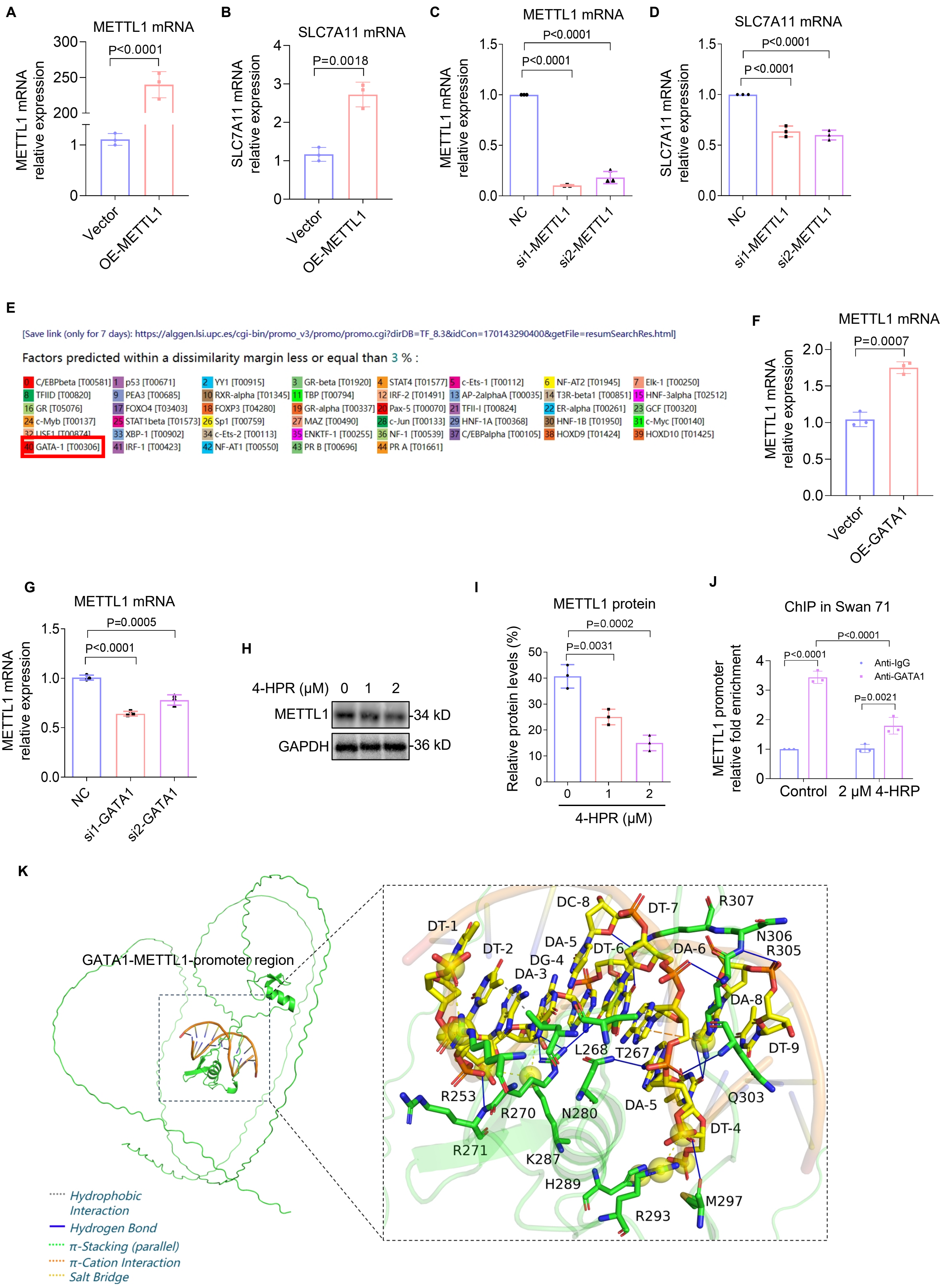

A
METTL1 mRNA
P<0.0001
METTL1 mRNA relative expression
300
250
200
1
0
Vector
OE-METTL1
B
SLC7A11 mRNA
P=0.0018
SLC7A11 mRNA relative expression
Vector
OE-METTL1
C
METTL1 mRNA
P<0.0001
P<0.0001
METTL1 mRNA relative expression
NC
si1-METTL1
si2-METTL1
D
SLC7A11 mRNA
P<0.0001
P<0.0001
SLC7A11 mRNA relative expression
NC
si1-METTL1
si2-METTL1
E
[Save link (only for 7 days): https://alggen.lsi.upc.es/cgi-bin/promo_v3/promo/promo.cgi?dirDB=TF_8.3&idCon=170143290400&getFile=resumSearchRes.html]
Factors predicted within a dissimilarity margin less or equal than 3 % :
0 C/EBPbeta [T00581] 1 p53 [T00671] 2 YY1 [T00915] 3 GR-beta [T01920] 4 STAT4 [T01577] 5 c-Ets-1 [T00112] 6 NF-AT2 [T01945] 7 Elk-1 [T00250]
8 TFIID [T00820] 9 PEA3 [T00685] 10 RXR-alpha [T01345] 11 TBP [T00794] 12 IRF-2 [T01491] 13 AP-2alphaA [T00035] 14 T3R-beta1 [T00851] 15 HNF-3alpha [T02512]
16 GR [T05076] 17 FOXO4 [T03403] 18 FOXP3 [T04280] 19 GR-alpha [T00337] 20 Pax-5 [T00070] 21 TFII-I [T00824] 22 ER-alpha [T00261] 23 GCF [T00320]
24 c-Myb [T00137] 25 STAT1beta [T01573] 26 Sp1 [T00759] 27 MAZ [T00490] 28 c-Jun [T00133] 29 HNF-1A [T00368] 30 HNF-1B [T01950] 31 c-Myc [T00140]
33 XBP-1 [T00902] 34 c-Ets-2 [T00113] 35 ENKTF-1 [T00255] 36 NF-1 [T00539] 37 C/EBPalpha [T00105] 38 HOXD9 [T01424] 39 HOXD10 [T01425]
40 GATA-1 [T00306] 41 IRF-1 [T00423] 42 NF-AT1 [T00550] 43 PR B [T00696] 44 PR A [T01661]
F
METTL1 mRNA
P=0.0007
METTL1 mRNA relative expression
Vector
OE-GATA1
G
METTL1 mRNA
P=0.0005
P<0.0001
METTL1 mRNA relative expression
NC
si1-GATA1
si2-GATA1
H
4-HPR (μM) 0 1 2
METTL1
-34 kD
GAPDH
-36 kD
I
METTL1 protein
P=0.0002
P=0.0031
Relative protein levels (%)
4-HPR (μM)
J
ChIP in Swan 71
P<0.0001
P<0.0001
P=0.0021
Anti-IgG
Anti-GATA1
METTL1 promoter relative fold enrichment
Control
2 μM 4-HRP
K
GATA1-METTL1-promoter region
DT-1
DT-2
DC-8
DT-7
R307
DA-5
DG-4
DA-3
DT-6
DA-6
N306
R305
DA-8
L268
T267
DT-9
R253
R270
N280
DA-5
Q303
DT-4
R271
K287
H289
R293
M297
Hydrophobic Interaction
Hydrogen Bond
π-Stacking (parallel)
π-Cation Interaction
Salt Bridge

Fig.S6-2

L

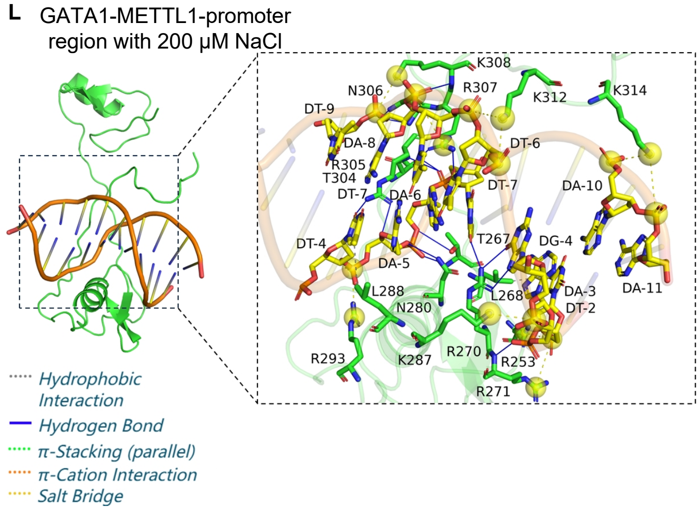
GATA1-METTL1-promoter region with 200 μM NaCl
K308
N306
R307
K312
K314
DT-9
DA-8
DT-6
R305
T304
DT-7
DA-6
DT-7
DA-10
DT-4
DA-5
T267
DG-4
L288
L268
DA-3
DA-11
N280
DT-2
R293
K287
R270
R253
R271
Hydrophobic Interaction
Hydrogen Bond
π-Stacking (parallel)
π-Cation Interaction
Salt Bridge

M

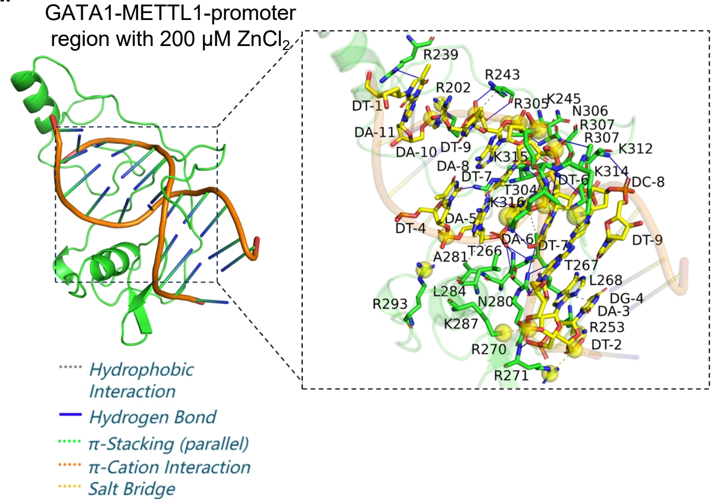
GATA1-METTL1-promoter region with 200 μM ZnCl2
R239
R243
R202
DT-1
R305
K245
N306
R307
DA-11
R307
K312
DA-10
DT-9
K315
DA-8
K314
DT-7
DT-6
DC-8
T304
K316
DA-5
DT-4
DA-6
DT-7
DT-9
A281
T266
T267
L268
L284
DG-4
R293
N280
DA-3
K287
R253
DT-2
R270
R271
Hydrophobic Interaction
Hydrogen Bond
π-Stacking (parallel)
π-Cation Interaction
Salt Bridge

N

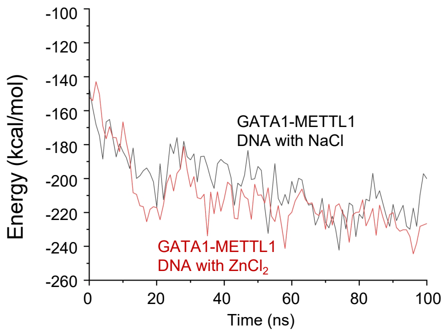
-100
-120
-140
-160
-180
-200
-220
-240
-260
Energy (kcal/mol)
GATA1-METTL1
DNA with NaCl
GATA1-METTL1
DNA with ZnCl2
0
20
40
60
80
100
Time (ns)

Fig.S7-1

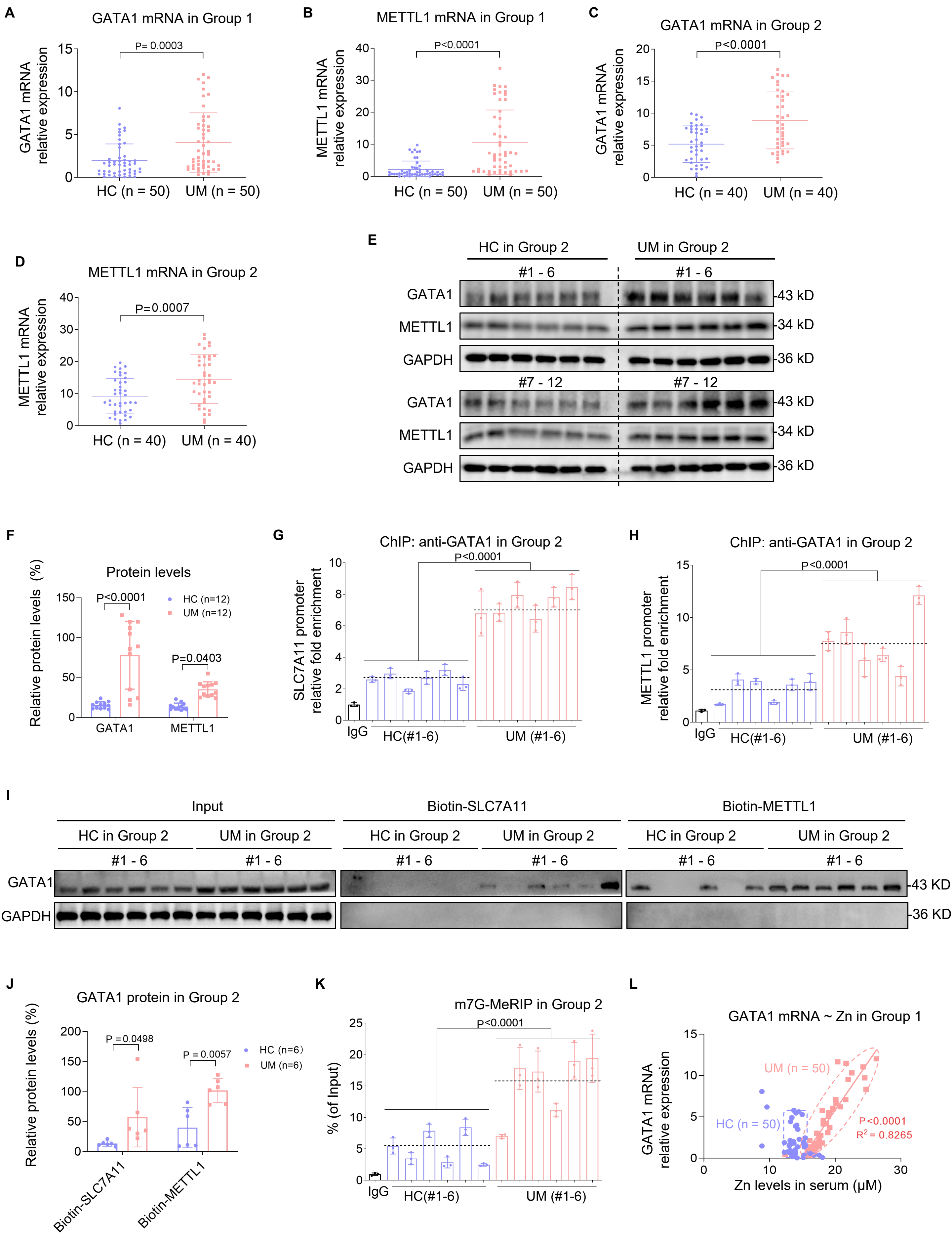
A
GATA1 mRNA in Group 1
GATA1 mRNA relative expression
P= 0.0003
HC (n = 50) UM (n = 50)
B
METTL1 mRNA in Group 1
METTL1 mRNA relative expression
P<0.0001
HC (n = 50) UM (n = 50)
C
GATA1 mRNA in Group 2
GATA1 mRNA relative expression
P<0.0001
HC (n = 40) UM (n = 40)
D
METTL1 mRNA in Group 2
METTL1 mRNA relative expression
P=0.0007
HC (n = 40) UM (n = 40)
E
HC in Group 2
UM in Group 2
#1 - 6
#7 - 12
GATA1
METTL1
GAPDH
-43 kD
-34 kD
-36 kD
F
Protein levels
Relative protein levels (%)
P<0.0001
P=0.0403
HC (n=12)
UM (n=12)
GATA1 METTL1
G
ChIP: anti-GATA1 in Group 2
SLC7A11 promoter relative fold enrichment
P<0.0001
IgG HC(#1-6) UM (#1-6)
H
ChIP: anti-GATA1 in Group 2
METTL1 promoter relative fold enrichment
P<0.0001
IgG HC(#1-6) UM (#1-6)
I
Input
Biotin-SLC7A11
Biotin-METTL1
HC in Group 2
UM in Group 2
#1 - 6
GATA1
GAPDH
-43 KD
-36 KD
J
GATA1 protein in Group 2
Relative protein levels (%)
P = 0.0498
P = 0.0057
HC (n=6)
UM (n=6)
Biotin-SLC7A11
Biotin-METTL1
K
m7G-MeRIP in Group 2
% (of Input)
P<0.0001
IgG HC(#1-6) UM (#1-6)
L
GATA1 mRNA ~ Zn in Group 1
GATA1 mRNA relative expression
UM (n = 50)
HC (n = 50)
P<0.0001
R² = 0.8265
Zn levels in serum (μM)

Fig.S7-2

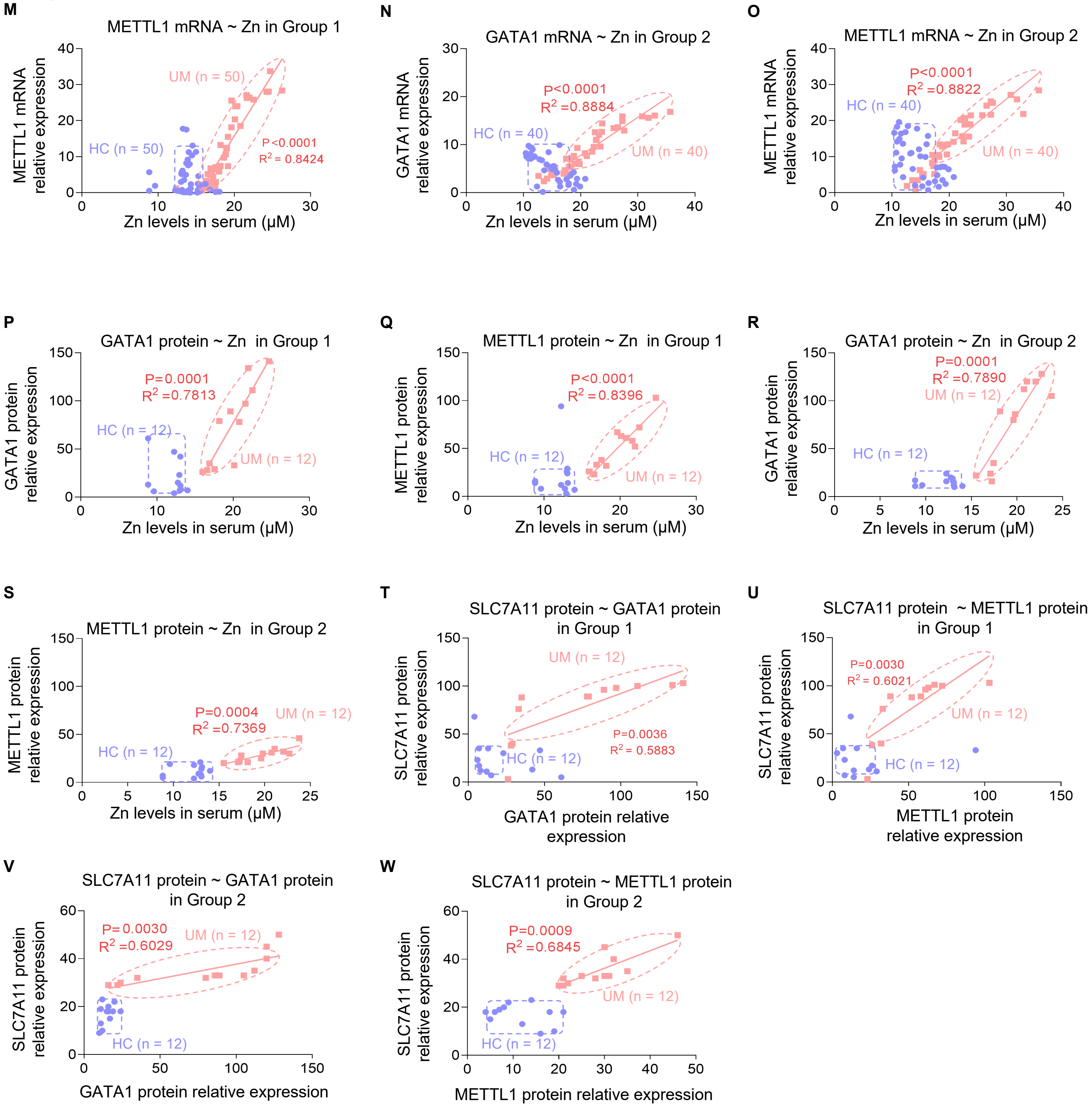
M
METTL1 mRNA ~ Zn in Group 1
METTL1 mRNA relative expression
Zn levels in serum (μM)
UM (n = 50)
HC (n = 50)
P<0.0001
R2 = 0.8424
N
GATA1 mRNA ~ Zn in Group 2
GATA1 mRNA relative expression
Zn levels in serum (μM)
P<0.0001
R2 =0.8884
HC (n = 40)
UM (n = 40)
O
METTL1 mRNA ~ Zn in Group 2
METTL1 mRNA relative expression
Zn levels in serum (μM)
P<0.0001
R2 =0.8822
HC (n = 40)
UM (n = 40)
P
GATA1 protein ~ Zn in Group 1
GATA1 protein relative expression
Zn levels in serum (μM)
P=0.0001
R2 =0.7813
HC (n = 12)
UM (n = 12)
Q
METTL1 protein ~ Zn in Group 1
METTL1 protein relative expression
Zn levels in serum (μM)
P<0.0001
R2 =0.8396
HC (n = 12)
UM (n = 12)
R
GATA1 protein ~ Zn in Group 2
GATA1 protein relative expression
Zn levels in serum (μM)
P=0.0001
R2 =0.7890
UM (n = 12)
HC (n = 12)
S
METTL1 protein ~ Zn in Group 2
METTL1 protein relative expression
Zn levels in serum (μM)
P=0.0004
R2 =0.7369
UM (n = 12)
HC (n = 12)
T
SLC7A11 protein ~ GATA1 protein in Group 1
SLC7A11 protein relative expression
GATA1 protein relative expression
UM (n = 12)
HC (n = 12)
P=0.0036
R2 = 0.5883
U
SLC7A11 protein ~ METTL1 protein in Group 1
SLC7A11 protein relative expression
METTL1 protein relative expression
P=0.0030
R2 = 0.6021
UM (n = 12)
HC (n = 12)
V
SLC7A11 protein ~ GATA1 protein in Group 2
SLC7A11 protein relative expression
GATA1 protein relative expression
P= 0.0030
R2 =0.6029
UM (n = 12)
HC (n = 12)
W
SLC7A11 protein ~ METTL1 protein in Group 2
SLC7A11 protein relative expression
METTL1 protein relative expression
P=0.0009
R2 =0.6845
UM (n = 12)
HC (n = 12)

Fig.S8

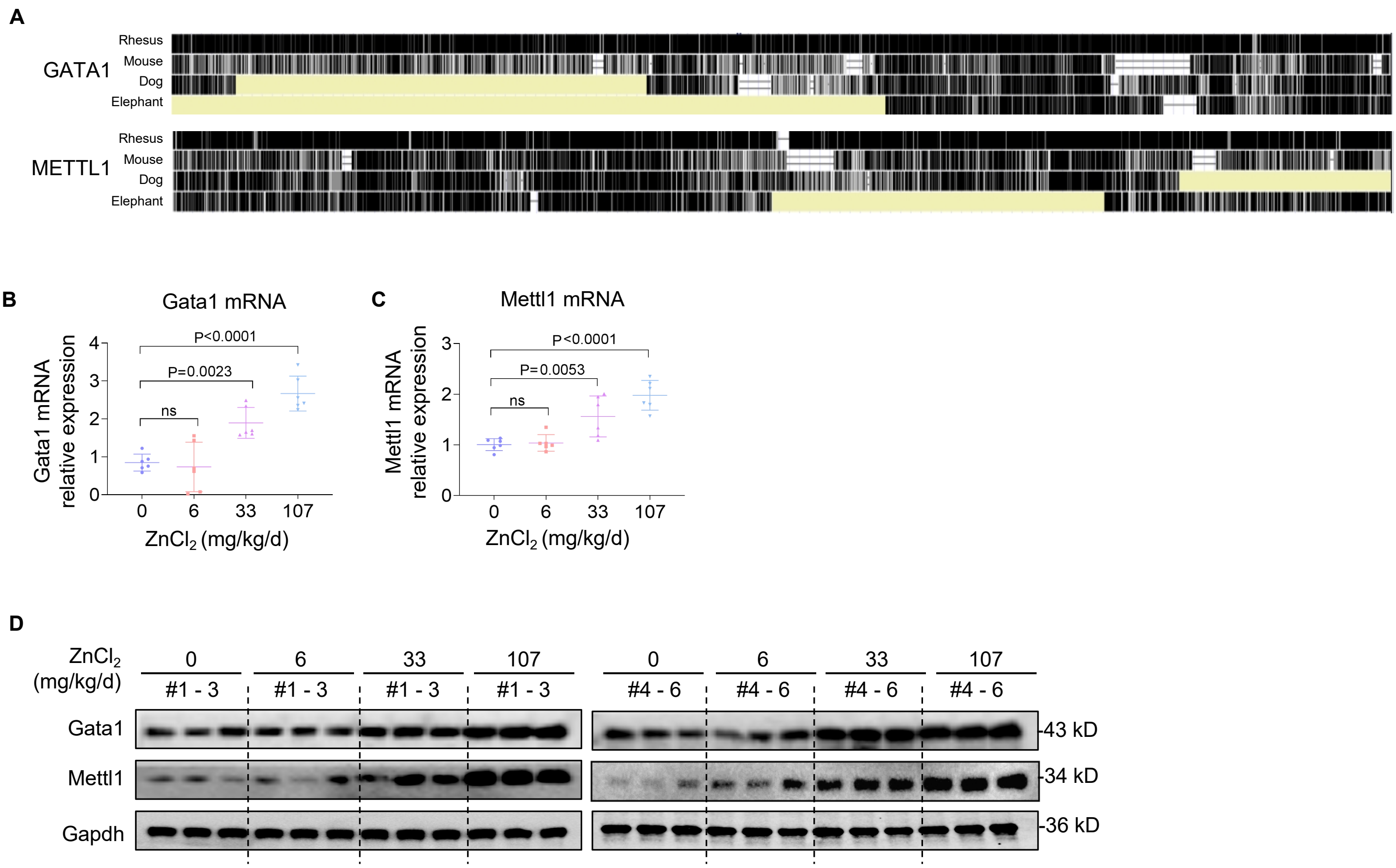

A
GATA1
Rhesus
Mouse
Dog
Elephant
METTL1
Rhesus
Mouse
Dog
Elephant
B
Gata1 mRNA
Gata1 mRNA relative expression
P<0.0001
P=0.0023
ns
0
6
33
107
ZnCl2 (mg/kg/d)
C
Mettl1 mRNA
Mettl1 mRNA relative expression
P<0.0001
P=0.0053
ns
0
6
33
107
ZnCl2 (mg/kg/d)
D
ZnCl2 (mg/kg/d)
0
6
33
107
#1 - 3
#4 - 6
Gata1
Mettl1
Gapdh
43 kD
34 kD
36 kD

Fig.S9-1

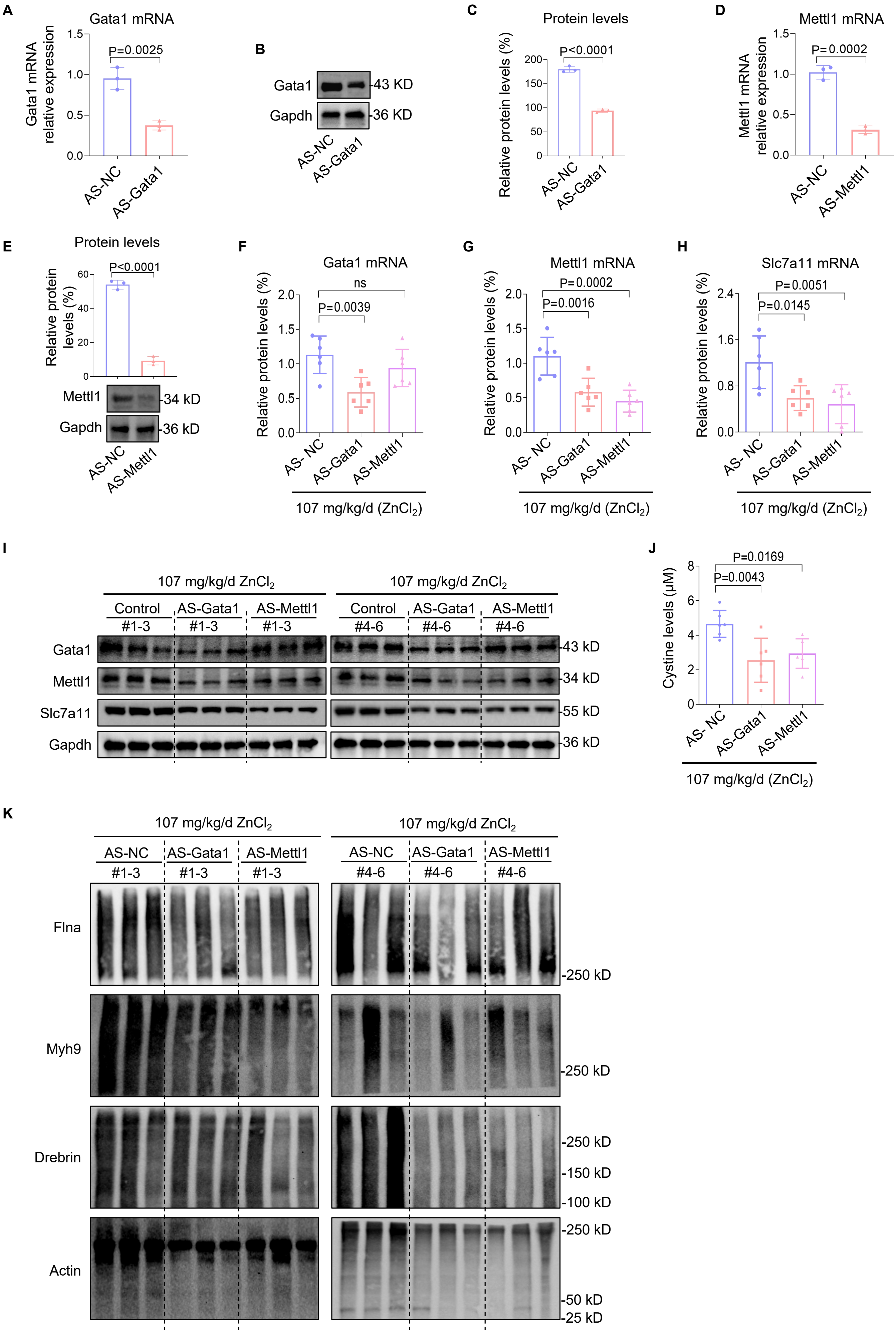

A
Gata1 mRNA
Gata1 mRNA relative expression
P=0.0025
AS-NC
AS-Gata1
B
Gata1
Gapdh
43 KD
36 KD
AS-NC
AS-Gata1
C
Protein levels
Relative protein levels (%)
P<0.0001
AS-NC
AS-Gata1
D
Mettl1 mRNA
Mettl1 mRNA relative expression
P=0.0002
AS-NC
AS-Mettl1
E
Protein levels
Relative protein levels (%)
P<0.0001
Mettl1
Gapdh
34 kD
36 kD
AS-NC
AS-Mettl1
F
Gata1 mRNA
Relative protein levels (%)
ns
P=0.0039
AS- NC
AS-Gata1
AS-Mettl1
107 mg/kg/d ($ZnCl_2$)
G
Mettl1 mRNA
Relative protein levels (%)
P=0.0002
P=0.0016
AS- NC
AS-Gata1
AS-Mettl1
107 mg/kg/d ($ZnCl_2$)
H
Slc7a11 mRNA
Relative protein levels (%)
P=0.0051
P=0.0145
AS- NC
AS-Gata1
AS-Mettl1
107 mg/kg/d ($ZnCl_2$)
I
107 mg/kg/d $ZnCl_2$
Control #1-3
AS-Gata1 #1-3
AS-Mettl1 #1-3
Control #4-6
AS-Gata1 #4-6
AS-Mettl1 #4-6
Gata1
Mettl1
Slc7a11
Gapdh
43 kD
34 kD
55 kD
36 kD
J
Cystine levels (μM)
P=0.0169
P=0.0043
AS- NC
AS-Gata1
AS-Mettl1
107 mg/kg/d ($ZnCl_2$)
K
107 mg/kg/d $ZnCl_2$
AS-NC #1-3
AS-Gata1 #1-3
AS-Mettl1 #1-3
AS-NC #4-6
AS-Gata1 #4-6
AS-Mettl1 #4-6
Flna
Myh9
Drebrin
Actin
250 kD
150 kD
100 kD
50 kD
25 kD

Fig.S9-2

L

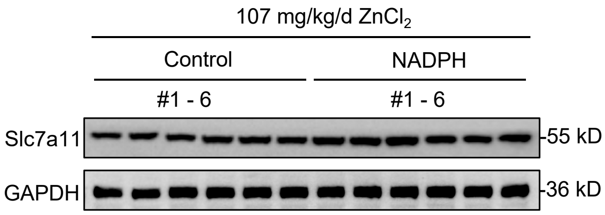
107 mg/kg/d $ZnCl_2$
Control
NADPH
#1 - 6
#1 - 6
Slc7a11
GAPDH
-55 kD
-36 kD

M

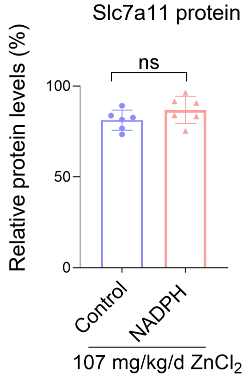
Slc7a11 protein
Relative protein levels (%)
ns
100
50
0
Control
NADPH
107 mg/kg/d $ZnCl_2$

N

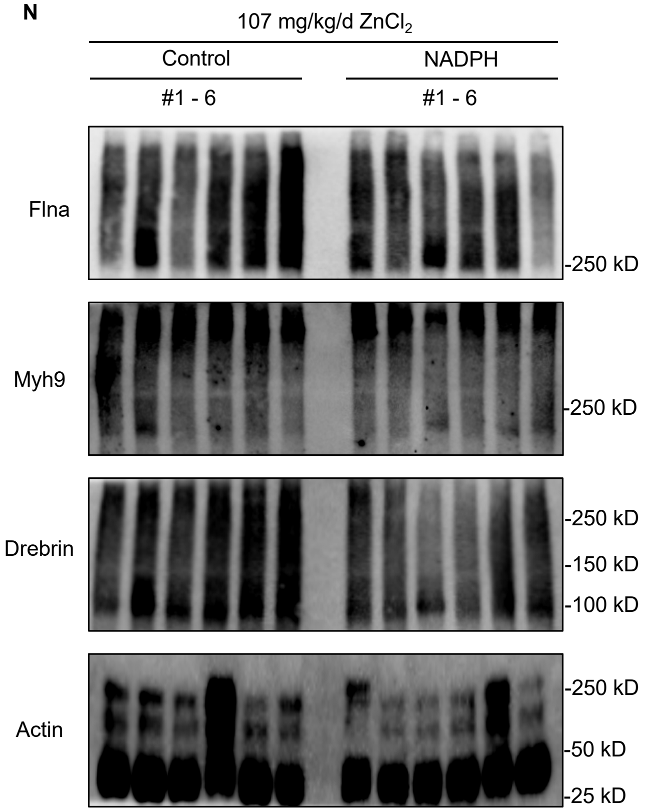
107 mg/kg/d $ZnCl_2$
Control
NADPH
#1 - 6
#1 - 6
Flna
Myh9
Drebrin
Actin
-250 kD
-250 kD
-250 kD
-150 kD
-100 kD
-250 kD
-50 kD
-25 kD

O

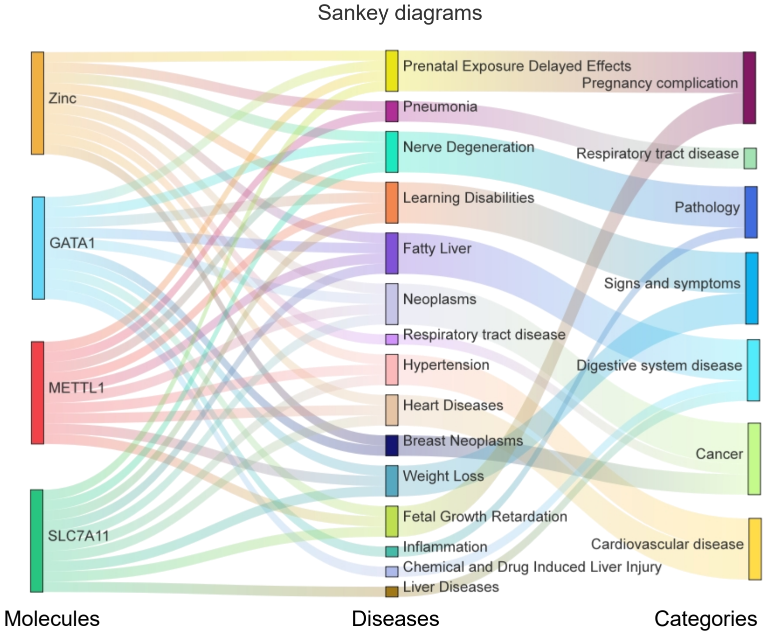
Sankey diagrams
Zinc
GATA1
METTL1
SLC7A11
Prenatal Exposure Delayed Effects
Pneumonia
Nerve Degeneration
Learning Disabilities
Fatty Liver
Neoplasms
Respiratory tract disease
Hypertension
Heart Diseases
Breast Neoplasms
Weight Loss
Fetal Growth Retardation
Inflammation
Chemical and Drug Induced Liver Injury
Liver Diseases
Pregnancy complication
Respiratory tract disease
Pathology
Signs and symptoms
Digestive system disease
Cancer
Cardiovascular disease
Molecules
Diseases
Categories

Fig.S10

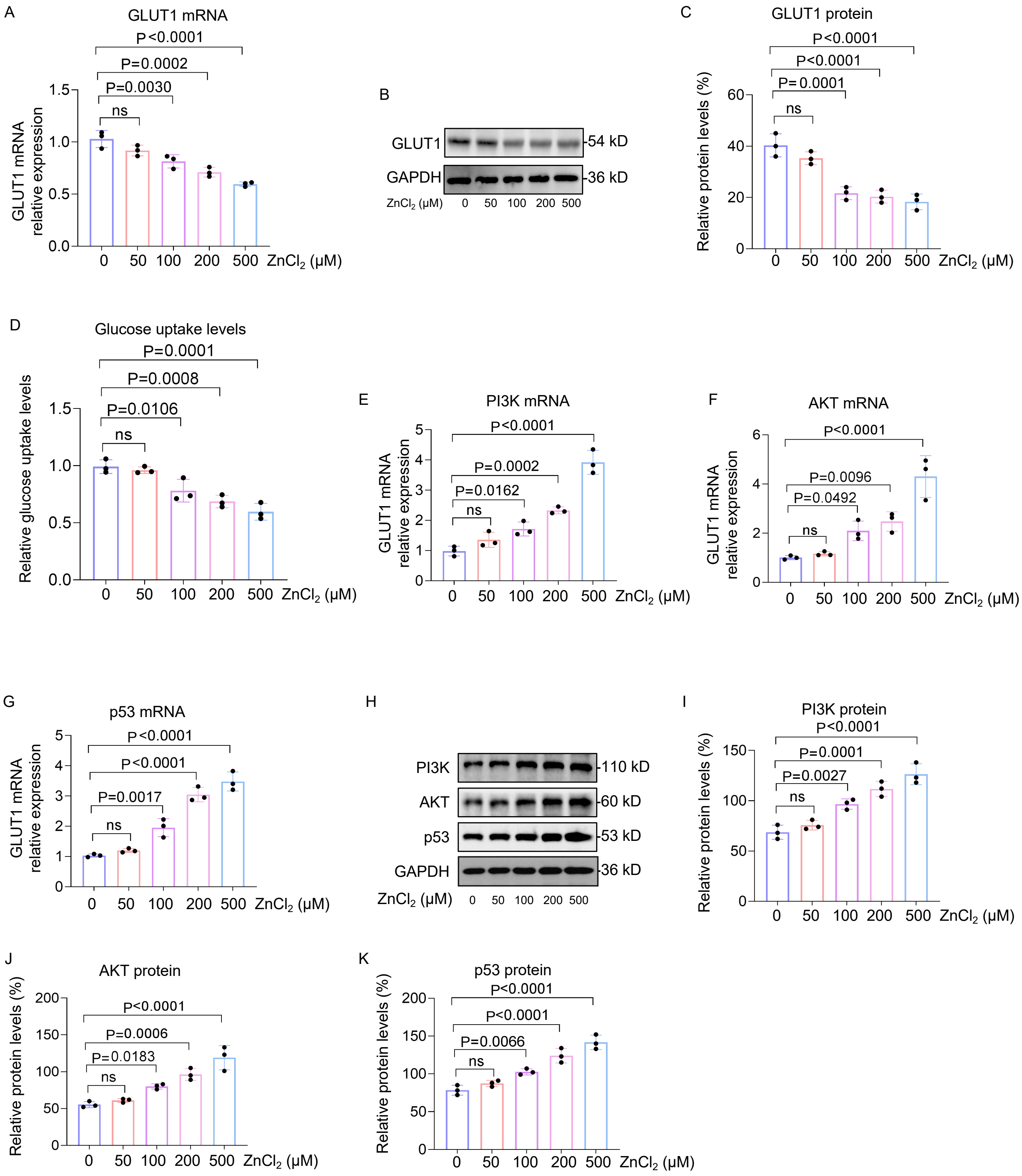
A
GLUT1 mRNA
P<0.0001
P=0.0002
P=0.0030
ns
GLUT1 mRNA relative expression
0 50 100 200 500 ZnCl2 (μM)
B
GLUT1
54 kD
GAPDH
36 kD
ZnCl2 (μM) 0 50 100 200 500
C
GLUT1 protein
P<0.0001
P<0.0001
P= 0.0001
ns
Relative protein levels (%)
0 50 100 200 500 ZnCl2 (μM)
D
Glucose uptake levels
P=0.0001
P=0.0008
P=0.0106
ns
Relative glucose uptake levels
0 50 100 200 500 ZnCl2 (μM)
E
PI3K mRNA
P<0.0001
P=0.0002
P=0.0162
ns
GLUT1 mRNA relative expression
0 50 100 200 500 ZnCl2 (μM)
F
AKT mRNA
P<0.0001
P=0.0096
P=0.0492
ns
GLUT1 mRNA relative expression
0 50 100 200 500 ZnCl2 (μM)
G
p53 mRNA
P<0.0001
P<0.0001
P=0.0017
ns
GLUT1 mRNA relative expression
0 50 100 200 500 ZnCl2 (μM)
H
PI3K
110 kD
AKT
60 kD
p53
53 kD
GAPDH
36 kD
ZnCl2 (μM) 0 50 100 200 500
I
PI3K protein
P<0.0001
P=0.0001
P=0.0027
ns
Relative protein levels (%)
0 50 100 200 500 ZnCl2 (μM)
J
AKT protein
P<0.0001
P=0.0006
P=0.0183
ns
Relative protein levels (%)
0 50 100 200 500 ZnCl2 (μM)
K
p53 protein
P<0.0001
P<0.0001
P=0.0066
ns
Relative protein levels (%)
0 50 100 200 500 ZnCl2 (μM)
